# Supplementary material for: Beyond the facility: An evaluation of seven community-based pediatric HIV testing strategies and linkage to care outcomes in a high prevalence, resource-limited setting
Source: PLoS One. 2020 Sep 2;15(9):e0236985. doi: 10.1371/journal.pone.0236985 (PMC7467225; doi:10.1371/journal.pone.0236985)
Supplement: S1 File — (PDF) [file pone.0236985.s003.pdf]

NATIONAL GUIDELINES ON THE USE OF ANTIRETROVIRAL THERAPY FOR HIV PREVENTION AND TREATMENT

(FourthEdition)

BOPHELO

Ministry of Health

Government of Lesotho

December 2013

TABLE OF CONTENTS

Table of Contents i

Abbreviations and Acronyms iii

Foreword v

[Honourable Minister of Health]Acknowledgements vi

Executive Summary viii

CHAPTER 1: INTRODUCTION 1

CHAPTER 2: HIV DIAGNOSIS 2

Section 2.1: HIV Testing 2
Section 2.2: HIV Diagnosis in Children 6
Section 2.3: Diagnosis of HIV in Adults and Children ≥ 18 Months 11
Section 2.4: Documentation of Test Results 12

CHAPTER 3: HIV CARE AND TREATMENT 13

Section 3.1: HIV Care and Treatment Package 13
Section 3.2: HIV Care and Treatment Package 15
Section 3.3: Baseline Laboratory Investigations 21
Section 3.4: Primary and Supportive Care 22
Section 3.5: Preventing Transmission of HIV among PLHIV 24
Section 3.6: Care of the HIV-Exposed Infant 24
Section 3.7: Selecting Patients for Antiretroviral Therapy 26
Section 3.8: Adults and Children Not Yet Eligible for ART 28

CHAPTER 4: ANTIRETROVIRAL TREATMENT REGIMENS 29

Section 4.1: First line Regimens 29
Section 4.2: Second-line Regimens 32
Section 4.3: Third-line Regimens 33

CHAPTER 5: PATIENT MONITORING 34

Section 5.1: Clinical Monitoring 34
Section 5.2: Laboratory Monitoring 36
Section 5.3: Monitoring and Substitutions for Arv Drug Toxicities 40
Section 5.4: Drug-Drug Interactions 41

CHAPTER 6: ADHERENCE AND DISCLOSURE 43

Section 6.1: Introduction 43
Section 6.2: Adherence Preparation 43
Section 6.3: Maintaining Adherence 46
Section 6.4: Disclosure 48

CHAPTER 7: Opportunistic AND CO-INFECTIONS 52

Section 7.1: Management of Opportunistic and Co-infections 52
Section 7.2: Tuberculosis 55
Section 7.3: Management of Cryptococcal Disease 63
Section 7.4: *Pneumocytis jirovecii* Pneumonia (PCP) 65
Section 7.5: Other Hiv- Associated Illnesses 66
Section 7.6: Mental Health Problems in Relation to Hiv 67
Section 7.7: Malignancy and HIV 69

CHAPTER 8: NUTRITION and HIV 74

CHAPTER 9: WELLNESS INFORMATION 80

CHAPTER 10: POST EXPOSURE PROPHYLAXIS (PEP) 82

Section 10.1: Introduction 82
Section 10.2: Indications for PEP 82
Section 10.3: Providing PEP 83

CHAPTER 11: INFECTION CONTROL 87

CHAPTER 12: OPERATIONAL AND SERVICE DELIVERY 91

Section 12.1: Decentralization of HIV Care and Treatment 91
section 12.2: Retention Across the Continuum of Care 92
Section 12.3: Service Integration and Linkages 93
Section 12.4: Human Resources 94
Section 12.5: Laboratory and Diagnostic Services 95
Section 12.6: Procurement and Supply Management System 95

CHAPTER 13: PROGRAMME MONITORING AND EVALUATION 97

Section 13.1: Definitions 97
Section 13.2: Overview of the Patient Monitoring System 97
Section 13.3: Monitoring Implications of 2013 recommendations 99
Section 13.4: Other Monitoring Considerations 99

ANNEXES: 102

Annex 1: Developmental Milestones in Infants and Young Children 102
Annex 2: Developmental Red Flags 103
Annex 3: Gross Motor Milestones in Infants and Young Children 104
Annex 4: Weight-based Dosing of Antiretrovirals and Prophylactics 105
Annex 5: Talking About HIV to HIV-Infected Children 107
Annex 6: Grading of ARV Toxicities 109
Annex 7: HIV Chronic Care/ART REFERRAL FORM 112
Annex 8: Adherence Contract 113
Annex 9: Drug-Drug Interactions 118
Annex 10: How to Analyse Indicators and Identify Problems 120
Annex 11: Weight-Based Dosing of Anti-TB Fixed-Dose Combination Medications 125
Annex 12: Reference Values for Weight-for-Height and Weight-for-Length 126
Annex 13: Ready-to-Use Therapeutic Food (RUTF) Dosing 128

ABBREVIATIONS AND ACRONYMS

ABC	Abacavir
AFASS	Affordable, Feasible, Acceptable, Sustainable and Safe
AFB	Acid-fast bacilli
AIDS	Acquired Immune Deficiency Syndrome
ALT	Alanine aminotransferase
ANC	Antenatal care
ART	Antiretroviral therapy
ARV	Antiretroviral
ATT	Anti tuberculosis treatment
ATV	Atazanavir
AZT	Azidothymidine, also known as Zidovudine
CDC	Centres for Disease Control and Prevention
CSF	Cerebro-Spinal Fluid
CTC	Care and Treatment Clinics
CTX	Co-trimoxazole
CXR	Chest X-ray
d4T	Stavudine
DBS	DriedBlood Spot
DNA	Deoxyribonucleic Acid
DST	Drug Susceptibility Testing
EC	Emergency Contraceptive
EFV	Efavirenz
ELISA	Enzyme-linked immunosorbent assay
EPTB	Extra-pulmonary tuberculosis
ETV	Etravirine
FBC	Full Blood Count
FP	Family Planning
HCW	Healthcare worker
Hb	Haemoglobin
HBsAg	Hepatitis B surface antigen
HCV	Hepatitis C Virus
HIV	Human Immunodeficiency Virus
HPV	Human Papilloma Virus
HRZE	Isoniazid, Rifampicin, Pyrazinamide, Ethambutol
HTC	HIV Testing and Counselling
IPT	Isoniazid Prophylaxis Therapy
IUD	Intra-Uterine Device
LFTs	Liver Function Tests
LPV/r	Lopinavir/Ritonavir boosted
MCH	Maternal and Child Health
MDR-TB	Multi-Drug Resistant TB
MOH	Ministry of Health
MTCT	Mother-to-Child Transmission of HIV
MUAC	Mid-Upper-Arm Circumference
NGO	Non-Governmental Organisation
NVP	Nevirapine
NRTI	Nucleoside Reverse Transcriptase Inhibitor
NtRTI	Nucleotide Reverse Transcriptase Inhibitor
NNRTI	Non-nucleoside Reverse Transcriptase Inhibitor

NSAIDs	Non-steroidal Anti-Inflammatory Drugs
OI	Opportunistic Infection
PCP	*Pneumocystis jirovecci* Pneumonia
PCR	Polymerase Chain Reaction
PEP	Post-Exposure Prophylaxis
PLHIV	People Living With HIV
PMTCT	Prevention of Mother-to-child Transmission of HIV
RAL	Raltegravir
RH	Rifampicin, Isoniazid
RPR	Rapid plasma regain (syphilis test)
RTV	Ritonavir
RUTF	Ready-to-use Therapeutic Food
SJS	Stevens-Johnson Syndrome
STI	Sexually Transmitted Infection
TB	Tuberculosis
3TC	Lamivudine
TDF	Tenofovir Disoproxil Fumatrate
TLC	Total Lymphocyte Count
TNA	Trained Nurse Assistant
VDRL	Venereal Disease Research Laboratory (syphilis test)
VHW	Village Health Worker
WBC	White Blood Count
WFP	World Food Programme
WHO	World Health Organization
XDR-TB	Extensively Drug Resistant TB
ZDV	Zidovudine; also known as Azidothymidine (AZT)

FOREWORD

The government of Lesotho has once again seized the opportunities offered by the WHO 2013 consolidated guidelines, to renew its commitment to reversing the scourge of the HIV and AIDS epidemic in the country. In 2004, the government through the Ministry of Health made a momentous decision to provide HIV comprehensive care and treatment in the public sector, and to use nurse base service delivery modelsfor scaling up antiretroviral therapy for the Basotho. Since then, the country continues in the forefront to adapt and scale up the most cost effective interventions to improve the quality of life of the people. By November 2007, the CD4 criteria for initiating patients on highly active antiretroviral treatment, was increased from 200 to 350 cells per mm^3. In addition, since 2010, Tenofovir based regimen is used as preferred first line, including phased up use of a single fixed-dose combination pill taken once per day. Furthermore, since 2012, highly active antiretroviral treatment is offered to all HIV infected pregnant women to prevent mother to child HIV transmission and provide early treatment for their own health.

The fourth edition of these guidelines is yet another ambitious commitment of the government to achieve and contribute to the global target of reducing and reversing the HIV and AIDS epidemic trend by 2015. Lesotho has the 2nd highest prevalence of HIV in the world. In 2013, the estimated number of people infected with HIV in need of ART was 160,200 and of whom 101,000(56%) patients were receiving the lifesaving treatment by December 2013, based on the 2010 national guidelines, which recommend 350 CD4 cut of point. The government of Lesotho through the Ministry of Health has made the commitment to provide 54% of its targeted 282,400 with ART by 2016, based on the 350 CD4 cut of point. The current guidelines have moved the bar higher to CD4 500 cut off point of Less or equal to 500, increasing the number of people in need of treatment.

10 years of experience in providing treatment to the sickest and aiming at ever increasing targets, has built the capacity and infrastructure to make services available, even to the poor and very remote areas. By the end of 2013, all public health facilities provide free safer and more robust treatment. Innovative strategies such as integration of ART for HIV positive pregnant and lactating women and their infants in maternal and child health services, integrated outreach community services and use of community health workers to provide HIV testing, counselling and adherence support and referral, allow for early diagnosis and treatment monitoring in the country'sremotest areas.

Even if by end of 2013 close to 50% of those who need treatment in Lesotho did not receive it, the government will forge ahead to reap the dual benefits of early treatment: keeping people healthier and reducing transmission of HIV to others. The guidelines therefore promote treatment, using a single one day pill for all HIV pregnant women, under five year old children, all HIV infected adults with Tuberculosis, Hepatitis, HIV positive sexual partners in a discordant relationship, and CD4 less than 500. This, we believe will simplify the operational demands on programmes and encourage innovations to reach all who need treatment.

The guidelines require each and every one, including the community and affected people to do business differently. We must make conscious and innovative changes,

invest wisely, which according to our National HIV Strategy and investment case strategy could avert 21,000 deaths and prevent around 32,000 new infections by 2016.

Lesotho is a resource challenged country but with a high burden of TB and HIV. I am personally convinced that nurturing our past experience will promote scale up implementing and adhering to these guidelines and utilize efficiently limited resources towards attaining the “Universal Access” commitment made by the country.

On behalf of all Basotho, I thank those that contributed to the development of this document.

Dr. Pinkie Rose Marie MANAMOLELA

HONOURABLE MINISTER OF HEALTH

ACKNOWLEDGEMENTS

The review of the National ART guidelines has been a joint effort between the Ministry of Health (MOH) and diverse stakeholders. The joint efforts have generated the data and information useful in making informed decision on interventions that Lesotho should invest in for better health outcomes. The Ministry wishes to acknowledge the valuable support and contribution of the different stakeholders and development partners who participated in the review and the revision of the National ART guidelines possible.

In particular, the Ministry wishes to express its special thanks and appreciation to the Ministry of Health and CHAL health facilities in Mokhotlong, Butha Buthe and Semonkong that were visited by the team of consultants and ART programme staff during the field visits. The ART TWG that provided technical support and policy oversight. The Ministry further wishes to thank all the organisations and individuals who have participated in the stakeholders meetings during review and the revision of the National ART Guidelines. Their participation ensured the quality, technical soundness and comprehensiveness of the revised guidelines.

Special thanks goes to World Health Organization (WHO) for providing the financial and technical support to the process. The Government further wishes to acknowledge the technical backstopping by WHO country office and WHO AFRO.

The Government is grateful to the team of consultants who facilitated the process and put together the revised ART guidelines and the 3-year ART Implementation framework 2014-2016. The team of consultants composed of Dr Tsitsi Appollo - International consultant, and Dr. Limpho Maile – Local Consultant. Without their dedication and commitment the revised ART guidelines and the ART Implementation Framework would not have been possible.

Finally I want to express my sincere gratitude to the staff of the Disease Control Directorate and in particular Ms. Puleng Ramphalla-Phatela, Manager of STI, HIV and AIDS programme, Ms. Annah Masheane, TB/HIV Clinical Officer, Ms. Matšitso Mohoanyane for their special commitment and tireless efforts in ensuring that the success of the project. They worked beyond the call of duty to make sure we the guidelines were updated.

Dr. Moselinyane Letsie
Director Disease Control

EXECUTIVE SUMMARY

While the global response to HIV and AIDS has resulted in a decrease in the overallprevalence of infection, the epidemic continues to have devastating effects on thecommunities affected. According to the most recent UNAIDS report, there were anestimated 35.3 million people living with HIV, 3.3 million of whom were children; 2.3million people were newly infected and 1.6 million people who died from AIDS in2012.

At 23%, Lesotho has the second highest adult HIV prevalence in the world. Thereare an estimated 62 new HIV infections and about 50 deaths due to AIDS each day.An estimated 380,000 people are living with HIV in Lesotho. Of these, 38,000 arechildren and 342,000 are adults. There has been no significant change in thenational adult HIV prevalence since 2005. The sentinel surveillance conducted in2013 showed that there is no major difference in HIV prevalence among womenattending ANC clinics. The prevalence stands at 25.9%. Mafeteng, Mohale's Hoek and Maseru Districts reported the highest HIV prevalence at 35.8%, 30.6% and 29.5% respectively whereas Butha Buthe had the lowest prevalence at 17.4%.

The general pattern suggested increasing HIV prevalence with increasing age up to the 35-39 age group which had the highest 44.0% (n=107/243) prevalence followed by 30-34yrs age group at 40.8%(n=232/568). The lowest HIV prevalence 10.9% was among teenagers aged between 15 and 19yrs. HIV prevalence among 20-24 and 15-24 year age groups was 22.4% and 18.0% respectively. The sentinel HIV/Surveillance of2013 showed that the overall prevalence of syphilis infection as determined by RPR test in pregnant women was 3.4% (CI: 2.9%-3.9%).The mean pooled prevalence for all 16 sites was 3.35% (CI: 1.62%- 6.50%) while the median was 2.71%. The ANC clients with a positive Syphilis test had higher 41.3% (CI: 32.0-48.7) HIV prevalence compared to syphilis none reactive clients at 24.5 %(CI: 23.2-25.8).

HIV prevalence was 38.8% among syphilis infected patients and 23.9% among those testing negative for syphilis. Pregnant women attending ANC with HIV positive test were two(2) times more likely to be infected with syphilis compared to HIV negative pregnant women Odds Ratio= 2.088 (CI:1.481-2.945)(p value= 0.00003). Prevalence of Syphilis among HIV negative clients was 2.7% in 2013.

The prevalence of HIV among the STI clinic clients in 2013 was relatively high at 43.7 % (38.07-49.5%).The prevalence of syphilis infection among clients attending STI clinic was also high at 6.5% (4.2%-9.7%).

Lesotho has embarked on an accelerated programme to achieve universal accessto HIV prevention, treatment, care and support by 2010, in line with thecommitments made at the UN General Assembly high level meeting in 2006. Inresponse, the government of Lesotho has put in place several programmes anddeveloped policies to provide guidance in the areas of HIV Prevention, Care, Support and Treatment.

These guidelines are focused on HIV prevention, treatment, care and support. Specific issues regarding adults, adolescents, pregnant women and children havebeen addressed. The guidelines are linked to other HIV-related documentsincluding: HIV testing and counselling; prevention of mother-to-child transmission of HIV; home-based care; nutrition and HIV; male circumcision; TB; behavior-change communication strategy; InfectionPrevention and Control; HIV post-exposure prophylaxis; programme monitoring andevaluation; and sexually transmitted infections guidelines.

Revision of the National ART Guidelines has been necessitated by the need to haltprogression of the HIV epidemic; increase the number of people whoreceive care and treatment; and improve the quality of available care. In particular, the guidelines provide a comprehensive approach to ART and include severalimportant updates such as:

- Integration of information for the treatment of children and pregnant women
- Early initiation of adolescents and adults on treatment at a CD4<500cells/mm3
- Initiation of all HIV-infected pregnant women on lifelong therapy
- Initiation of HIV-infected children less than five years on ART
- Initiation of all HIV-TB or HIV-HBV co-infected patients on ART
- Initiation of all HIV positive partners in a discordant relationship (treatment asprevention)
- Use of viral load to monitor patients on ART
- Use of early infant diagnosis using DNA PCR tests
- Development of tools and reports to support programme monitoring and evaluationof key indicators.

CHAPTER 1: INTRODUCTION

The fourth edition of the National ART Guidelines comes following an adaptation process of the *2013 Consolidated Guidelines on the Use of Antiretroviral Drugs for Treating and Preventing HIV Infection* released by the World Health Organization. The process of developing these guidelines was led by the Ministry of Health and involved multiple stakeholders who reviewed the scientific evidence, rationale and feasibility of translating the global recommendations into country-specific national ART guidelines. Recent evidence supports ART as an HIV prevention intervention and underscores the need to continue to invest in ART for the purpose of providing both treatment and prevention of HIV transmission.

The **main highlights** for these national guidelines include the following:

- Earlier diagnosis of HIV and initiation of ART at a CD4 threshold of 500 cells/mm^3 for adults, adolescents, and older children
- ART initiation in certain populations regardless of CD4 count including pregnant and lactating women, children under 5 years, TB/HIV coinfection, HBV/HIV coinfection, and HIV infectedpartners in sero-discordant couples
- Use of simple, once daily, single pill ARV regimens across most population groups
- The use of routine viral load monitoring as the preferred approach for monitoring HIV treatment success
- Decentralization of HIV treatment and care services
- Retention across the continuum of care, service integration and linkages (ART in TB treatment settings and TB treatment in HIV care settings, ART in MNCH), human resources, laboratory and diagnostic services and procurement and supply management system

The guidelines present opportunities to reach out to more people who need treatment earlier, in line with government's commitment to universal access to HIV prevention, care and treatment services by 2015.

The **aims** of providing ART are therefore as follows:

- Maximal and durable suppression of replication of HIV
- Restoration and/or preservation of immune function
- Reduction of HIV-related morbidity and mortality
- Improvement of quality of life
- Prevention of mother-to-child transmission of HIV
- Reduction of transmission of HIV from infected to uninfected individuals through the use of ARVs by the infected individual now commonly known as 'Treatment as prevention'

The structure of these guidelines is consistent with the HIV treatment cascade from HIV diagnosis, initiation of HIV care and treatment across different population groups, patient monitoring, management of comorbidities and coinfections, nutrition, to infection control, operational and service delivery guidance, and programme monitoring and evaluation.

CHAPTER 2: HIV DIAGNOSIS

People access HIV treatment, care and prevention through the gateway of HIV testing and counselling. The overall goal of HTC is to identify as many people living with HIV as early as possible after acquiring HIV infection, and to link them appropriately and in a timely manner to prevention, care and treatment services.Efforts must be made to identify adults, adolescents and children who are likely to be HIV infected, in order to enroll them into appropriate care and treatment services. Diverse models of HIV testing and counselling are available to increase access to HIV diagnosis, including testing services in health care facilities, free standing sites and a wide range of community based approaches.

Given the risk of mother to child transmission of HIV during pregnancy and breast-feeding and the rapid progression of disease in infants, priority for early diagnosis of HIV is given to pregnant women and infants.

SECTION 2.1: HIV TESTING

The process of HIV testing and counselling should follow the minimum standards of consent, confidentiality, counselling, correct results, and linkage to care.

Consent for HIV Testing

Adults and children 12 years or above have the right to give their own informed consent for testing. For children under 12 years, a parent or caregiver who brings the child for care can give a written or verbal agreementto testing. Pre and post-test counselling must be offered to the patient or caregiver.

If the health care provider determines that the adult or child is at risk of HIV exposure or infection, consent is not required, and the provider may initiate testing with the understanding that the individual maintains the right to ‘opt out’.

Who Can Conduct Rapid Tests and Perform DBS for DNA PCR?

All those trained and accredited in HTC including:

- Laboratory Technicians and Technologists
- Counsellors (including lay counsellors under close supervision)
- Midwives
- Nurses/Nursing Assistants
- Doctors
- Pharmacists/Pharmacy Technicians
- Social Workers
- Ward Attendants
- Village/Community Health Workers (under close monitoring)
- Expert Patients (PLHIV); under close monitoring.

Provider-Initiated Testing and Counselling

HIV testing should be offered to people with unknown status at all clinical interactions with a health care facility.Provider-initiated testing and counselling is recommended to everyone (adults, adolescents and children) attending all health facilities, including medical and surgical services; sexually transmitted infection, hepatitis, TB clinics; public and private facilities; inpatient and outpatient settings; mobile or outreach medical services; services for pregnant women (antenatal care, family planning and maternal and child health settings);

services for key populations; services for infants and children; and reproductive health services.

The importance of establishing one's HIV status to one's medical management should be emphasised. Individuals should be tested unless they opt out. Those who test should receive post-test counselling whether the result is positive or negative. Individuals who opt out should be further counselled on prevention; benefits of knowing one's status; and different ways of getting tested if desired in the future.

TABLE 2.1: HIV TESTING PRIORITIES

Pregnant and Breastfeeding Women
▪ Every pregnant woman who presents at ANC should be tested. If she opts out initially, she should be encouraged to test at each follow up visit.<br>▪ During labour for women of unknown status, or immediately after delivery if testing was not possible during labour<br>▪ Testing should be repeated at 36 weeks in women who tested negative earlier in pregnancy<br>▪ Offer testing every 3 months to breastfeeding mothers who tested negative during pregnancy or delivery
**Infants and Children**
▪ All infants born to HIV-infected women.<br>▪ Children who are admitted to hospital, regardless of the diagnosis<br>▪ Children diagnosed with TB and/or malnutrition<br>▪ Orphans (with the consent of the guardian)<br>▪ Abandoned children<br>▪ Children receiving home-based care<br>▪ Children who have multiple visits to a health facility<br>▪ Children who present in Under 5 Clinic with illness<br>▪ Children of HIV positive mothers and/or fathers<br>▪ Victims of sexual abuse (in line with the Sexual Offences Act of 2003 and to facilitate provision of PEP)<br>▪ Children receiving immunizations, who have not been previously tested<br>▪ Children of any adult who is receiving chronic HIV care or is on HAART
**Adolescents**
▪ All sexually active adolescents<br>▪ All adolescents with STIs<br>▪ All adolescents accessing SRH and FP services<br>▪ Adolescents from key populations in all settings<br>▪ Adolescent victims of sexual abuse or exposure<br>▪ Any adolescent reporting injection drug use (IDU)
**Adults**
▪ Adults with unknown status or who tested uninfected > 6 months previously<br>▪ Adults who are admitted to hospital wards, regardless of the diagnosis<br>▪ Adults diagnosed with TB, malnutrition (underweight), hepatitis or STIs<br>▪ Adults receiving community home-based care<br>▪ Survivors of sexual assault<br>▪ Men presenting for circumcision services<br>▪ Adults seen as outpatients in any clinic<br>▪ Women presenting for SRH and FP services<br>▪ Any caregiver of a child receiving chronic HIV care or receiving HAART<br>▪ Health care providers

HIV testing should be offered to people with unknown status during all clinical interactions with a health facility.

Clients may seek HTC services to guide personal life decision making; plan for their future or the future of their families; understand symptoms they might be experiencing; or to support personal HIV prevention efforts.

HIV Testing and Counselling for Specific Populations

Couples

Couples counseling is a key strategy to identify people living with HIV. Offer services to married and cohabiting couples, premarital couples, polygamous unions and any other partnerships. Health providers must be aware of the potential for intimate partner-based violence and should support individuals when they do not want to test with their partners. Couples HIV testing and counselling can be offered in all settings where HIV testing and counselling is provided, including antenatal care and TB services.Offering family counselling and testing where one or both partners are living with HIV can identify children, adolescents and other household members who have not previously been diagnosed.

- Offer couples and partners HIV testing and counseling with support for mutual disclosure

Pregnant and postpartum women (lactating mothers)

Provider-initiated testing and counselling for pregnant women and linkage to prevention and care are needed to promote the mother's health and to prevent new paediatric infections.

- Provide HTC for women as a routine component of antenatal, childbirth, postpartum and paediatric care settings.
- Re-test pregnant women at 36 weeks and every 3 months during breastfeeding period for women who test negative at enrollment or during labour.

Adolescents

Adolescents are often underserved and given insufficient priority, with poor access to and uptake of HIV testing and counselling and linkage to prevention and care. Adolescents with HIV include those surviving perinatal infection and those newly acquiring infection as they become sexually active or are exposed through injecting drug use, other unsafe injections and blood transfusions. Adolescent girls and adolescents from key populations are also vulnerable to HIV infection and benefit from access to acceptable and effective HIV services, including HIV testing and counselling.

- Counsel adolescents about the potential benefits and risks of disclosure of their HIV status and empower and support them to determine if, when, how and to whom to disclose.

Key Populations

For key populations, especially those who are criminalized, HIV testing and counselling services are sometimes used in punitive and coercive ways. It is recommended that HIV testing and counselling for the most-at-risk and vulnerable groups therefore emphasize consent and confidentiality as well as ensuring that HIV testing and counselling is part of a comprehensive prevention, care and treatment programme.

TABLE 2.2: SUMMARY OF HIV TESTING AND COUNSELLING RECOMMENDATIONS

Who to Test	When to Test	Where to Test
Everyone attending<br><br>health facilities	Integrate in all health care encounters	All settings, including primary health care, outpatient medical and surgical wards, antenatal care and maternal and child health, TB, family planning and sexually transmitted infection clinics
Partners and couples	Premarital, pregnancy, after separations, new partnerships, at the start of care and ART<br><br>For the HIV-negative person in serodiscordant couples, offer re-testing every 6–12 months	Primary health care settings, voluntary counselling and testing sites, ART clinics, antenatal care, familyplanning clinics, sexually transmitted infection clinics, community and mobile outreach, home
Families of index cases	As soon as possible after the family memberis diagnosed	Primary health care settings, ART clinics, maternal and child health and antenatal care settings, homes and community and mobile outreach
Key populations: MSM, transgender people, sex workers, prisoners, factory workers	Every 6–12 months	Primary health care settings, sexually transmitted infections clinics and outreach services and other sites providing services to key populations
Pregnant women and male partners	At first antenatal care visit<br><br>Re-test in third trimester or peripartum<br><br>Offer partner testing	Antenatal care, delivery,<br><br>Postpartum
Infants and children <18 months old	Early infant diagnosis at 6 weeks for all infants whose mothers are living with HIV or if maternal HIV status is unknown; determine the final infant HIV infection status after 18 months and/or when breastfeeding ends	Maternal and child health services<br><br>Paediatric clinics<br><br>Immunization clinics
Children	Establish HIV status for all health contacts	Child inpatients and outpatients, immunization clinics
Adolescents	Integrate into all health care encounters<br><br>Annually if sexually active; with new sexual partners	Primary health care, outpatients, inpatients, voluntary counselling and testing sites, youth friendly services, family planning and sexually transmitted infections clinics

COMMUNITY-BASED HIV TESTING AND COUNSELLING

Although facility-based testing is a key approach, people living with HIV are often identified late in the course of HIV disease in clinical settings, and some populations, including men and adolescents, and especially key populations, have low utilization of health care services. Community-based testing approaches may reach people with HIV earlier in the course of HIV disease than provider-initiated testing and counselling, as well as reaching populations that may not normally attend health services.To maximize the individual and public health benefits of these recommendations, people living with HIV must be diagnosed and linked to care early in the course of HIV infection.

The use of HIV rapid tests performed by trained lay counselors and community health workers has facilitated the expansion of HIV testing and couselling in community settings including homes, transport stations, religious facilities, schools, universities, workplaces and venues frequented by key populations. Continued expansion of community-based testing is an important consideration in achieving universal knowledge of HIV status and earlier diagnosis linked to care and treatment. Community-based HIV testing and counselling includes using mobile, door-to-door, index, campaign, workplace and school-based HIV testing and counselling approaches.

Community health workers are encouraged to perform HIV testing and counseling for all community members, including infants, children and adolescents!

Community-based HIV testing and counseling with linkage to prevention, care and treatment services is recommended for family members of HIV index cases, including key populations such as migrant workers, factory workers, discordant couples and sex workers.

SECTION 2.2: HIV DIAGNOSIS IN CHILDREN

More than 90% of HIV infections in children areacquired from the mother during pregnancy, labour and delivery,or through breastfeeding. Infants and children have an immature immune system and are therefore less likely to suppress HIV viral replication once infected. Hence, HIV disease can progress much faster in infants and children than it does in adults. If untreated, approximately 40% of HIV-infected children die before their first birthday and 50% by the age of 2 years.

It is of paramount importance to diagnose HIV-exposed and HIV-infected children *early*,with rapid testing or DNA PCR test before they get sick.

HIV Testing and Counselling for Infants and young Children

The diagnosis of HIV in infants is challenging because they may carry maternal antibodies, which cross the placenta during pregnancy. Antibody (serological) tests, including HIV rapid tests, indicate HIV exposure and *possible* HIV infection. The HIV exposure of all infants attending under-5 clinic should be known in order to diagnose HIV infection as early as possible. To determine the exposure status, the mother should be tested for HIV. A positive test result for the mother indicates the infant's exposure.If the mother's status is unknown (e.g. she is unavailable or refuses to be tested), the infant should be tested with antibody tests to determine exposure status.

By the age of 9 months, majority of infants (93%) no longer have maternal antibodies, but the remaining 7% may carry maternal antibodies until 18 months of age. A virologic test such as DNA PCR is needed to definitely determine HIV status.Every infant born to an HIV infectedmother should receive a DNA PCR test to determine their HIV status at 6 and 14 weeks of age. For infants with ongoing exposure through breastfeeding, perform HIV rapid test at 9 months of age and 6 weeks after cessation of breastfeeding. ***If the infant is still breastfeeding, the child is still being exposed.***

All positive antibody tests should be followed immediately by DNA PCR test to determine the infant's infection status.All positive DNA PCR tests should be repeated immediately on a separate sample to confirm HIV infection. ART should be initiated while waiting for the repeat test results.

Exposed infants with previous negative testing need confirmatory rapid testing at 18 months. HIV-infected infants need two definitive tests to diagnose HIV infection, either 2 positive DNA PCRs or 1 positive DNA PCR and positive HIV rapid tests at 18 months.

A positive HIV rapid test in a child less than 18 months of age means that the infant has been *exposed* to HIV and may be HIV infected.

Rapid tests conducted in children less than 18 months should be done using parallel HIV testing.This means that two rapid tests such as Determine and Unigold should be done at the same time to ascertain HIV exposure status.If either or both of the tests are positive, the child is HIV-exposed.Additional testing with DNA PCR is necessary to determine infection status. It is important to ensure that infants who receive DNA PCR testing are given follow-up appointments to obtain their results and be enrolled in appropriate care, while awaiting results.

TABLE 2.3: INTERPRETATION OF DNA PCR TESTING IN CHILDREN< 18 MONTHS

DNA/PCR Result	Test Interpretation
POS	Definitively HIV infected; send a second sample for confirmation of HIV status andInitiate ART
NEG**	Definitively HIV uninfected, if outside the window period* **OR**<br>HIV exposed and possibly HIV infected, if still within the window period*

**Window period for DNA PCR test is 6 weeks.Repeat testing should be performed 6 weeks after the last exposure (6 weeks after cessation of breastfeeding).*

*** Must always consider window period when interpreting HIV negative test results*

The turn-around time for DNA PCR test results is usually 4-6 weeks.While waiting for results, an infant must be managed as an **EXPOSED** infant and started on co-trimoxazole.All infants presenting for HIV testing MUST be examined and those with signs suggestive of "presumptive diagnosis of severe HIV disease" initiated on ART.

Diagnosis of HIV in Infants between 0 and 9 Months of Age

Infants below the age of 9 months who have a known exposure to HIV can only be confirmed definitively HIV positive by two positive DNA PCR tests.If the HIV status of the mother is unknown or undocumented, a rapid test should be performed on either the mother or the infant to determine HIV exposure status. If the result of the rapid HIV test ispositive or indeterminate, then the infant has been exposed to HIV.

All HIV **EXPOSED** infants (infants born to HIV-infected mothers, or infants with positive or indeterminate rapid tests) should receive DNA PCR testing at 6 weeks of age for early infant diagnosis and should be started on co-trimoxazole prophylaxis at 4-6 weeks of age.

If **DNA PCR is positive**, then the infant is HIV infected and qualifies for ART.A second specimen should be sent for DNA PCR testing to confirm HIV infection.

If **DNA PCR is negative outside the window period**, then the infant can be declared definitively negative.

If **DNA PCR is negative within the window period** (i.e. infant is still breastfeeding or cessation of breastfeeding was less than 6 weeks prior to the test), then the baby is still HIV exposed, possibly infected and needs to be enrolled into care for an exposed infant.

Diagnosis of HIV in Children between 9 and 18 Months

At 9 months, most infants no longer possess maternal antibodies.HIV rapid testing is performed for HIV-exposed infants with ongoing exposure through breastfeeding. If the rapid test result is positive or indeterminate, then the infant is HIV exposed and possibly infected.DNA PCR testing is needed to diagnose HIV infection.

If the rapid HIV test is negative and the infant is outside the window period (six weeks after last exposure), then the infant can be considered definitively negative.Perform confirmatory rapid tests at 18 months of age.

If the test is negative and the infant is still within the window period, then the infant remains HIV exposed. Continue (or begin) exposed infant care. Rapid HIV testing should be repeated 6 weeks after complete cessation of breastfeeding.Repeat sooner if clinically indicated.

Repeat rapid testing may be done at any time if clinically indicated.

Presumptive Clinical Diagnosis of HIV in Children under 18 Months

In cases where DNA PCR is not available or results are pending, presumptive diagnosis of severe HIV disease is essential for early initiation of treatment. A child diagnosed based on the clinical criteria below may be eligible for ART even before definitive DNA PCR test results are available.

TABLE 2.4: CRITERIA FOR PRESUMPTIVE DIAGNOSIS IN CHILDREN < 18 MONTHS

A presumptive diagnosis of severe HIV disease should be made if:

- The child is confirmed HIV antibody positive;***and***
- Diagnosis of any AIDS-indicator condition(s) can be made; or
- The child is symptomatic with two or more of the following:
- Oral thrush[a];
- Severe pneumonia[b];
- Severe sepsis[c].

Other factors that support the diagnosis of severe HIV disease in an HIV seropositive child include:

Recent HIV-related maternal death; *or* advanced HIV disease in the mother;CD4< 20% in infant.

Confirmation of the diagnosis of HIV infection should be sought as soon as possible.

AIDS-defining conditions include Pneumocystis pneumonia, oesophageal candidiasis, crptococcal meningitis, cerebral toxoplasmosis, and unexplained wasting or malnutrition.

IMCI definition:

a. Oral thrush: Creamy white to yellow soft small plaques on red or normally coloured mucosa which can often be scraped off (pseudo membranous), or red patches on the tongue, palate or lining of mouth, usually painful or tender.
b. Severe pneumonia: Cough or difficulty in breathing in a child with chest in drawing, stridor or any of the IMCI general danger signs i.e., lethargic or unconsciousness, not able to drink or breastfeed; vomiting; and presence or history of convulsions during current illness.
c.Severe sepsis: Fever or low body temperature in an infant with any severe sign such as fast breathing, chest in drawing, bulging fontanelle, lethargy, reduced movement, not feeding or sucking breast milk, convulsions.

FIGURE 2.1: HIV DIAGNOSIS IN INFANTS 0-9 MONTHS OF AGE

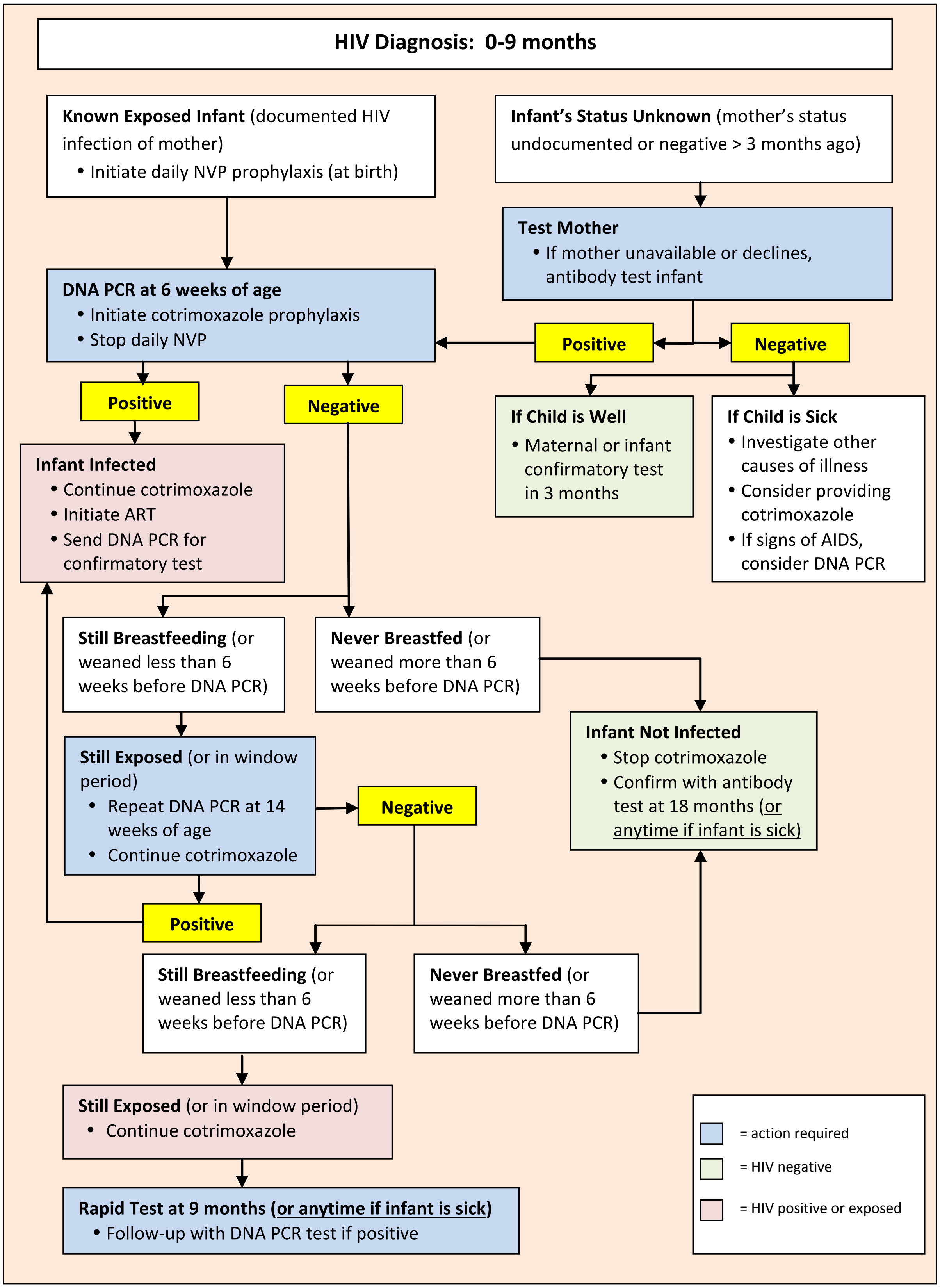

FIGURE 2.2: HIV DIAGNOSIS IN INFANTS 9-18 MONTHS OF AGE

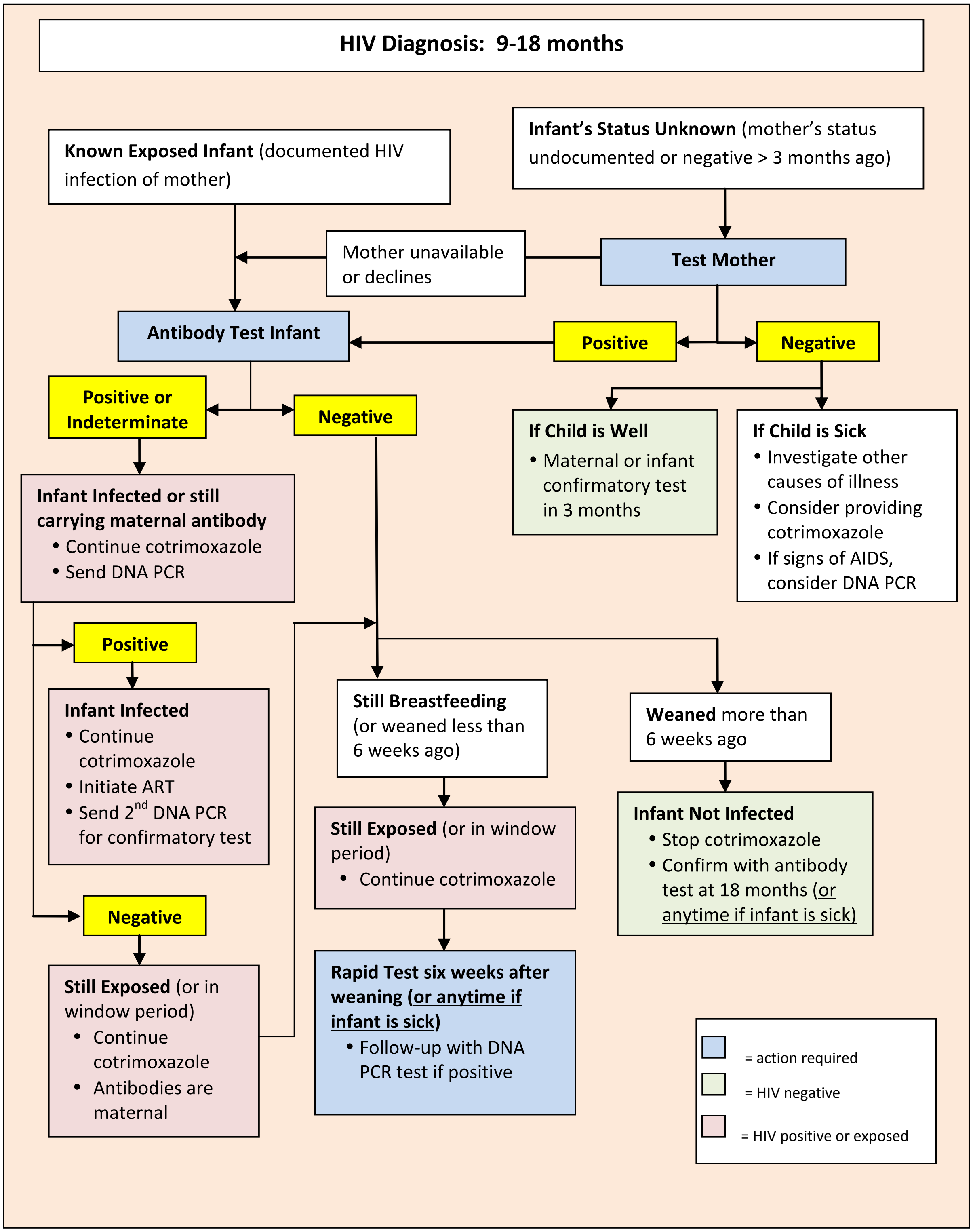

SECTION 2.3: DIAGNOSIS OF HIV IN ADULTS AND CHILDREN ≥ 18 MONTHS

By the age of 18 months, all children have lost their maternal HIV antibodies. Thus, serial rapid HIV testing can accurately confirm HIV infection.

Serial testing is done in children over the age of 18 months and in adults to determine HIV infection status.Children≥ 18 months still breastfeeding should be considered still HIV exposed.Rapid testing should be repeated 6 weeks after cessation of all breastfeeding.

Serial testing:

- If the first test is negative, the person is definitively negative.Counselling to stay negative is conducted and repeat testing is recommended for subsequent exposures or high risk behaviour.
- If the first test is positive, a different confirmation test is carried out.
- If the second test is also positive, the person is definitively positive.
- If the second test is negative, then the result is “indeterminate”.Perform repeat rapid testing in 2 weeks.If repeat testing is also indeterminate, perform ELISA testing.
- If a pregnant woman has an indeterminate test result, treat as if she is positive and enroll in appropriate care until her true status can be established.

FIGURE 2.3 ALGORITHM FOR SERIAL RAPID TESTING IN ADULTS

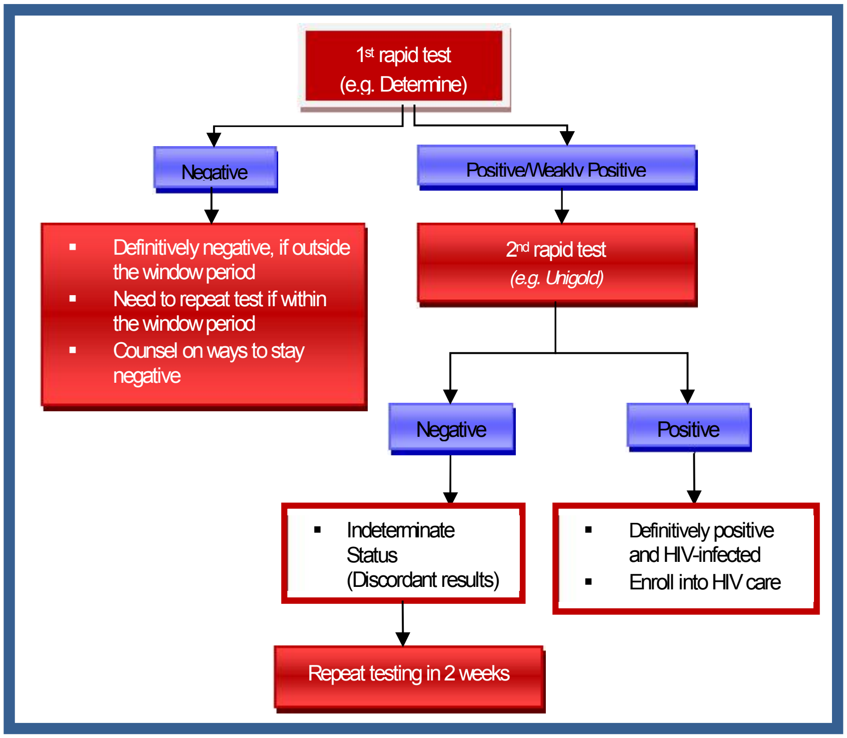

SECTION 2.4:DOCUMENTATION OF TEST RESULTS

Test results (Rapid HIV test, DNA PCR or RNA-based tests), are recorded in the hard cover of the bukana as follows.

FIGURE 2.4: RECORDING HIV STATUS IN THE BUKANA

HTC done: Y or N ***Date*: ________***   ***Type of test:***   1st rapid test: P or N   2nd rapid test: P or N   DNA PCR tests*: P or N or I   ***Where:***   P = Positive   N = Negative   I = Indeterminate   U = Unknown
**Record the date that DNA PCR test is done and the results can be filled in when they arrive.*

If available, the Under 5 stamp should be used in the Bukana and test results recorded as indicated on the stamp. Test results may also be recorded on the PMTCT stamp where available.

CHAPTER 3: HIV CAREAND TREATMENT

After a person is infected with HIV, the virus progressively weakensthe immune system.Without treatment, opportunistic infections such as TB;Pneumocystis pneumonia (PCP); thrush; malignancy; and other HIV-related conditions eventually set in.HIV-infected people need to be linked to HIV chronic care. Several good practices that can improve linkage to care include: **integrating HIV testing and counselling and care services**; **providing on-site or immediate CD4 testing with same day results**; **engaging community health workers to identify people lost to follow up**; ensure availability of **peer support**; and using new technologies such as **mobile phone text messaging**.Even after linkage to care, efforts are needed to retain patients.

SECTION 3.1:HIV CARE AND TREATMENT PACKAGE

Once diagnosed with HIV, patients should receive a comprehensive package of care.

1. Clinical evaluation
    a. Staging of clinical disease
    b. Screening for TB, sexually transmitted infectionsand pregnancy
    c. Diagnosis and management of any current OI's and co-morbid conditions.
    d. Perform anthropometric measurements (weight and height)
2. Prevention of opportunistic infections with co-trimoxazole prophylaxis and INH prophylaxis for TB.
3. Baseline laboratory investigations:
    a. CD4 count for immunologic staging
    b. Cryptococcal antigen screening for adolescents and adults with CD4 count <100 cells/mm^3
    c. VDRL or RPR to screen for Syphilis if 12 years or above
    d. HBsAgand Hepatitis C serology
    e. Haemoglobin or FBC
    f. ALT and Creatinine, urine dipstick (glucose, protein)
    g. Pregnancy test
    h. Blood glucose, cholesterol, triglycerides for PI-based regimens
4. Primary and supportive care.
5. Education, Counselling and Patient Follow up
    a. Psychosocial assessment and support, including education about HIV and adherence readiness
    b. Support for disclosure counseling
    c. Reinforce self-management including looking out for key symptoms, avoidance of dangerous habits such as alcohol consumption
    d. Need to prevent HIV transmission
    e. Develop a partnership with the patient and organize a care plan
    f. Encourage partner testing (identify discordance), encourage testing of children
    g. Link the patient to community based support and resources
    h. Record keeping for chronic care

NB. Record all the findings on the ART card in the patient's file

TABLE 3.1: PRIMARY HEALTH CARE SERVICES FOR HIV INFECTED PATIENTS

Recommended Service	Target Group
Routine immunizations	Children
Vitamin A and micronutrient supplements	Children
De-worming	Children
Monitoring of growth and development	Children and Adolescents

Annual screening - cervical smears (Pap smear) or visual inspection using acetic acid (VIAC) for cervical cancer	Sexually active women and adolescents
Nutritional evaluation and counselling	All HIV infected patients
Safe water supply	All HIV infected patients
Treatment preparedness and adherence evaluation	All HIV infected patients
Hepatitis B vaccination	Adolescents and adults with HBsAg negative result; newborns of mothers with chronic Hepatitis B
Family planning counselling and method provision	Adolescents and adults
Condom use counselling and provision	Sexually active individuals

FIGURE 3.1: ADULT AND PAEDIATRIC HIV MANAGEMENT

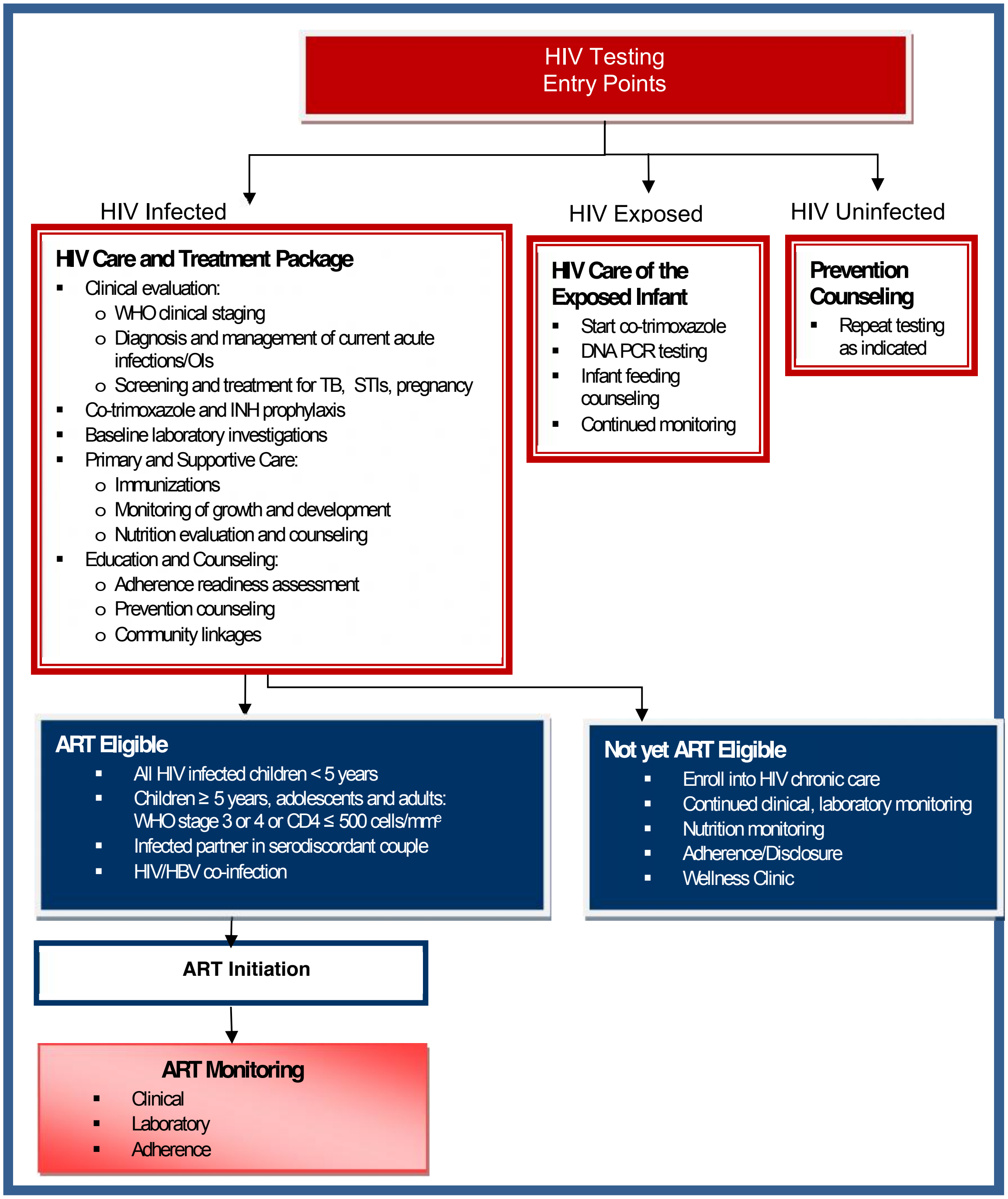

SECTION 3.2: HIV CARE AND TREATMENT PACKAGE

Clinical Evaluation of the HIV Infected Patient

A thorough clinical evaluation must be performed on all newly-diagnosed HIV-infectedpatients.A comprehensive history and physical examination allow for accurate assessment of the WHO clinical stage; screening for active TB; and diagnosis and management of any other opportunistic infections (OIs) including Hepatitis B.

1. The **history** should consist of the following components:
   a. Current symptoms
   b. Past medical history
      - birth history in children
      - growth and developmental history in children (Annexes 1,2,3)
      - obstetrics/gynaecology and STI history (in adolescent girls and women)
      - history of diabetes, hepatitis, renal insufficiency, peripheral neuropathy, hyperlipidemia, lipodystrophy
      - past hospitalization admissions
   c. Assessment of TB symptoms and history
   d. Nutritional assessment, including a feeding history and date of last breastfeeding (in children)
   e. Immunization assessment
   f. Medications, including any traditional medicines, prior ARVs, history of PMTCT, and known food or medication allergies
   g. Family history
      - HIV status of current household members
      - Possible TB contacts
   h. Social history, including initial assessment for potential barriers to adherence (familial, financial, medical and mental status) , work history, school attendance, functional ability, family planning, and alcohol / tobacco / marijuana / drug use
   i. Review of symptoms

2. **Physical examination** should proceed from head to toe, and include the following:
   a. Growth measurements
      - Weight (to be repeated at every visit)
      - Baseline length or height for all (repeated every 3 months in children)
      - Assess weight-for-height
      - Head circumference for children $\leq$ 3 years of age (repeated every 3 months until age 3 years)
      - Mid upper- arm circumference (MUAC)
   b. Vitals signs – temperature, heart rate, blood pressure
   c. General appearance (wasting, respiratory distress, pallor, jaundice, parotid enlargement, generalized oedema)
   d. Scalp (tinea, sores, signs of malnutrition)
   e. Conjunctivae (paleness, keratoconjunctivitis, jaundice)
   f. Ears (discharge)
   g. Mouth, oropharynx (thrush, ulcers, dental caries, gingivitis, Kaposi Sarcoma lesions)
   h. Lymphadenopathy (submandibular, cervical, axillary, inguinal)
   i. Lung sounds (wheeze, crackles, rhonchi); respiratory distress (nasal flaring, chest indrawing)
   j. Heart sounds (murmur, gallop, tachycardia, irregular rhythm, extra heart sounds); peripheral pulses
   k. Abdomen (hepatomegaly, splenomegaly, distension, tenderness)
   l. Genital area

- Tanner staging in older children
- Evidence of STIs (ulcers, warts, discharge)

m. Extremities
   - Fingers (paronychia, clubbing, paleness)
   - Peripheral oedema
   - Musculoskeletal (joint swelling, joint pain, back pain, muscle tenderness)

n. Skin lesions

o. Neurological (sensory abnormalities, hypotonia, hypertonia, decreased strength, developmental milestones)

Clinical Evaluation:WHO Staging

After a thorough history and physical examination have been completed, the patient should be 'clinically staged' according to the WHO criteria (see the following Tables 3.2 and 3.3).The2010WHO staging should preferably be based on the evaluation of current conditions.Past conditions can be considered for staging only if they are well documented.History of repeated chest infections, HSV and/or other OIs is indicative of an advanced clinical stage.If **documented and confirmed,** episodes of PCP, tuberculosis, cryptococcal meningitis or toxoplasmosis, clearly indicate severe HIV infection.

Note that the term 'AIDS' refers to the condition in which a person's immune system is severely compromised and serious infections have already occurred.A patient in WHO clinical stage 4 or with severe immunodeficiency is described as having AIDS.

TABLE 3.2:WHO CLASSIFICATION OF HIV-ASSOCIATED CLINICAL DISEASE

WHO clinical stage	Classification of HIV-associated clinical disease
1	Asymptomatic
2	Mild
3	Advanced
4	Severe

Clinical Evaluation:Diagnosis and Management of Opportunistic Infections and Co-morbid Conditions

Once diagnosed, treat all opportunistic infections while the patient is being prepared for ART initiation.A guide to diagnosis and management of OIs and commonly associated HIV-diseases appears in Chapter7.

Clinical Evaluation:Screening for Tuberculosis, STIs, and Pregnancy

Screening for tuberculosis, STIs, and pregnancy is an essential component of the HIV care package.HIV increases susceptibility to TB and increases the risk of progression from TB infection to active disease as well as reactivation of latent TB.

In children and adults, TB screening must be done during the initial assessment.If active TB disease is present, initiate the patient on TB treatment first then on ART within 2-4 weeks.

TABLE 3.3:WHO CLINICAL STAGING OF HIV DISEASE IN INFANTS AND CHILDREN

**CLINICAL STAGE 1**
▪ Asymptomatic<br>▪ Persistent generalized lymphadenopathy (PGL)
**CLINICAL STAGE 2**
▪ Unexplained persistent hepatosplenomegaly<br>▪ Papular pruritic eruptions<br>▪ Extensive wart virus infection (facial, >5% ofbody area or disfiguring)<br>▪ Extensive molluscum contagiosum (facial, >5%of body area or disfiguring)<br>▪ Recurrent oral ulcerations (2 or more episodes in 6 months)<br>▪ Unexplained persistent parotid enlargement<br>▪ Lineal gingival erythema (LGE)<br>▪ Herpes zoster<br>▪ Recurrent or chronic upper RTIs (otitis media, otorrhoea, sinusitis, tonsillitis)<br>▪ Fungal nail infections
**CLINICAL STAGE 3**
▪ Unexplained moderate malnutrition not adequately responding to standard therapy<br>▪ Unexplained persistent diarrhoea (14 days or more)<br>▪ Unexplained persistent fever (> 37.5°C, intermittent or constant, for longer than 1 month)<br>▪ Persistent oral candidiasis (after the first 6 weeks of life)<br>▪ Oral hairy leukoplakia<br>▪ Acute necrotizing ulcerative gingivitis/periodontitis<br>▪ Lymph node TB<br>▪ Pulmonary TB<br>▪ Severe recurrent bacterial pneumonia<br>▪ Symptomatic lymphoid interstitial pneumonitis (LIP)<br>▪ Chronic HIV-associated lung disease including bronchiectasis<br>▪ Unexplained anaemia (< 8.0 gm/dl), neutropenia (< 0.5 x $10^9/L^3$) or chronic thrombocytopenia (< 50 x $10^9/L^3$)
**CLINICAL STAGE 4**
▪ Unexplained severe wasting, stunting, or severe malnutrition not responding to standard therapy<br>▪ Pneumocystis pneumonia<br>▪ Recurrent severe bacterial infections (e.g. empyema, pyomyositis, bone or joint infection, meningitis,but excluding pneumonia)<br>▪ Chronic Herpes Simplex infection; (orolabial or cutaneous > 1 month's duration, or visceral at any site)<br>▪ Extrapulmonary TB<br>▪ Kaposi sarcoma<br>▪ Oesophageal candidiasis (or Candida of trachea, bronchi, or lungs)<br>▪ CNS toxoplasmosis (after the neonatal period)<br>▪ HIV encephalopathy<br>▪ CMV infection (retinitis or affecting another organ, with onset at age > one month<br>▪ Extrapulmonary Cryptococcosis (including meningitis)<br>▪ Disseminated endemic mycosis(extrapulmonary Histoplasmosis, Coccidiomycosis)<br>▪ Chronic Cryptosporidiosis(with diarrhoea)<br>▪ Chronic Isosporiasis<br>▪ Disseminated non-tuberculosis mycobacteria infection<br>▪ Cerebral or B cell non-Hodgkin lymphoma<br>▪ Progressive multifocal leukoencephalopathy (PML)<br>▪ HIV-associated cardiomyopathy or nephropathy<br>▪ HIV-associated rectovaginal fistula

For use in those under 15 years of age with established HIV infection

TABLE 3.4:WHO CLINICAL STAGING OF HIV DISEASE IN ADULTS AND ADOLESCENTS

CLINICAL STAGE 1
▪ Asymptomatic<br>▪ Persistent generalized lymphadenopathy
**CLINICAL STAGE 2**
▪ Moderate unexplained weight loss (under 10% of presumed or measured body weight)<br>▪ Recurrent respiratory tract infections (sinusitis, tonsillitis, otitis media, pharyngitis)<br>▪ Herpes Zoster<br>▪ Angular cheilitis<br>▪ Recurrent oral ulceration<br>▪ Papular pruritic eruptions<br>▪ Seborrhoeic dermatitis<br>▪ Fungal nail infections
**CLINICAL STAGE 3**
▪ Unexplained severe weight loss (over 10% of presumed or measured body weight)<br>▪ Unexplained chronic diarrhoea for longer than 1 month<br>▪ Unexplained persistent fever (intermittent or constant for longer than 1 month)<br>▪ Persistent oral candidiasis<br>▪ Oral hairy leukoplakia<br>▪ Pulmonary tuberculosis (TB)<br>▪ Severe bacterial infections (e.g. pneumonia, empyema, pyomyositis, bone or joint infection, meningitis, bacteraemia)<br>▪ Acute necrotizing ulcerative stomatitis, gingivitis or periodontitis<br>▪ Unexplained anaemia (< 8 g/dl), neutropenia (< 0.5 x 10^9/l) and/or chronic thrombocytopenia (< 50 x 10^9/l)
**CLINICAL STAGE 4**
▪ HIV wasting syndrome<br>▪ Pneumocystis pneumonia<br>▪ Recurrent severe bacterial pneumonia<br>▪ Chronic herpes simplex infection (orolabial, genital or anorectal of more than one month's duration, or visceral at any site)<br>▪ Oesophageal candidiasis (or candidiasis of trachea, bronchi or lungs)<br>▪ Extrapulmonary TB<br>▪ Kaposi sarcoma<br>▪ Cytomegalovirus infection (retinitis or infection of other organs)<br>▪ Central nervous system (CNS) toxoplasmosis / HIV encephalopathy<br>▪ Extrapulmonary cryptococcosis including meningitis<br>▪ Disseminated non-tuberculous mycobacteria infection<br>▪ Progressive multifocal leukoencephalopathy (PML)<br>▪ Chronic cryptosporidiosis<br>▪ Chronic isosporiasis<br>▪ Disseminated mycosis (extrapulmonary histoplasmosis, coccidiomycosis)<br>▪ Recurrent septicaemia (including non-typhoidal *Salmonella*)<br>▪ Lymphoma (cerebral or B cell non-Hodgkin)<br>▪ Invasive cervical carcinoma<br>▪ Atypical disseminated leishmaniasis<br>▪ Symptomatic HIV-associated nephropathy or HIV-associated cardiomyopathy

Screening for Active Tuberculosis

Active TB is common in HIV-infected patients.Prior to initiation of ART, it is very important to rule out active TB.A thorough assessment includes history, physical examination and appropriate investigations.

Signs and symptoms of possible TB infection include:

- History of TB contact
- Cough
- Fever
- Night sweats
- Weight loss
- Lymphadenopathy

Possible investigations include:

- Sputum for acid-fast bacilli (AFB)
- Chest x-ray (CXR)
- Rapid molecular assays – GeneXpert, Line Probe Assay (LPA)
- Culture and drug-susceptibility testing (DST) of sputum, blood, other specimens
- If EPTB is suspected, further investigations can be carried out, including: fine needle aspirates of enlarged lymph node, lumbar puncture, thoracentesis, paracentesis, abdominal ultrasound, spinal x-ray, etc.Specific investigations will depend upon the site of disease.**See National TB Guidelines for details.**

In **children**, TB disease is difficult to confirm.There should be a low threshold to start TB treatment.If possible, sputum or gastric aspirates for GeneXpert should be obtained from all infants, children and adolescents with presumptive TB.

TABLE 3.5: DIAGNOSING TB IN CHILDREN

Clinical Suspicion	CXR Findings	Management
**High**<br>▪ TB contact<br>▪ Clinical symptoms	Suspicious	Initiate TB treatment
	Not suspicious	Initiate TB treatment
**Moderate**<br>▪ Clinical symptoms	Suspicious	Initiate TB treatment
	Not suspicious	Monitor and consider initiating TB treatment if no response to antibiotics; consider other diagnoses
**Low**<br>▪ Intermittent clinical symptoms	Suspicious	Monitor and consider initiating TB treatment if no response to antibiotics; consider other diagnoses
	Not suspicious	No TB treatment needed

Patients diagnosed with active TB should be initiated on ATT and then ART 2-4 weeks thereafter.

Screening for STIs/Pregnancy

Treatment for commonly associated STIs, such as syphilis, gonorrhoea, Chlamydia, Herpes simplex, and genital warts is important.Details can be found in National Guidelines for STI Management.

Lastly, a pregnancy test is crucial for early identification of pregnant women.This assists in determining timing of treatment.

Prevention of new Infections with Co-trimoxazole Prophylaxis

Co-trimoxazole is an inexpensive and cost-effective way to reduce morbidity and mortality in HIV-infected patients.Daily co-trimoxazole protects against:

- *Pneumocystis* pneumonia (PCP)
- Toxoplasmosis
- Diarrhoea caused by *Isospora belli* and *Cyclospora* species
- Bacterial infections, including bacterial pneumonia and urinary tract infections

Co-trimoxazole prophylaxis is recommended for **children** under the following circumstances:

- All HIV-exposed children; starting at 4-6 weeks of age.It should be continued until:
    - HIV infection has definitively been excluded in the child *and*
    - The infant is no longer at risk of acquiring HIV through breastfeeding
- All children < 5 years confirmed to be HIV-infected
- For children 5 years or above, follow adult and adolescent guidelines

Co-trimoxazole prophylaxis is recommended for **adults and adolescents** under the following circumstances:

- All those in clinical stages3 and 4
- Those in clinical stage 1 and 2 where the CD4 count is≤ 350 cells/mm^3.
- All those in clinical stages 2, 3 and 4 where CD4 is not available

Note that this implies that all those receiving TB treatment should simultaneously receive daily co-trimoxazole.

TABLE 3.6: CO-TRIMOXAZOLE PROPHYLAXIS IN ADULTS AND ADOLESCENTS

WHO Clinical Staging	CD4 is available	CD4 is not available
4	Daily Co-trimoxazole (CTX)	
3	Daily CTX	
2	Daily CTX if CD4 < 350	Daily CTX
1	Daily CTX if CD4 < 350	Do notgive CTX routinely

Dosing of Co-trimoxazole

TABLE 3.7:DOSE OF CTX FOR PREVENTION OF PCP

Age	Suspension (200/40mg per 5 ml)	Single Strength adult tablet(400/80mg)	Double Strength adult tablet(800/160mg)
< 6 Months	2.5ml	¼ tablet	–
6 mo- 5 Years	5 ml	½ tablet	–
6-14 Years	-	1 tablet	½ tablet
>14 Years	–	2 tablets	1 tablet

Co-trimoxazole should be avoided in the following situations:

- History of a severe rash with prior use of CTX (or other 'sulfa' drug)
- Pre-existing severe renal disease
- Pre-existing severe hepatic disease

Note that those patients who are unable to take co-trimoxazole should be offered Dapsone 100 mg daily (children: 2mg/kg daily) to help prevent *Pneumocystis* pneumonia (PCP) and as an alternative to co-trimoxazole in all situations.

When to Discontinue Co-trimoxazole Prophylaxis

Co-trimoxazole prophylaxis should only be discontinued in adults and children $\geq$ 5 years on ART; following two consecutive CD4 counts of > 350 cells/mm^3.Since CD4 counts are to be monitored routinely every 6 months in those on ART, this implies that the CD4 count will have been > 350 cells/mm^3 for at least 6 months.CTX may be safely discontinued in those with previous PCP infection if ALL conditions above are met.This decision should be made by a physician with experience in treating HIV-infected persons.

SECTION 3.3: BASELINE LABORATORY INVESTIGATIONS

Laboratory investigations enhance clinical evaluation.Clinical assessment is the primary tool for evaluating patients both before initiation and after ART treatment has been initiated.Laboratory investigations can help inform when to start ART and which regimen to choose but are not essential for ART initiation.

Inability to perform laboratory investigations (including CD4 count/%) should not prevent patients who are eligible from being initiated on ART.

CD4:Immunological Staging

- CD4 counts, measured in cells/mm^3, give an approximate measure of the strength of one's immune system.The CD4 count result can predict the risk and type of subsequent opportunistic infections (OIs).The count declines with disease progression.
- Note that CD4 parameters in children differ from adults since absolute CD4 count varies with age.In children without HIV, absolute CD4 counts are comparatively higher than in adults.Normal absolute CD4 counts in children slowly decline and reach adult levels by the age of 5 years.CD4 percentage is less variable than absolute count.
- CD4 percentage is the preferred immunological parameter for monitoring disease progression in children less than 5 years.
- CD4 count/percentage can decline very rapidly in infants < 12 months of age.
- Due to their immature immune system, HIV-infected infants < 12 months may suffer from opportunistic infections regardless of their CD4 count/percentage.Close clinical monitoring is therefore imperative in younger infants.
- Conditions such as tuberculosis and severe malnutrition can also cause low CD4 count/percentage; resulting in severe immunosuppression.

TABLE 3.8: WHO CLASSIFICATION OF HIV IMMUNODEFICIENCY IN CHILDREN

Classification of HIV-Associated Immunodeficiency		< 11 Months (%)	12-35 Months (%)	36-59 Months (%)	≥ 5 Years (cells/mm^3)
Not significant		> 35%	> 30%	> 25%	> 500
Mild		30-35%	25-30%	20-25%	350-499
Advanced		25-30%	20-25%	15-20%	200-349
Severe	CD4%, or absoluteCD4 count	< 25%, or < 1500 cells/mm^3	< 20%, or < 750 cells/mm^3	< 15%, or < 350 cells/mm^3	< 15%, or < 200 cells/mm^3

Source:WHO Antiretroviral Therapy of HIV Infection in Infants and Children:Towards Universal Access

If there is a discrepancy between CD4% and CD4 absolute count, then CD4% should be used for children < 5 years and the absolute count should be used for children aged 5 years or above.If no CD4% is available, then severe immunosuppression should be categorized using CD4 count as noted above.CD4 percentage can also be calculated using the following formula:

$$CD4\% = \frac{\text{CD4 absolute count} \times 100}{\text{Total Lymphocyte Count (TLC)}}$$

TLC = White Blood Count (WBC) x % Lymphocytes

Example:

10 month old with absolute CD4 count of 1,000; WBC of 8,000; and lymphocytes of 50%

TLC= 8,000 X 0.50= 4,000

$$CD4\% = \frac{1{,}000 \times 100}{4{,}000} = 25\%$$

This infant falls into the severe immunodeficiency category by both CD4% and absolute CD4 count.

TABLE 3.9: CLASSIFICATION OF IMMUNODEFICIENCY IN ADOLESCENTS AND ADULTS

Immunological Category	CD4 Count
No significant immunodeficiency	> 500 cells/mm^3
Evidence of mild immunodeficiency	350-499 cells/mm^3
Evidence of advanced immunodeficiency	200-349 cells/mm^3
Evidence of severe immunodeficiency	< 200 cells/mm^3

SECTION 3.4:PRIMARY AND SUPPORTIVE CARE

Immunizations

Immunizations in an HIV-infected child should follow the standard Lesotho immunization schedule:

TABLE 3.10:IMMUNIZATION SCHEDULE

Age	Immunizations
Birth	BCG
0-2 weeks	OPV
6 weeks	Pentavalent vaccine (DPT, Hep B, HiB); OPV
10 weeks	Pentavalent vaccine (DPT, Hep B, HiB); OPV
14 weeks	Pentavalent vaccine (DPT, Hep B, HiB); OPV
9 months	Measles
18 months	Measles, DT

- Children who have or are suspected to have HIV infection but are not yet symptomatic should be given all appropriate vaccines, including BCG, measles, and yellow fever vaccine (if indicated).

- BCG and yellow fever vaccines should not be given to a child who has symptomatic HIV infection or who is severely immunodeficient.Virtually all HIV-exposed and HIV-infected infants are asymptomatic at birth and can receive the BCG vaccine at birth.
- Consider giving measles vaccine early (at 6 months and again at 9 and 18 months) in children with HIV infection.Measles vaccine may be given to a child with symptomatic HIV infection as long as there are no features suggestive of severe immunodeficiency.

Routine Oral Vitamin A Supplementation

The following dosages should be administered to children **every 6 months**:

Age	Dose of Vitamin A
6 months	100,000 Units
12 months to 5 years	200,000 Units

Treatment for Worms

Children should be de-wormed routinely every 6 months until the age of 12 years.

TABLE 3.11: DE-WORMING TREATMENT

Age	Weight	Albendazole		Mebendazole (alternative)
12-24 months	<10kg	200 mg once	or	100 mg BD for 3 daysor 500 mg stat
25–60 months<br>> 5 yrs	>10kg	400 mg once		500 mg once

Cervical Cancer Prevention

Cervical cancer is the most common cancer among females in Lesotho, with a peak incidence at age of 40-49 years. HIV positive women are at a higher risk of pre-cancer and invasive cervical cancers.Routine cervical PAP smears or Visual Inspection using Acetic Acid (VIAC) should be performed to screen for cervical cancer in women.**All HIV infected women of reproductive age should have cervical cancer screening (Pap smear or VIAC) at baseline and thereafter annually**. Adolescent girls living with HIV need screening annually once they are sexually active.Patients with abnormal test results should be referred to the appropriate health facility (hospitals) for immediate management of pre-cancerous and cancerous lesions.

Nutrition

The nutritional status of HIV infected patients should be addressed as part of the comprehensive care package.Children in particular, should be screened for malnutrition.Refer to Chapter 8 and the National Guidelines for the Integrated Management of Acute Malnutrition for details regarding nutritional assessment and management.

Education and Counselling

In preparation for ARV initiation, begin HIV education and counselling and performa psychosocial assessment.This will assist with future adherence counselling and identification of potential barriers to successful treatment and adherence as well as potential strategies to optimise care and treatment.

Counselling should reinforce the importance of preventing HIV transmission and preparation for ARV treatment.

Discussing available community linkages and support networks with the patient or caregiver is beneficial for the future care of the patient.

Community support can assist with patient adherence, disclosure, stigma mitigation and defaulter tracking.

SECTION 3.5: PREVENTING TRANSMISSIONOF HIV AMONG PLHIV

From a public health perspective, PLHIV constitute the most important group in terms of HIV prevention.A change in the risk behaviour of an HIV infected person has a greater impact on the transmission of HIV.Enrollment into care facilitates identification of PLHIV with behavioural risk factors and interventions to reduce such factors.It also facilitates the identification of clinical risk factors such as STIs and their treatment; interventions to reduce unplanned pregnancies; and prevention of mother-to-child transmission of HIV.

Pre-ART care includes referral to and from support systems such as Blue Cross and Alcoholic Anonymous Clinics. This has the potential to normalize unstable lifestyles by limiting excessive alcohol use in preparation for ART initiation and facilitates risk reduction as well.Positive prevention strategies at group and individual levels lead to reduction in HIV risk behaviour among PLWHIV. The strategies include support to improve consistent condom use; reduction of unprotected sex; treatment of intercurrent sexually transmitted infections and reduction in the number of sexual partners.

SECTION 3.6:CARE OF THE HIV-EXPOSED INFANT

Most infants with HIV infection are asymptomatic at birth. Without identification and treatment close to half of HIV infected infants will die before their second birthday.It is therefore, essential that infants exposed to HIV are placed on appropriate prophylaxis and monitored closely until their HIV status is confirmed. The table below summarizes the monitoring schedule for the HIV-exposed infant.

All efforts should be made to ensure that HIV-exposed infants are not lost to follow-up.HIV-exposed infants should be followed until their status is definitively known.If they are definitively negative, they should continue attending under- five clinic for primary health care services.If they are definitively positive they should be initiated on ART.

TABLE 3.12: SCHEDULE FOR MONITORING VISITS FOR HIV EXPOSED INFANTS

Age	Services to be offered
**At birth**	Clinical evaluation (thorough history and physical examination)<br>Weight<br>Infant feeding counselling and support<br>Ensure that infant ARV prophylaxis(daily NVP) is being provided
**7 days**	Clinical evaluation (thorough history and physical exam)<br>Infant feeding counselling and support<br>Ensure that infant ARV prophylaxis(daily NVP) is being provided

**6 weeks**	Clinical monitoring (thorough history and physical exam)<br>Infant feeding counselling and support<br>Provide Immunizations<br>Monitor growth and development<br>Send DBS for DNA PCR<br>Initiate CTX prophylaxis<br>Stop Infant NVP prophylaxis
**10 weeks**	Clinical monitoring (thorough history and physical exam)<br>Infant feeding counselling and support<br>Provide immunizations<br>Provide DNA PCR results to caregiver; If DNA PCR positive; initiate ART and send confirmatory DNA PCR<br>Monitor growth and development<br>Continue CTX prophylaxis
**14 weeks**	Clinical monitoring (thorough history and physical exam)<br>Infant feeding counselling and support<br>Provide Immunizations<br>Provide DNA PCR results to caregiver if not given; If DNA PCR positive:initiate ART and send confirmatory DNA PCR;<br>Repeat DNA PCR test<br>Monitor growth and development<br>Continue CTX prophylaxis
**Monthly visits until 12 months of age**	Clinical monitoring (thorough history and physical exam)<br>Infant feeding counselling and support<br>Provide Immunizations, Vit A, routine de- worming as per MOH guidelines<br>Provide DNA PCR results to caregiver if not given; If DNA PCR positive; initiate ART and send confirmatory DNA PCR<br>Monitor growth and development<br>Continue CTX prophylaxis

Clinical evaluation

- Growth and Development
  - Measure weight, height, and head circumference
  - Assess developmental milestones
- History and Physical
  - Assess for opportunistic infections and signs and symptoms suggestive of HIV infection.Treat as indicated.
  - Remember that HIV-infected infants will present with common infections – pneumonia, diarrhoea, otits media, etc.
- Initiation and continuation of co-trimoxazole prophylaxis (initiation at 4-6 weeks) or the earliest possible time an infant is in contact with a health facility.
- Commence NVP prophylaxis at birth and continue until the infant is 6 weeks old.
- Nutritional assessment,dietary advice and management of malnutrition as appropriate
  - Review infant feeding practices (see Chapter 8 and Infant and Young Child Feeding National Guidelines)
- Primary and supportive care:Immunizations, Vitamin A, and de-worming for children
- Re-assess need for repeat testing (DNA PCR) depending on age and exposure history

Identifying Infants with Signs and Symptoms of HIV Infection

Infants may be HIV-infected but completely asymptomatic.More commonly, they present with recurrent common infections.All HIV-exposed infants may be HIV-infected and must be

started on co-trimoxazole prophylaxis and followed closely as outlined above.DNA PCR testing should be performed to determine definitive diagnosis.Use criteria for presumptive diagnosis of severe HIV disease to initiate ART in sick infants awaiting DNA PCR testing or results.

Co-trimoxazole Prophylaxis

Co-trimoxazole can only be discontinued once HIV is definitively excluded. An HIV-exposed infant or child can be declared definitively HIV-negative if he or she fulfils the following requirements:

- Negative rapid test or negative DNA PCR and outside of window period (6 weeks after complete cessation of breastfeeding).

SECTION 3.7: SELECTING PATIENTS FOR ANTIRETROVIRAL THERAPY

Eligibility for ART in infants and children

- Initiate ART in all children under 5 years, irrespective of the CD4 count or clinical stage.
- Initiate ART in children aged > 5 years, WHO clinical stage 3 or 4, irrespective of CD4 count or percentage.
- Initiate ART in children aged > 5 years with CD4 <500 cells/mm^3.
- Initiate ART in an infant or child (under 18 months) who meets the presumptive criteria for severe HIV disease.

TABLE 3.13: RECOMMENDATIONS ON WHEN TO START ART IN CHILDREN

Age	When to start
Children under 5years	Treat all
5 years and above	WHO stage 3 or 4 or<br>CD4 ≤500 cells/mm^3

Issues to consider in initiating ART in children

*Psychosocial factors:*It is important to identify and counsel at least one dedicated caregiver who can supervise and/or give medicines. Disclosure to another adult in the same household (secondary caregiver) is encouraged to assist with medication.

Disclosure: The process of disclosure to the child should be initiated as early as possible, usually from as early as 5 – 7 years of age. *Adherence is improved in children who know their status and are supported to adhere to medicines.*

Eligibility for ART in adolescents and adults

- Initiate ART in adolescents and adults with a CD4 count of ≤ 500 cells/mm^3.
- Initiate ART in all pregnant and breast-feeding women living with HIV
- Initiate ART in individuals with HIV in sero-discordant relationships.
- Initiate ART in all individuals with HBV co-infection.

TABLE 3.14: ELIGIBILITY CRITERIA FOR ART INITIATION FOR ADULTS AND ADOLESCENTS

Criteria	Treatment Decision
WHO clinical stage 3 or 4	Treat all regardless of CD4 cell count
WHO clinical stage 1 or 2	Treat CD4 ≤ 500 cells/mm³
HIV sero-discordant couples	Treat infected partner regardless of CD4 cell count
TB co-infection	Treat all HIV-infectedTB patients regardless of CD4 cell count
Hepatitis B co-infection	Treat regardless of CD4 count
HIV-infectedPregnantand lactating women	Treat all regardless of CD4 cell count

If a CD4 count is not available, this should not delay the initiation of ART in an adult or child who is WHO Clinical Stage 3 or 4.All children less than 5 years of age, pregnant and breastfeeding women diagnosed must be initiated on ART irrespective of their CD4 count.

Other criteria to assess readiness to start ART

ART requires a life-long commitment and patient's willingness to adhere to treatment for life is paramount. All patients should receive 1-3 counselling sessions in order to learn about the following aspects of ART:

- HIV, its life-cycle, and how it affects a person's immune system
- How HIV is transmitted
- The difference between HIV and AIDS
- The value of CD4 and viral load testing
- How antiretroviral medication works
- Possible side effects of ARVs
- Consequences of non-adherence ('resistance')

Other criteria that can help assess a patient's readiness to begin ART include:

- The involvement of a Treatment Supporter (also known as a 'treatment buddy' or 'treatment assistant':
    - This is usually a friend or family member who can help with adherence, and implies that the patient has disclosed her/his status to at least one person.The participation of a treatment supporter is strongly encouraged, but not mandatory.If a patient has difficulty in identifying a treatment supporter, the health facility should help to identify a suitable person, often through a support group or community health worker.It is desirable to have the treatment supporter living within walking distance to the patient's home and be older than 12 years of age.For children, a reliable, consistent caregiver should be identified to give medicines to the child and bring the child for clinic visits.If possible a second caregivershould also be identified to assist with medications when the primary caregiver is unavailable
- Determination to attend a clinic visits:
    - It is recommended that patients receive ART from the nearest clinic to their home.However, this is not obligatory, since there are valid reasons that may force patients to seek ART services elsewhere.Once such patients are stable on ART, and if all parties agree, the person can be transferred to the nearest clinic using a standardized Transfer Letter (Annex 7).
- Psychosocial issues**:**
    - Ideally, the home environment should be supportive of patients receiving ART. Factors that should be considered include a patient's or caregiver's mental health status, alcohol or drug abuse, or the presence of domestic violence and financial status.

Refer to Chapter 6 for details regarding adherence counselling and readiness assessment.

SECTION 3.8: ADULTS AND CHILDREN NOT YET ELIGIBLE FOR ART

All children, adolescents and adults living with HIV need chronic care. Enroll patients into care at diagnosis.The complete package of care outlined in the beginning of this chapter supports optimal health outcomes for those not yet eligible for ART. Regular clinical review and laboratory monitoring will enable timely initiation on ART as eligibility changes.Encourage community support throughout the continuum of care.

Children who are not yet eligible for ART on clinical or immunological grounds must be assessed regularly.HIV disease can progress rapidly, even in those with seemingly good CD4 counts.Hence, close clinical monitoring in HIV-infected children is of utmost importance.Clinical evaluation is needed every three months with CD4 monitoring every 6 months.Children should continue to access the comprehensive package of care.

Adults and adolescents in the early stages of HIV infection (clinical stages 1 and 2) with CD4 counts >500 cells/mm^3 do not need ART.They instead need the comprehensive package of medical care outlined at the beginning of this section.Clinical evaluation and CD4 testing is needed every 6 months.It can be done more frequently if clinically indicated

An important aspect of care for this group of patients is a healthy and positive lifestyle, which is outlined in Chapter 9 - Wellness Information. Often, regular support group sessions every three months are suitable for addressing lifestyle issues.

CHAPTER4: ANTIRETROVIRAL TREATMENT REGIMENS

SECTION 4.1: FIRST LINE REGIMENS

For treatment purposes, three anti-retroviral (ARV) drugs are given together.In a treatment-naïve patient (one who has never used ARVs in the past), the first-line regimen should consist of two Nucleoside Reverse Transcriptase Inhibitors (NRTIs) plus one Non-nucleoside Reverse Transcriptase Inhibitor (NNRTI).For children younger than three years, a PI-based regimen is the preferred approach.

If fewer than 3 ARVs are used for treatment, resistance of HIV to individual ARVs will eventually develop, resulting in treatment failure.

The goals of anti-retroviral therapy (ART) include:

- Reduction in HIV-related morbidity and mortality
- Improvement in quality of life and prolonged survival
- Restoration and preservation of immune function
- Maximal and durable suppression of HIV replication
- Accelerated growth (for children)

TABLE 4.1: SUMMARY OF FIRST-LINE ART REGIMENS

First-line ART	Preferred first-lineregimens	Alternative first-line regimens
**Adults**<br>including pregnant and breastfeeding women and adults with TB and HBV coinfection	**TDF + 3TC + EFV**	AZT + 3TC + EFV<br>AZT + 3TC + NVP<br>TDF + 3TC + NVP<br>*ABC+3TC+EFV (or NVP)
**Adolescents**<br>(10 to 19 years)<br>≥35 kg		AZT+3TC+EFV<br>AZT+3TC+NVP<br>TDF+3TC +NVP<br>*ABC+3TC+EFV (or NVP)
**Children**<br>(3 to 9years)<br>**Adolescents**<br><35 kg	**ABC + 3TC + EFV**	AZT+ 3TC + EFV<br>ABC+3TC+ NVP<br>AZT+3TC+ NVP
**Children**<br><3 years	**ABC+ 3TC + LPV/r**	AZT + 3TC + LPV/r<br>ABC + 3TC + NVP<br>AZT + 3TC + NVP

**ABC or boosted PIs (ATV/r, LPV/r) can be used in special circumstances. For all patients on Emtricitabine (FTC), Lamivudine can be given as both drugs have the pharmacological properties.*

Special Considerations for ART in Children

New evidence has become available for young children suggesting the superiority of a LPV/r-based regimen regardless of PMTCT exposure.

- Use LPV/r-based regimen as first-line ART for all children infected with HIV younger than three years (36 months) of age, regardless of NNRTI exposure. If LPV/r is not feasible, treatment should be initiated with a NVP-based regimen.
- For infants and children younger than three years and co-infected with HIV/TB; ABC + 3TC + NVP is recommended for children during TB treatment.Once TB treatment is completed, place child on recommended ABC + 3TC + LPV/r.

- For HIV-infected children three years and older (including adolescents), EFV is the preferred NNRTI for first-line treatment and NVP is the alternative.
- For HIV-infected children three to nine years old (or adolescents less than 35kg), the NRTI backbone for an ART regimen should be one of the following, in preferential order:
  - **ABC + 3TC**
  - **AZT + 3TC**
- For adolescents infected with HIV (10 to 19 years old) weighing 35kg or more, the NRTI backbone for an ART regimen should align with that of adults and be one of the following in preferential order:
  - **TDF + 3TC**
  - **ABC + 3TC**
  - **AZT + 3TC**

TB co-treatment in children with HIV

TB is one of the most common opportunistic infections affecting children with HIV. Selecting regimens that are compatible with TB therapy is therefore essential. Interactions between rifampicin and LPV/r or NVP mean that co-treatment in children under three years is challenging.

TABLE 4.2: RECOMMENDED ART REGIMENS FOR CHILDREN WHO NEED TB TREATMENT

.

Recommended regimen for children and infants initiating TB Treatment while receiving ART		
Child on standard PI-based regimen (two NRTIs +LPV/r)	Younger than3 years	Substitute NVP for LPV/r, ensuring that dose is 200mg/m^2<br>*or*<br>Triple NRTI (AZT + 3TC + ABC)
	3 years and older	If the child has no history of failure of an NNRTI-based regimen:<br>Substitute with EFV or<br>Triple NRTI (AZT + 3TC + ABC)<br><br>If the child has a history of failure of an NNRTI-based regimen:<br>Triple NRTI (AZT + 3TC + ABC)<br>Consultation with experts for constructing a second- line regimen

Dosing of ARVs in children is based on weight. Refer to Annex 4 for dosingrecommendations.

First-line ART for adults and adolescents

- First-line ART should consist of two nucleoside reverse-transcriptase inhibitors (NRTIs) plus a non-nucleoside reverse-transcriptase inhibitor (NNRTI)
- **TDF + 3TC + EFV** as a fixed-dose combination is recommended as the preferred option to initiate ART
- If TDF + 3TC + EFV is contraindicated or not available, one of the following is recommended
  - AZT + 3TC + EFV
  - AZT + 3TC + NVP
  - TDF + 3TC + NVP

First-line ART for pregnant and breastfeeding women and ARV drugs for their infants

- A once-daily fixed-dose combination of TDF + 3TC + EFV is recommended as first-line ART in pregnant and breastfeeding women, including pregnant women in the first trimester of pregnancy and women of childbearing age. The recommendation applies for lifelong treatment initiated for PMTCT.
- HIV-exposed infants should receive six weeks of infant prophylaxis with daily NVP. Infant prophylaxis should begin at birth or when HIV exposure is recognized postpartum.

HIV infected pregnant women require the same care for their own health as any other infected adults (See Chapter 3 on HIV Care and Treatment).In addition, they need special education and counselling about PMTCT, family planning, follow-up of exposed infant(s), partner involvement, family care and infant feeding options. Eligible pregnant women should be 'fast-tracked' for initiation of ART.

TABLE 4.3: SUMMARY OF MATERNAL AND INFANT ARV PROPHYLAXIS FOR DIFFERENT CLINICAL SCENARIOS

Scenario	Maternal ARV prophylaxis[a]	Infant ARV prophylaxis	Duration of infant ARV prophylaxis
Mother diagnosed with HIV during pregnancy	Initiate maternal ART	NVP	6 weeks
Mother diagnosed with HIV during labour or immediately postpartum and plans to breastfeed	Initiate maternal ART	NVP	6 weeks
Mother diagnosed with HIV during labouror immediately postpartum and plans replacement feeding	Refer mother for HIV care and evaluation for treatment	NVP	6 weeks
Infant identified as HIV exposed after birth (through infant or maternal HIV antibody testing) and is breastfeeding	Initiate maternal ART	NVP	Until 6 weeks of age
Infant identified as HIV exposed after birth (through infant or maternal HIV antibody testing)and is not breastfeeding	Refer mother for HIV care and evaluation for treatment	NVP	Until 6 weeks of age
Mother receiving ART but interrupts ART regimen while breastfeeding (such as toxicity, stock-outs or refusal to continue)	Determine an alternative ART regime; counsel regarding continuing ART without interruption	NVP	Until 6weeks after maternal ART is restarted or until 1 week after breastfeeding has ended

[a]*Ideally, obtain the mother's CD4 cell count at the time of initiating or soon after initiating ART.*

TABLE 4.4: INFANT DOSING OF NEVIRAPINE SYRUP

Term Infants		Pre-term Infants	
NVP Daily DoseBirth to 6 wks		NVP Daily Dose Birth to 2wks	
2 - 2.49 kg	1 ml	1 – 1.8 kg	0.3 ml
≥ 2.5 kg	1.5 ml	1.8 - 2 kg	0.5 ml
		NVP Daily Dose 2wks to 6 wks	
		≥ 2 to 6 wks	1 ml

SECTION 4.2 SECOND-LINE REGIMENS

Patients who fail 1st-line treatment, are switched to second-line ART. The criteria for switching patients are elaborated in chapter 5. The second-line regimen will still consist of two NRTIs but with the addition of a PI. The table below summarizes the preferred second line ART regimens.

TABLE 4.5: SUMMARY OF PREFERRED SECOND-LINE ART REGIMENS

Second-line ART			Preferred regimens	Alternative regimens
**Adults and adolescents (≥10 years), including pregnant and breastfeeding women**			AZT+ 3TC+ LPV/r<br>AZT +3TC+ATV/r	TDF+3TC+ ATV/r<br>TDF +3TC+LPV/r
**Children**	If a NNRTI-based first line regimen was used		AZT +3TC+ LPV/r	ABC +3TC +LPV/r<br>TDF +3TC+LPV/r
	If a PI-based first line regimen was used	<3 years	No change from first line regimen	AZT +3TC +NVP<br>ABC +3TC + NVP
		3 years to less than 10 years	AZT +3TC+ EFV	AZT +3TC+ NVP

If on TDF or ABC in first-line, second-line should be AZT and vice versa. For all patients on Emtricitabine (FTC), Lamivudine can be given as both drugs have the pharmacological properties.

Second-line ART for adults and adolescents

- Second-line ART for adults should consist of two nucleoside reverse-transcriptase inhibitors (NRTIs) + a ritonavir-boosted protease inhibitor (PI).
- The following sequence of second-line NRTI options is recommended:
  - After failure on a TDF + 3TC–based first-line regimen, use AZT + 3TC as the NRTI backbone in second-line regimens.
  - After failure on an AZT + 3TC–based first-line regimen, use TDF + 3TC as the NRTI backbone in second-line regimens.
- Heat-stable fixed-dose combinations of ATV/r and LPV/r are the preferred boosted PI options for second-line ART.
- Atazanavir (ATV/r) is preferred for patients with lipodystrophy syndrome. Please discuss with an experienced physician.
- Patients with HIV/HBV co-infection should receive a regimen with 3 NRTIs backbone plus boosted PI (eg. TDF + 3TC + AZT + LPV/r).This regimen enables effective treatment for both HIV and HBV.

TABLE 4.6: SUMMARY OF PREFERRED SECOND-LINE ART REGIMENS FOR ADULTS

Target population	Preferred second-line regimen	
**Adults and Adolescents (≥10 years)**	If d4T or AZT was used in first-line ART	**TDF + 3TC + ATV/r or LPV/r**
	If TDF was used in first- line ART	**AZT + 3TC + ATV/r or LPV/r**
**Pregnant women**	**Same regimens recommended for adults and adolescents**	
**HIV and TB Coinfection**	**Same NRTI backbones as recommended for adults and adolescents plus super-boosted LPV/r**<br>(LPV/RTV 400 mg/400mg twice daily *or* LPV/r 800 mg/200 mg twice daily)	
**HIV and HBV Coinfection**	**AZT + TDF + 3TC + ATV/r or LPV/r**	

Note: For all patients on Emtricitabine (FTC), Lamivudine can be given as both drugs have the pharmacological properties.

Second-line ART for children (including adolescents)

- After failure of a first-line NNRTI-based regimen, a boosted PI plus two NRTIs are recommended for second-line ART; LPV/r is the preferred boosted PI.
- After failure of a first-line LPV/r-based regimen, children younger than 3 years should remain on their first-line regimen, and measures to improve adherence should be undertaken.
- After failure of a first-line LPV/r-based regimen, children 3 years or older should switch to a second-line regimen containing an NNRTI plus two NRTIs; EFV is the preferred NNRTI.Such children MUST have viral load monitoring after switch with resistance testing for a detectable viral load.
- After failure of a first-line regimen of ABC or TDF + 3TC, the preferred NRTI backbone option for second-line ART is AZT + 3TC.
- After failure of a first-line regimen containing AZT + 3TC, the preferred NRTI backbone option for second-line ART is ABC or TDF + 3TC.

TABLE 4.7:SUMMARY OF RECOMMENDED FIRST- AND SECOND-LINE ART

	Children	First-line ART regimen	Second-line ART regimen
LPV/r-based first-line regimen	Younger than 3 years	ABC + 3TC + LPV/r	No change[a]
		AZT + 3TC + LPV/r	
	3 years and older	ABC + 3TC + LPV/r	AZT + 3TC + EFV
		AZT + 3TC + LPV/r	ABC + 3TC + EFV
NNRTI-based first-line regimen	All ages	ABC + 3TC + EFV (or NVP)	AZT + 3TC + LPV/r[b]
		TDF+ 3TC + EFV (or NVP)	
		AZT + 3TC + EFV (or NVP)	ABC + 3TC + LPV/r[b]

[a]No change is recommended unless in the presence of advanced clinical disease progression or lack of adherence specifically because of poor palatability of LPV/r. In this case, switching to a second-line NVP-based regimen should be considered.
[b]ATV/r can be used as an alternative to LPV/r in children older than 6 years.

SECTION 4.3 THIRD-LINE REGIMENS

Lesotho's ART programme is now 9 years old and although majority of patients are on first-line regimens, there is a significant number of patients on second-line regimens. The country's goal is to maintain patients on successful first-line for as long as possible.If there is evidence of treatment failure while on a first-line regimen switching to a second-line regimen should be done with the same principle in mind. It is likely that as the programme scales up, more patients will fail second-line treatment and will require 3rd line ART.

Third line drugs for Lesotho will be as follows: Darunavir (DRV), Ritonavir (RTV) as a pharmacokinetic booster, Raltegravir (RAL) and Etravirine (ETV).

Patients on a failing second-line regimen with no new ARV options should continue on a tolerated regimen.

CHAPTER 5: PATIENT MONITORING

Monitoring patients on ART

Clinical assessment and laboratory tests play a key role in assessing individuals before ART is initiated and then monitoring their treatment response and possible toxicities of ART.Before initiation of ART, monitoring will help to:

- Decide when to initiate ART
- Decide which ART regimen to use

After initiation of ART, monitoring will help to:

- Manage any possible side effects early, before they become serious
- Assess the efficacy of treatment
- Detect problems with adherence and/or identify treatment failure

SECTION 5.1: CLINICAL MONITORING

Clinical assessment should be the primary tool for monitoring patients both before and after initiation of ART.

Before starting ART, it is of utmost importance to rule out and treat opportunistic infections (OIs), especially active TB.

After starting ART, clinical assessments should be done by a doctor or nurse at 2 weeks, 1 month, 2 months, 3 months, 6 months after initiation, and at least every 6 months thereafter *(see Table 5.1)*.More frequent monitoring may be required if clinically indicated or if poor adherence to ARVs is noted.

A focused history and physical assessment should be performed during routine visits.Important features of regular clinical assessments should include:

- Monitoring of:
  - Weight (done at every visit)
  - Height (in children; done every 3 months)
  - Blood pressure
  - Head circumference (in children < 3 years; measured every 3 months)
  - Developmental status in children
  - Nutritional status in children
- Diagnosis and management of new illnesses
  - OIs, including tuberculosis, which may suggest immune reconstitution syndrome or treatment failure
  - Other co-morbidities, including STIs, Hepatitis B, hypertension, diabetes mellitus, substance abuse, psychiatric illness, etc.
- Medication review
  - Side effects
  - Adherence and dosing
  - Other medications including traditional medicines that may interact with ARVs
- Early diagnosis of pregnancy
- Changes in the social situation that might affect adherence to ART

TABLE 5.1:MONITORING SCHEDULE FOR PATIENTS ON ANTIRETROVIRAL THERAPY

ARV Regimen	Before ARVs are started (Baseline)	Day of Initiation	Wk 2	Mo 1	Mo 2	Mo 3	Mo 6	Mo 9	Mo 12	Every 6 mos
All Regimens	Rule out active TB (sputum and CXR if coughing)									
	Adherence		X	X	X	X	X	X	X	X
	Treatment Assistant	X					X	If adherence problems		
	Ask about symptoms of possible side effects		X	X	X	X	X	X	X	X
	Clinical exam (including weight)	X	X	X	X	X	X	X	X	Every 3 mos in children
TDF/3TC/EFV	CD4, ALT, FBC, Creatinine (Cr)			Hb*			CD4, Cr, VL		CD4, Cr, VL	CD4, Cr
TDF/3TC/NVP	CD4, ALT, FBC, Creatinine		ALT+	Hb*	ALT	ALT+	CD4, VL, ALT, Cr		CD4,VL, ALT, Cr	CD4, ALT, Cr
AZT/3TC/EFV	CD4, ALT, FBC			Hb	Hb		Hb, CD4, VL		Hb, CD4, VL	Hb, CD4
AZT/3TC/NVP	CD4, ALT, FBC		ALT+	Hb	Hb, ALT	ALT+	Hb, ALT, CD4, VL		Hb, ALT, CD4, VL	Hb, ALT, CD4
ABC/3TC/EFV	CD4, ALT, FBC			Hb*			CD4, VL		CD4, VL	CD4
ABC/3TC/NVP	CD4, ALT, FBC		ALT+	Hb*	ALT	ALT+	ALT, CD4, VL		ALT, CD4,VL	ALT, CD4
All Pregnant women	In addition to above, pregnant women should receive monthly clinical exams, and extra Hb testing (at 2 weeks, and monthly thereafter)									
Any regimen containing NRTIs	Check lactate level when symptoms or signs suggest high lactate (or lactic acidosis)									
Any regimen containing PI	Glucose and lipids (both done fasting) should be checked at baseline and when clinically indicated									

+All adults with CD4 between 250-350 cells/mm^3 are at increased risk of NVP-related hepatotoxicity, and should have ALT monitoring more frequently (extra ALT tests at 2 weeks and 3 months).
**All patients with abnormal Hb at baseline should have repeat measurement to ensure correction.*

SECTION 5.2: LABORATORY MONITORING

Guiding principles:

- Laboratory monitoring is not a prerequisite for the initiation of ART.
- CD4 and viral load testing are not essential for monitoring patients on ART.
- Symptom-directed laboratory monitoring for safety and toxicity is recommended for those on ART.
- Use viral load in a routine approach, measured at 6 and 12 months after ART initiation, and annually thereafter, with the objective of detecting failure earlier than would be the case if immunological and/or clinical criteria were used to define failure.

Baseline Laboratory Investigations

Laboratory monitoring should complement clinical assessments.Baseline laboratory tests will help to determine the regimen a patient should be initiated on. However, lack of the capacity to perform laboratory tests should not preclude a person from starting ART.

The following baseline laboratory investigations should be obtained prior to starting ART:

a. CD4 count for immunologic staging
b. Cryptococcal antigen screening for adolescents and adults with CD4 count <100 cells/mm^3
c. VDRL or RPR to screen for Syphilis if 12 years or above
d. HBsAgand Hepatitis C serology
e. Haemoglobin or FBC
f. ALT and Creatinine, urine dipstick (glucose, protein)
g. Pregnancy test
h. Blood glucose, cholesterol, triglycerides for PI-based regimens

Routine Laboratory Investigations

The following laboratory tests should be performed **routinely** depending on the regimen the patient is on(See Monitoring Schedule: Table 6.1):

- If on AZT, Haemoglobin (Hb) should be checked at 1 month, 2 months, 6 months, and every 6 months thereafter.
- If on NVP, ALT should be checked at baseline, 2 months, and 6 months after initiation.Thereafter, check ALT based on clinical suspicion of hepatotoxicity.
- If on Tenofovir (TDF), perform urine dipstick every six months.If urine protein detected, check serum creatinine (and calculate rate of creatinine clearance) every 6 months.
- Inability to perform creatinine clearance should not be a barrier to TDF use. Creatinine clearance monitoring is needed in those with underlying renal disease; of older age groups; and with low body weight or other risk factors such as diabetes or hypertension.
- HBsAg should be performed to identify HIV/HBV coinfection and who should initiate TDF-based ART.
- For individuals with HIV/HBV or HIV/HCV co-infection, it is recommended that liver enzymes be monitored 1 and 3 months after ART initiation.
- CD4 counts should be checked every 6 months to monitor immune status. Monitor CD4% for children under 5 years of age.
- Measure viral load 6 and 12 months after ART initiation and annually thereafter.If a patient is switched to a 2nd-line or 3rd- line regimen, measure viral load 6 and 12 months after the switch and annually thereafter.

Additional laboratory tests can be requested based on clinical assessments but should only be done if the results will guide the patient's management.Such tests include but are not limited to:

- Lactate assay, if the patient is on a NRTI for > 4 months and losing weight, and/or having other symptoms that suggest hyperlactatemia
- Glucose and lipid assays, if the patient is taking a PI, such as Lopinavir/ritonaviror Atazanavir/ritonavir
- Baseline full blood count at initiation.If Hb is checked using point-of-care testing, an FBC should be sent for any Hb < 8 g/dL.
- Full liver function tests for any elevated ALT result.

Calculation of Creatinine clearance in ml/min using Cockcroft Gault Equation

Male: $$\frac{1.23 \times (140\text{-age}) \times \text{wt in Kg}}{\text{Creatinine in micromols/L}}$$

Female: $$\frac{1.04 \times (140\text{-age}) \times \text{wt in kg}}{\text{Creatinine in micromols/L}}$$

Point-of-care testing equipment should ideally be available in all clinics to measure Haemoglobin (Hb) and glucose.Not only do such equipments allow for immediate results, but they also take some pressure off the district hospital laboratories, which have to cope with an ever-increasing load of specimens.

Measuring Efficacy of Treatment

The effectiveness of ART may be monitored by assessing **clinical improvement, immunologic function (CD4 count/CD4%), and HIV viral load (VL).**However, virologic monitoring is the gold standard for monitoring ART efficacy.It is necessary to make an assessment of response to treatment through regular careful clinical examinations backed where possible by simple laboratory tests.

- Viral load is the preferred monitoring approach to diagnose and confirm ARV treatment failure.
- If viral load is not routinely available, use CD4 count and clinical monitoring to diagnose treatment failure.

Clinical monitoring

Monitoring ART in adults and adolescents

The following clinical indices suggest that the patient is responding to ART:

- The patient feels better and has more energy to perform daily tasks.
- The patient is gaining weight (record the patient's weight at each visit).
- There is an improvement in symptoms and signs of the original presenting illness.
- The patient is free of new moderate or severe infections.

Monitoring of ART in children

In children, growth and development are important clinical monitoring indicators and are assessed using growth charts.

Clinical assessment involves the following:

- Always check the child's and caregiver's understanding of ART as well as anticipated support and adherence to ART.
- Always check for symptoms of potential drug toxicities.
- Always assess for treatment failure (i.e. reassessment of clinical stage).

Important signs of infants' and children's response to ART include the following:

- Improvement in growth—in children who have been failing to grow
- Improvement in neurological symptoms and development—in children with encephalopathy or who have been demonstrating delay in the achievement of developmental milestones
- Decreased frequency of infections (bacterial infections, oral thrush, and/or other OIs)

Immunologic (CD4) monitoring

With ART, immune recovery is expected with improvement in the CD4 count.Monitoring of CD4 serves as an important proxy of ART effectiveness.A falling CD4 count may indicate intercurrent illness or OI, poor adherence, or treatment failure due to resistance.Any patient with two falling CD4s needs thorough clinical review to identify any untreated illnesses or OIs.A viral load should also be sent.

Virologic (HIV viral load) monitoring

The VL usually decreases to undetectable levels within **six months** of 95-105% adherence to ART. The VL measurement is useful in assessing treatment failure. Any viral load over 1000 copies/ml MUST stimulate additional investigations and adherence support.Viral load should be tested 6 months after initiating ART, at 12 months, and then annually thereafter to detect treatment failure (See VL Algorithm below). If viral load testing is not routinely available, CD4 count and clinical monitoring should be used to diagnose treatment failure, with targeted viral load testing to confirm virologic failure where possible.

TABLE 5.2: WHO DEFINITIONS OF CLINICAL, IMMUNOLOGICAL AND VIROLOGIC FAILURE TO SUPPORT DECISION-MAKING FOR SWITCHING ART REGIMENS.

Failure	Definition
Clinical Failure	**Adults and Adolescents**<br>New or Recurrent clinical event indicating severe immunodeficiency (WHO clinical stage 4 condition)after 6 months of effective treatment<br><br>**Children**<br>New or recurrent clinical event indicating advanced or severe immunodeficiency (WHO clinical stage 3 and 4 clinical condition with exception of TB) after 6 months of effective treatment
Immunological failure	**Adults and adolescents**<br>CD4 count falls to the baseline (or below)<br>or<br>Persistent CD4 levels below 100 cells/mm^3
	**Children:**<br><br>**Younger than 5 years**<br>Persistent CD4 levels below 200 cells/mm^{3}or <10%<br>**Older than 5 years**<br>Persistent CD4 levels below 100 cells/mm^3
Virologic Failure	Plasma viral load above 1000 copies/ ml based on two consecutive viral load measurementsafter 3 months, with adherence support

FIGURE 5.1: VIRAL LOAD TESTING ALGORITHM

Already on ART (>6 months)

Viral load to be taken
- Now
- In 6 months
- Every 6-12 months based on age/pregnancy status
- **Perform immediate viral load for any patient presenting with clinical or immunological failure!**

Pregnant and lactating mothers and Children <5 years

Viral load to be taken
- 6 months after initiation
- 12 months after initiation
- Every 6 months

Newly initiated on ART
Switched to 2nd-line ART
Switched to 3rd-line ART

Viral load to be taken
- 6 months after initiation/switch
- 12 months after initiation/switch
- Annually thereafter

VL less than 1000
- Continue current regimen
- Repeat viral load according to schedule

VL more than 1000
- Screen patient for **OIs**
- Start enhanced adherence counseling (EAC)
- **1st EAC session** on day of result

2nd EAC session after 2-4 weeks
Continue if more EAC sessions are needed

Repeat VL
8-12 weeks after 1st EAC if EAC has been successful and adherence has improved

VL less than 1000
- Continue current regimen
- Repeat viral load according to schedule (follow from top of algorithm)

VL more than 1000
- Refer to clinician experienced in switching to 2nd line ART:
  - Gather information on patient from both clinicians and counselors
  - If ≥ 0.5 log drop*: repeat VL after 3 months
  - If still >1000 or < 0.5 log drop and if no outstanding adherence challenges: request switch to 2nd-line ART
- VL > 100,000 copies while on treatment is consistent

**Viral load is often measured on a logarithmic scale.A drop of 0.5 log is roughly equivalent to a 2/3 reduction.EgViral load of 2.3 log = 2000 copies/ml.A drop of 0.5 to 1.8 log = 650 copies/ml.On the other hand, a decrease from 12000 copies/ml to 5000 copies/ml only represents a 0.4 log drop from 4.1 log to 3.7 log.*

SECTION 5.3 MONITORING AND SUBSTITUTIONS FOR ARV DRUG TOXICITIES

Guiding principles

- The availability of laboratory monitoring is not required for initiating ART.
- Symptom-directed laboratory monitoring for safety and toxicity can be used for those receiving ART

Table 5.3 below summarizes the main types of toxicities associated with the first, second and third line ARVs, risk factors associated with the toxicities and suggests how they should be managed. See Annex 6 for additional information.

TABLE 5.3: TYPES OF TOXICITIES ASSOCIATED WITH FIRST- SECOND, AND THIRD-LINE ARV DRUGS

ARV Drug	Major types of toxicity	Risk Factors	Suggested Management
TDF	Tubular renal dysfunction, Fanconi syndrome	Underlying renal disease<br>Older age<br>BMI<18.5 or body wt <50kg<br>Untreated Diabetes Mellitus<br>Untreated hypertension<br>Concomitant use of nephrotoxic drugs or a boosted PI	Substitute with ABC
	Decreases in bone mineral density	History of Osteomalacia & pathological fractures<br>Osteoporosis or bone loss	
	Lactic acidosis or severe hepatomegaly with steatosis	Prolonged exposure to nucleoside analogues<br>Obesity	
ABC	Hypersensitivity reaction	Presence of HLA-B*5701 gene	Substitute with TDF (or AZTin children)
AZT	Anaemia, neutropaenia, myopathy, lipoatrophy or lipodystrophy	Baseline anaemia or neutropaenia<br>CD4 count ≤200 cells/mm^3	Substitute with TDF or ABC
	Lactic acidosis or severe hepatomegaly with steatosis	BMI >25 (or body weight >75 kg)<br>Prolonged exposure to nucleoside analogues	
	Vomiting - persistent		
EFV	Hepatotoxicity	Underlying hepatic disease – HBV and HCV coinfection	NVP, but if cannot tolerate any NNRTI, use boosted PIs
	Convulsions	History of seizure	
	Hypersensitivity reaction, Stevens-Johnson syndrome	Risk factors unknown	
NVP	Hepatotoxicity	Underlying hepatic disease<br>HBV and HCV coinfection<br>Concomitant use of hepatotoxic drugs<br>CD4>250 cells/mm^3 in women; and CD4 >400 cells/mm^{3}in men	Substitute with EFV, if person cannot tolerate either NNRTI, use boosted PIs
	Severe (Grade 4) skin rash and hypersensitivity reaction (Stevens-Johnson syndrome)	Risk Factors unknown	Use boosted PIs

ATV/r	Electrocardiographic abnormalities (PR interval prolongation)	Pre-existing conduction disease<br>Concomitant use of other drugs that may prolong the PR interval	Substitute with LPV/r or DRV/r.<br><br>If boosted PIs are contraindicated and NNRTIs have failed in first-line ART, consider integrase inhibitors.
	Indirect hyperbilirubinaemia (clinical jaundice)	Underlying hepatic disease<br>HBV and HCV coinfection<br>Concomitant use of hepatotoxic drugs	
	Nephrolithiasis & risk of prematurity	Risk factors unknown	
LPV/r	Electrocardiographic abnormalities (PR and QT intervalprolongation, torsades de pointes)	People with pre-existing conduction system disease<br>Concomitant use of other drugs that may prolong the QT interval	If LPV/r is used in first-line ART for children, use an age-appropriate NNRTI (NVP for children younger than 3 years and EFV for children3 years and older).<br><br>ATV/r can be used for children olderthan 6 years.<br><br>If LPV/r is used in second-line ART for adults, use ATV/r or DRV/r.<br><br>If boosted PIs are contraindicated and the person has failed on treatment with NNRTI in first-line ART, consider integrase inhibitors.
	QT interval prolongation	Congenital long QT syndrome<br>Hypokalaemia<br>Concomitant use of other drugs that may prolong the PR interval	
	Hepatotoxicity	Underlying hepatic disease<br>HBV and HCV coinfection<br>Concomitant use of hepatotoxic drugs	
	Pancreatitis	Advanced HIV disease	
	Risk of prematurity, lipoatrophy or metabolic syndrome, dyslipidaemia or severe diarrhea	Risk Factors unknown	
DRV/r	Hepatotoxicity	Underlying hepatic disease HBV and HCV coinfection<br>Concomitant use of hepatotoxic drugs	When it is used in third-line ART, limited options are available; refer to specialist.
	Severe skin and hypersensitivity reactions	Sulfonamide allergy	
ETV	Severe skin and hypersensitivity reactions	Unknown	Limited options are available
RAL	Rhabdomyolysis, myopathy, myalgia	Concomitant use of other drugs that increase the risk of myopathy and rhabdomyolysis	Limited options are available

SECTION 5.4DRUG-DRUG INTERACTIONS

Before initiating ART and while on ART, all medications which a patient is taking including traditional medicines should be reviewed.Since NVP, EFV, and LPV/r are all metabolised by the liver, drugs that induce or inhibit liver metabolism may affect drug levels.The effects of Sesotho medicines on serum levels of antiretrovirals have not been evaluated.It is therefore recommended that patients do not take traditional medicines in conjunction with antiretrovirals.

TABLE 5.4: IMPORTANT DRUG-DRUG INTERACTIONS

ARV drug	Key interactions	Suggested management
**AZT**	Ribavirin and peg-interferon alfa-2a	Substitute AZT with TDF
**Boosted PI (ATV/r, LPV/r)**	Rifampicin	Adjust the PI dose or change regimen
	Lovastatin and simvastatin	Use an alternative dyslipidaemia agent (for example pravastatin)
	Estrogen-based hormonal contraception	Use alternative or additional contraceptive methods
	Methadone and buprenorphine	Adjust methadone and buprenorphine doses as appropriate
	Astemizole and terfenadine	Use alternative antihistamine agent
	TDF	Monitor renal function
**EFV**	Amodiaquine	Use an alternative antimalarial agent
	Methadone	Adjust the methadone dose as appropriate
	Estrogen-based hormonal contraception	Use alternative or additional contraceptive methods
	Astemizole and terfenadine	Use an alternative anti-histamine agent
**NVP**	Rifampicin	Substitute NVP with EFV
	Itraconazole and ketoconazole	Use an alternative antifungal agent (for example fluconazole)

CHAPTER6: ADHERENCEAND DISCLOSURE

SECTION 6.1: INTRODUCTION

The standard clinical definition of adherence is taking 95-105% of medications the right way, at the right time.Overtime this definition has been broadened to include more factors related to continuous care such as following a care plan; attending scheduled clinic appointments; picking up medications on time; and getting regular CD4 tests.

Adherence to ART is critical for improving a patient's clinical, immunological and virologicoutcomes.Maintaining good adherence to the prescribed ARV regimen delays the onset of drug resistance, treatment failure and the need to switch to second line drugs.

Adherence is the process of taking medications in the correct amount, at the correct time and in the way they are prescribed.Proper storage of medications is another component of successful adherence.The ability to execute treatment adherence implies treatment literacy on the part of the patient/caregiver.This means that the patient/caregiver must understand both the disease process and necessary medications.

Excellent adherence results in the suppression of HIV replication and leads to lower viral loads, higher CD4 counts/percentage, improved clinical outcomes and lower risk for the emergence of resistant virus.

SECTION 6.2:ADHERENCE PREPARATION

Assuring adherence to the prescribed regimen begins with educating and counselling patients/caregivers on aspects regarding HIV and AIDS care and treatment.In preparation for initiation of HAART, 1-3 adherence sessions should be conducted in group or individual counselling sessions to help patients/caregivers understand basic HIV knowledge as well as the importance of excellent adherence.Topics to be covered during the sessions include:

- Basic HIV knowledge
  - How HIV is transmitted and not transmitted
  - Signs and symptoms of HIV
  - The difference between HIV and AIDS
  - Significance of CD4 count/percentage and viral load
- Antiretroviral therapy
  - Names of ARVs (including brand names)
  - Side effects of ARVs including the possibility of immune reconstitution syndrome
  - How and when to take ARVs
  - Importance of bringing all medications during clinic visits
  - Anticipated monitoring schedules; both clinical and laboratory,
  - Importance of adherence; taking95-105% of prescribed doses prevent resistance
  - The fact that treatment is lifelong even when one is feeling well
- Other relevant and practical issues
  - Demonstration to caregivers how to draw syrups into syringes
  - Importance of proper nutrition, safe water, immunizations, and primary care
  - Re-assessment of understanding of basic HIV knowledge and antiretroviral therapy
  - Provision of basic medicines for management of common OIs
  - Encouragement of disclosure to family/other caregivers who can support the treatment plan
  - Referral to a community based care support group

Discussion of the importance of medication adherence should begin as early as possible.

Barriers to Adherence

Barriers to adherence should be discussed with the patient/caregiver prior to initiation of ART. Adherence does not depend solely on the patient's or caregiver's ability to remember to take medications.Possible barriers include:

- Patient/caregiver medical/mental health
- Patient/caregiver's workload
- Lack of transportation; distance from the health facility; lack of access to refills
- Medication side effects
- Unavailability of food
- Migration
- Inability to afford associated medical costs
- Inconsistent caregiver
- Alcohol or other impairing drug use in patient or caregiver
- Domestic violence
- Living alone; lack of social support from family and/or friends
- Lack of disclosure of HIV status
- Illiteracy
- Poor understanding of the relationship between non-adherence and resistance
- Inadequate understanding of ARV regimen or effectiveness
- Lack of confidence in ability to adhere
- Belief in alternative medicines or religious healings

All of the above barriers may affect a patient's/caregiver's ability to maintain acceptable adherence.

Strategies to address Barriers to Adherence

Once potential barriers have been identified, attempts should be made to help patients/caregivers overcome them.Interventions and strategies include:

- Referral to community health workers; support groups; Community ART Groups
- Identifying supportive family/community members
- Linkage to social support services – transportation, food
- Use of pill boxes or reminder calendars or written schedules
- Referral for assistance with substance abuse problems
- Repeat adherence sessions to ensure appropriate understanding of key issues
- Use of pictorial education materials to assist with understanding
- Frequent clinic visits to monitor adherence closely
- Identification of treatment buddies or second caregivers for backup
- Use of tablets or capsules instead of syrups where applicable
- Incorporating taking of ARVs into the patient's lifestyle.
- Keeping medications in places where they are easily seen
- Planning ahead if overnight travel is anticipated
- Encouraging patients to attend facilities that are geographically closest to them
- Disclosure of HIV status to supportive family / community members
- Mobile ART services

Both group and individual counselling should be provided for caregivers of children.When appropriate, children should be included in counselling sessions.

Readiness Assessment

The provider team needs to assess a patient's/caregiver's readiness to initiate lifelong ART. Considerations for readiness include:

- General understanding of HIV, AIDS, ARVs, CD4 count/percentage, viral load, and their relationship with health status
- Understanding importance of keeping appointments
- Successful adherence to co-trimoxazole
- Successful adherence to TB therapy and INH prophylaxis
- Presence of support network in family/community to assist with treatment adherence and medication reminder
- Understanding roles of different household members in drug administrationand relevant household members counseled
- Discussion of adherence strategy, including medication schedule and methods for remembering
- Patient's/caregiver's desire and commitment to taking lifelong therapy
- Household conditions of drug storage met

For children, collaboration between the child, caregiver and the multidisciplinary team is paramount.The following should be considered during readiness assessment:

- Disclosure status of child
- Commitment of the caregiver(s)
- Cooperation of the child
- Skills for monitoring and supporting adherence by Community Health Workers
- Provision of linkages to community support structures

ARV treatment for children is complicated by:

- Developmental stage/age of the child
- Caregiver-child interaction
- Psychosocial issues
- Use of syrups/inability to swallow pills/tablets
- Caregiver factors

Adolescents need specific support to maintain adherence.

Full disclosure of HIV status and good understanding of HIV disease is important for older children and adolescents to maintain adherence.However, knowledge by itself is not sufficient.Linkages to Adolescent-friendly services – clinical and psychosocial – are other important adjuncts to successful therapy.

A supporter or treatment buddy is strongly encouraged for adults but is not a pre-requisite for the initiation of treatment. It is the responsibility of the health facility to identify a supporter who should live within a walking distance of the patient's home and be older than 12 years.

ART should be started once readiness has been agreed between the patient and the health care provider.

The patient's readiness to start ART should be documented prior to initiation of therapy. It is important to thoroughly assess and address the patient's psychosocial and economic issues as part of adherence counselling.An adherence contract including consent for home visits for future adherence assessments should be signed by adult patients and caregivers (in the case of children) prior to starting ARV treatment.

"Drugs donot work in patients who donot take them;" *New England Journal of Medicine, 353(5), 2005.*

Consequences of poor adherence include:

- Incomplete viral suppression
- Continued destruction of the immune system and decrease in CD4 cell count

- Progression of disease
- Emergence of resistant viral strains
- Limited future therapeutic options and higher costs for the individual and the programme

Excellent adherence to the first regimen has the best chance of long term success

SECTION 6.3: MAINTAINING ADHERENCE

Adherence is a lifelong process and continued assessment and education must be done at every opportunity to ensure the success of ART.At each visit, adherence must be assessed using the following parameters:

- ARV pill count or suspension return:
  - In order to perform pill counts, the pharmacy must document the date ARVs were dispensed and the # of pills dispensed.Document in the bukana.

$$\text{\% Adherence} = \frac{\text{(\# Pills taken)}}{\text{(\# Pills prescribed)}} = \frac{\text{(\# Pills given) - (\# Pills remaining)}}{\text{(Daily dose) x (\# Days since refill)}} \times 100$$

Example:

On January 1, a patient received 60 pills of AZT/3TC/NVP. Her prescribed dose was 1 tablet of AZT/3TC/NVP in the morning and 1 in the evening (2 per day).

The patient returns for a refill on January 27th (26 days since prescription filled). You count 10 pills left in the bottle. Therefore, she has taken a total of 50 pills (60-10 = 50). She was supposed to take 2 pills per day multiplied by 26 days. She should have taken 52 pills. She missed 2 doses of medication (4%).

$$\text{\% Adherence} = \frac{\text{(60 Pills given) – (10 Pills left)}}{\text{(2 Pills/day) x (26 Days since refill)}} \times 100 = 96\%$$

- Quantitative questioning:
  - "How many doses of ARVs have you missed over the past 3 days?"
  - "How many doses of ARVs have you missed over the past month?"

- Qualitative questioning:
  - "What are the names of your medications?"
  - "How often do you take/give the ARVs?"
  - "What time doyou take/give the ARVs?"
  - "How many tablet/pills do you take/give for each dose?"
  - "How much syrup do you take/give for each dose?"

Remember to use a team approach: Doctor, nurse, counsellor, social worker, laboratory technician, pharmacist, family, friends, support groups, community or village health workers, caregivers, and the PATIENT need to be involved to maintain adherence.

Help the patient/family with adherence by:

- Providing education at every opportunity
- Discussing the importance of adherence at every visit
- Asking the patient/family to name or describe the specific medications (colour, #, size, or amount given)
- If any doses have been missed, ascertain the reason

- Ask the patient/family to update you on living conditions and location
- If you expect problems and non-adherence; plan and schedule follow-up

Monitoring and Support

- Pill count
- Emphasize the importance of honest reporting
- Identify barriers to adherence (timing, work, food, etc.)
- Identify and reinforce effective and successful strategies
- Address psychosocial support
- Provide adherence aids

Dealing with Poor Adherence

If adherence is questionable (<95%):

- Repeat adherence counselling
- Increase frequency of monitoring
- Identify barriers to adherence and assist with interventions/strategies

If adherence continues below 95%, consider interrupting ART, while continuing to address adherence barriers.Stopping treatment is a last resort and should be done in consultation with an experienced physician.Remember not to stop all drugs at once if using an NNRTI-based regimen; stop NVP or EFV while continuing the backbone (e.g. AZT/3TC, ABC/3TC, or TDF/3TC) for one more week in order to reduce the risk of NNRTI resistance.Re-start treatment once barriers have been identified and accordingly addressed.

Factors that may contribute to poor adherence in children include:

- Caregiver illness
- Presence of multiple caregivers
- Holiday/vacation travel for patient; caregiver travel
- School attendance
- Extended family visits
- Poor communication among family members or between parents regarding the child's HIV status

Other adherence considerations for children include:

Infants and young children – family needs:

- Emotional and physical support for caregivers
- Have at least 2 people knowledgeable about the child's medication and available to administer
- Help the family to create a realistic medication schedule

School-age children:

- Teach them how to count / measure medication
- Help them discover foods that make medications more palatable

Older Children and Adolescents:

- Caregiver's control over the child's treatment should be more subtle (one to one; not a public issue)
- Ongoing supervision of medication by caregiver
- Individual counselingwith adolescents
- Identify friends/ peer support groups/ older children willing to help
- Provide discreet pill boxes for social events
- Use role play for problem solving

Adherence Counseling must be:

- Continuous and repetitive; at every visit
- Personalised: Tailored to the needs and situation of each child/family
- Universal: Reinforced by all health care providers

TABLE 6.1: UNDERSTANDING AND MONITORING ADHERENCE AND TOOLS TO IMPROVE IT

**Reason for non-adherence relating to drugs**	Poor palatability and unpleasant flavour
	Amount of pills/solution volume
	Frequency of dosing
	Nausea
	Fear of adverse effects (particularly if prior bad experience)
	Child's refusal
**Reason for non-adherence relating to the family**	Lack of disclosure in the family and to the child
	Strangers or visitors in the house
	Parental/caretaker illness, mental health, drug/alcohol abuse
	Lack of belief in the value of the treatment
	Responsibility for giving the medication residing with a specific member of the family
	Poor understanding/knowledge
	Denial
	Lack of food security
	Lack of funding/transport to return to the clinic
**Tools for the parent**	Colour coded bottles and syringes
	Pillboxes
	Diary cards to use as memory aid
	Encourage use of alarms (i.e. in cellular phones)
	Link medication to specific times e.g. meals or television programmes
	Make use of treatment supporters in the community
	Regular visits to therapeutic counsellors
	Early switch to pills from syrups/suspensions
	Treatment buddies
**Tools to measure adherence**	Calculate adherence by measuring the drugs returned (drugs dispensed – drugs returned)/(prescribed)x100
	Check for late returns to both the clinic and the pharmacy
	Ask about problems with specific drugs
	Look at diary cards
**The clinic should:**	Stress adherence at every visit
	Assist with disclosure within the family and to the child
	Help explain to children why they must take the drugs
	Assist with financial and food security through grants and referral to appropriate NGOs
	Support groups; tracing for missed appointments

SECTION 6.4: DISCLOSURE

Definition: Disclosure is a situation where information about a client's HIV sero-status is shared with one or more people (spouse, children, parents, friends, caregiver or employer).A counsellor can help a client develop a plan to share information about his/her HIV status. This involves exploring the options of whom and when to tell. Disclosure is important for promoting the client's adherence to treatment, prevention and care plan.

All patients should be encouraged to disclose their status to family, sexual partners, household members and community members.Appropriate disclosure can help a patient develop a reliable support network, which can be crucial to successful

adherence.Furthermore, disclosure can help fight stigma and encourage others within the family and community to “know their status”.

Benefits of Disclosure

Disclosure can help reduce stigma and discrimination

- Enables an individual to begin with the issue of reducing transmission and getting support
- Promotes easy access to care, support and treatment services as well as adoption of safer behaviour to protect family and partners
- Creates a sense of empowerment and control over the infectionsince the person is able to talk with friends or counsellors for advice and support
- Client can feel confident and no longer has to worry about having to disclose
- Client may be able to influence others to test and avoid infection
- Openness about HIV status can stop rumours and suspicion.

Skills for supporting clients to disclose include assessing the client’s readiness for disclosure:

- Make sure that it is what the client wants to do and assist him/her to plan
- Help the client to take time to make a decision

Assist clients to disclose by encouraging them to:

- Take time to accept their status
- Ensure that they are ready and comfortable to disclose
- Choose someone they can trust and who is likely to support them.
- Choose a time when the person to be disclosed to has enough time to listen and is in a goodmood
- Choose a place that is comfortable and private
- Think about how the person will react and plan for their possible response

Empower the client to disclose appropriately and safely by:

- Providing the client with information and support that can help him/her live positively
- Emphasising the need for discussingwith sexual partners who need protection against infection.
- Facilitating Role Plays such as ‘empty chair’ rehearsals where the individual client practices disclosure alone but pretending that the person is sitting next to him/her in an empty chair
- Emphasising the importance of speaking calmly and clearly

For further details on disclosure, refer to the HTC Training Manual.

Disclosure in Children

Informing the child should be age appropriate and encouraged for all children.Informing an adolescent ≥10 years of their positive HIV status prior to initiation of treatment is especially important to ensure adherence.

- Ensure that the child’s HIV status is known. The child should hear about HIV from the caregiver. The healthcare worker should support the caregiver to provide timely disclosure and appropriate information.
- Honesty is important in child-caregiver relationship
- Children often know the truth before we expect or think they do
- Children often cope with the truth better than we anticipate
- Secrecy may be associated with increased behavioural problems
- Provide the child with a sense of control over his/her life

- The child should know why he/she goes to the clinic and has blood taken regularly
- Remember that it is children's right to know their status
- Give the child permission to talk openly about HIV with caregivers

Children informed about their diagnosis have better coping mechanisms and higher self-esteem than children who are not disclosed to.In addition, children and adolescents who have been disclosed to tend have better adherence.

Source:Committee on Pediatric AIDS. Disclosure of illness status to children and adolescents with HIV infection.Pediatrics.Jan 1999.

Disclosure and discussion of the child's illness forms an essential part of regular follow up.An age appropriate disclosure process and plan should be established for all children.It is a process and not a single event.Disclosure should be done by the caregiver with assistance from the clinical team.

Process of Disclosure

"Disclosure of HIV infection status to children and adolescents should take into consideration their age, psychosocial maturity, the complexity of family dynamics and the clinical context."

Committee on Pediatric AIDS.Disclosure of illness status to children and adolescents with HIV infection.Pediatrics. Jan 1999.

What the caregiver says during the process of informing a child depends on the following:

- Age of the child
- Maturity of the child
- What the patient already knows
- Personality of the child
- Illnesses the child has had
- Whether the patient is on treatment or not
- Health of others in family
- Recent stressors

How disclosure should be done:

- Private location
- Planned in advance
- Tell the child who they can talk to about their status
- Progressive informing is preferable to "all at once."
- Provide follow-up support

Disclosure Guidelines by Age

For young children:

- Simple information in a language they can understand
- Discuss**:**
- Nature of illness
- How they can care for themselves
- The near future

Note that diagnosis and prognosis are not a priority at this stage.

For school age children:

- It is recommended that they should be informed of their status
- Discuss and plan disclosure with parents
- First determine what they already know; may ask if they know why they are

- coming to clinic/getting blood drawn
- Need for correct child assessment
- Information should be specific
- Provide moderate amount of information instead of all at once
- Assist in developing coping mechanisms
- Talk about who/what they can tell others

For adolescents:

- Should be informed of their status
- Discuss all aspects of the disease:
    - Basic nature of the HIV virus and disease progression
    - Transmission and Prevention
    - Diagnosis and Prognosis
    - Self-care and self-medication
    - Drug Resistance
    - Living Positively and Normality
    - Sexual health education

Expect the following possible feelings after disclosure:

- Shock
- Anger
- Sadness/Depression
- Fear
- Confusion
- Rejection
- Isolation
- Relief
- Acceptance

It is important to help children cope with their diagnosis, care and treatment. Ways to help children cope include:

- Problem-solving with the patient
- Empowering them
- Help the patient take one step at a time
- Reassurance
- Comfort

CHAPTER7: OPPORTUNISTIC, CO-INFECTIONS AND CO-MORBIDITIES

SECTION 7.1: MANAGEMENT OFOPPORTUNISTIC AND CO-INFECTIONS

Introduction

People living with HIV and AIDS are at a higher risk of developing infections and cancers due to their immunocompromised status.The term **co-infection** is used to refer to infections that can interact with HIV to alter its natural history and vice versa.

The term **opportunistic infection** refers to an infection caused by a pathogen that is typically harmless to a normal host but causes severe disease in patients with HIV infection.Opportunistic infections are often associated with advanced or severe immunodeficiency and usually represent WHO clinical stage 3 or 4.Due to an infant's immature immune system, opportunistic infections may occur despite a high CD4 count or percentage.Hence HIV-infected children are more susceptible and vulnerable to severe life threatening opportunistic diseases and must be monitored closely and treated aggressively.

Due to an infant's immature immune system, opportunistic infections may occur despite a high CD4 count or percentage.

Common HIV-associated illnesses are often the presenting clinical manifestations that may lead to the diagnosis of HIV.Although TB is the most common co-infection and is responsible for the highest number of deaths, there are other diseases that should be considered and monitored in patients with HIV.Diagnosis and treatment of opportunistic infections in HIV-infected patients is an essential component of their package of care.Table 7.1 below presents common adult and paediatric OIs; their major presenting signs and symptoms; diagnostic investigations; and subsequent management. Specific infections are discussed in more detail in the remainder of the chapter.

TABLE 7.1- COMMON OPPORTUNISTIC INFECTIONS

Opportunistic Infection	Major Presenting Symptoms	Prophylaxis	Diagnosis	Management	Comments
Oral candidiasis (thrush)	White spots or plaques in the mouth, painful	None	Clinical	Nystatin susp Miconazole oral gel Fluconazole 200-400 mg stat	Nystatin troches may be used but can be less effective than a single dose of fluconazole or Miconazole
Vaginal candidiasis	Vaginal itching, white creamy discharge	None	Clinical KOH prep	Nystatin or clotrimazole pessaries Fluconazole 200-400 mg stat	Nystatin pessaries are used but can be less effective than a single dose of fluconazole
Oesophageal candidiasis	Retrosternal pain and/or vomiting Difficulty in swallowing	None	Clinical	Fluconazole 200-400 mg daily for 14 days (6 mg/kg daily for children)	

Opportunistic Infection	Major Presenting Symptoms	Prophylaxis	Diagnosis	Management	Comments
*Pneumocystis jiroveci*Pneumonia(PCP)	Sub-acute shortness of breath; dry cough; fever; hypoxia; auscultation – normal or rales	CTX	Clinical; Chest x-ray	Cotrimoxazole two 960 mgtablets tds for 21 days + folic acid (120 mg/kg/day divided QID)	If dyspnoea is severe and the patient's clinical status critical, add prednisone 0.5-1 mg/kg/day
Bacterial pneumonia	Fever, cough, fast breathing, acute onset	CTX	Clinical; chest x-ray	Amoxicillin 500mg TDS for 10 days (50 mg/kg/day) Doxycycline 100mg BD for 10 days	Admit and give IV antibiotics if severe respiratory distress
LIP, Chronic lung disease	Chronic cough, lymphadenopathy, finger clubbing		CXR	ART Antibiotics and salbutamol for symptomatic flares	LIP is a paediatric disease LIP-reticulonodular patter on CXR CLD – bronchiectasis, cystic changes
MAC/MOTT	Varied Abdominal pain; cough; malaise	Azithromycin	Culture	Rifampicin Ethambutol Clarithromycin	Associated with very low CD4
Toxoplasmosis	Headache, fever, seizure, focal neurologic signs (facial droop, hemiparesis), confusion	CTX	Clinical Head CT: ring-enhancing lesions with oedema	Pyrimethamine plus sulfadiazine plus folinic acid OR Cotrimoxazole (60mg/kg/day) for 6 weeks	Consider steroids to reduce oedema
Bacterial meningitis	Fever, headache, stiff neck, vomiting, bulging fontanelle	CTX	Lumbar puncture Head CT for focal neurologic signs	Ceftriaxone 100mg/kg daily or Chloramphenicol 25 mg/kg QID	Consider head CT if no improvement or continued fevers
Isospora Crypto-sporidium Microsporidium	Persistent diarrhoea	CTX	Stool iodine stain	ART	Ensure good nutrition
Giardia	Diarrhoea, bulky, foul-smelling stool, flatulence	CTX	Stool iodine stain	Metronidazole 400mg (10mg/kg) TDS x 5 days	
Typhoid	Fever without a focus, abdominal pain, diarrhoea or constipation		Blood or stool culture	Ciprofloxacin 500mg twice daily x 7 days	Complications – peritonitis, perforation

Opportunistic Infection	Major Presenting Symptoms	Prophylaxis	Diagnosis	Management	Comments
Dysentery	Bloody diarrhoea, abdominal pain, fever, vomiting		Clinical Stool culture	Ciprofloxacin 500mg twice daily x 3 days	Additional antibiotics based on stool culture Ensure hydration
Orolabial HSV	Painful oral or pharyngeal ulcers	Acyclovir	Clinical	Acyclovir 400mg (25mg/kg) TDS x 10 days	Prophylaxis only for recurrent episodes (>6/year)
Parotitis	Swelling of parotid gland; pain with mouth movement		Clinical	Amoxicillin 500mg (50mg/kg/d) TDS x 10 days	
Acute tonsillitis or otitis media or gingivitis	Fever, pain, swollen tonsils, purulent drainage	CTX	Clinical	Amoxicillin 500mg (50mg/kg/d) TDS x 5-7days	If suspect epiglottitis, admit for IV antibiotics
Acute necrotizing ulcerative gingivitis	Ulcerative gingivitis with soft tissue loss of cheek and gums, teeth		Clinical	Ampicillin 25mg/kg QID *plus* Gentamycin 7.5 mg/kg daily *plus* Flagyl 10mg/kg TDS	Involve Dental for debridement and reconstruction
PPE Pruritic papular eruption	Itchy papules, 2mm to 2cm		Clinical	Antihistamine Topical steroids if no response	
Scabies	Itchy rash Burrows and papules in webs of fingers, wrists		Clinical KOH prep	Benzyl benzoate applied from neck down overnight and repeated in 1 week Ivermectin 6g stat	Boil clothing and bedclothes Treat family
Varicella (chickenpox)	Itchy popular rash in crops		Clinical Tzank smear	Acyclovir 20mg/kg QID	Isolate away from other immune suppressed children
Herpes Zoster	Painful vesicles, dermatomal distribution		Clinical Tzank smear	Acyclovir 800mg (20mg/kg) QID x 7-10 days	Monitor for post-herpetic neuralgia
Molluscum	Umbilicated lesions		Clinical	ART	
Tinea (ringworm)	Round scaly itchy lesions with raised edges		Clinical KOH prep	Clotrimazole cream twice daily (body)6 wks Griseofulvin 20mg/kg daily (scalp) x 6 wks	Monitor LFTS if on griseofulvin, ART, ATT Give cloxacillin for superinfected lesions
Kaposi Sarcoma	Reddish-purple or hyperpigmen-ted dark flat or raised lesions		Clinical Biopsy	ART Chemotherapy (Bleomycin, Thalidomide, Vincristine)	Refer all children with KS to oncology

Opportunistic Infection	Major Presenting Symptoms	Prophylaxis	Diagnosis	Management	Comments
	on skin or mucous membranes				
Syphilis	Painless genital lesions, rash		Clinical VDRL, RPR	Benzathine pencillin 2.4 MU IM weekly x 3	Treat partner
Gonorrhoea, Chlamydia	Burning urethral discharge Vaginal discharge		Clinical	Ceftriaxone 250mg IM stat *or* Ciprofloxacin 500mg po stat **plus** Doxycycline 100mg twice daily x 7 days *or* Erythromycin 500mg QID x 7 d	Treat partners Do not use ciprofloxacin or doxycycline if pregnant
Genital HSV	Painful anal or genital ulcers		Clinical	Acyclovir 400mg 5x daily x 10 days	
HPV/genital warts	Painless, raised fleshy lesions	HPV vaccine	Clinical	Podophyllin 0.5% twice daily on 3 consecutive days weekly x 4 weeks	Surgical excision or curettage may be needed
Cervical cancer	Vaginal bleeding	HPV vaccine	PAP smear/ VIAC Biopsy	Colposcopy Hysterectomy	Early diagnosis improves outcomes
HIV advanced nephropathy (HIVAN)	Sub-acute (high blood pressure or oedema rare)		Protein-uria Elevated creatinine	ART Enalapril 2.5mg twice daily	Avoid TDF in renal failure
CMV	Varies Malaise, visual loss, bloody diarrhoea		Clinical	ART Gancyclovir	
Lymphoma	Malaise, swollen lymph nodes, weight loss		Biopsy	Chemotherapy	

SECTION 7.2: TUBERCULOSIS

TB and HIV are closely interrelated. TB is the leading cause of morbidity and mortality in HIV- infected patients. HIV is the single most important factor fueling the TB epidemic in settings with a high prevalence of HIV infection. Patients infected with HIV infection have a 10 % annual risk to develop TB disease compared to HIV negative persons (with a 10% life-time risk of developing TB disease). Disease occurs basically through two mechanisms:

- Reactivation of latent TB infection to TB disease due to HIV-related immunodeficiency
- Rapid progression from recent TB infection (including TB re-infection) to TB disease

Early diagnosis of TB in HIV-infected patients is critical to ensure early treatment and cure of TB, to minimize the negative effects of TB on HIV progression, and halt the TB transmission in the community. Proper case management of TB can prolong the survival of PLHIV. Likewise early diagnosis of HIV in TB patients will enable early initiation of HIV care and treatment, and this has been shown to reduce morbidity and mortality of TB/HIV co-infected patients.

Reducing the Burden of TB in PLHIV

Integration of services is essential for addressing this challenge. The following include the activities to be implemented:

- Intensified Case Finding (ICF) and prompt initiation of TB treatment
- Isoniazid Preventive Therapy (IPT) for Latent TB Infection
- Infection Control (IC) for TB in congregate settings

Integration of TB services in the HIV care and treatment settings is key for delivering the services. Providers in HIV services will routinely screen for TB in all PLHIV attending HIV services and promptly provide TB treatment for identified cases.In settings where integration is not yet implemented, effective referral mechanisms must be established and maintained between the HIV and TB services so that patients are able to access the dual services without any hindrance.

ICF

TB cases are routinely detected through passive case-finding, when symptomatic patients present to health services for diagnosis and treatment.Intensified case-finding for TB (ICF) differs from passive case-finding in that screening and the diagnostic work up for TB are initiated by the provider, detecting TB earlier and reducing morbidity and mortality of TB.

HIV- infected persons are at a higher risk of developing TB disease and may present with varied atypical features making the diagnosis difficult, thus the need to implement ICF. This should be done routinely for all HIV-infected patients using the nationally approved TB screening tool.

HIV-infected patients should be screened for signs and symptoms of active TB at every clinical encounter, including when he/she is first diagnosed with HIV. ICF is essential to exclude active TB, which requires treatment with a TB treatment regimen. Diagnosis of TB should be seamlessly integrated into HIV care. Begin diagnostic procedures in the HIV clinic for all patients with a positive TB screen by sending sputum for appropriate laboratory investigations, referring for chest x-ray if indicated, etc.

ICF should be intimately linked to prompt provision of TB treatment services, with comprehensive registration of all cases.

IPT

Isoniazid preventive therapy has been shown to reduce the risk of active TB disease in persons infected with HIV. IPT should be given to all persons (over 1 year of age) infected with HIV in whom active TB has been excluded. It is used to treat latent TB infection and reduce the risk of progression to active TB disease among PLHIV. The risk of TB is particularly high during the first six months after ART initiation.

Information about TB, including IPT, should be made available to all HIV-infected patients who present for health services. Clinicians should also counsel patients about the benefits of taking IPT, side-effects associated with IPT, and need for adherence to IPT. During post-test counselling following diagnosis of HIV, patients should be screened for symptoms of active

TB and informed about the benefits of IPT. IPT is integrated within the HIV services provided by the pre-ART clinics, HIV/ART clinics, MCH/ANC clinics and paediatric clinics.

At every clinical encounter, PLHIV should be screened for signs and symptoms of TB using the Lesotho TB Screening Tool. Those who do not report any symptoms of TB are highly unlikely to have active TB and should be offered IPT if they have no contraindications to IPT. Those with one or more signs or symptoms of active TB are considered to be Presumptive TB patients and must undergo further investigations for active TB disease. Presumptive TB patients are not eligible for IPT until active TB has been excluded. Once TB has been excluded, IPT should be initiated and the patient should be followed closely.

Eligible HIV-infected patients should be initiated on IPT irrespective of the CD4 count, WHO clinical stage, and ART status. There isan additional protective benefit of concomitant use of IPT and ART. Patients who are receiving IPT and who are eligible for ART should continue IPT while initiating ART. IPT should not delay ART initiation among eligible PLHIV.

Given the high prevalence of latent TB infection in Lesotho, all PLHIV (over 1 year of age) who have no signs or symptoms of TB at the time of HIV testing and/or entry into care and who do not have contraindications to IPT should be started on IPT as soon as possible.

Initiate IPT after:

- Active TB has been excluded.
- Contraindications to IPT (i.e. active TB disease, active hepatitis, alcoholism, severe peripheral neuropathy, epilepsy, or kidney failure) have been excluded.
- Patients have been counselled on the benefits of IPT, the importance of adherence to IPT, and on the need to return should possible side-effects or signs/symptoms of TB develop.

HIV-infected patients with signs and/or symptoms of TB, or with signs and/or symptoms of active liver disease should not be offered IPT.

Patients should not be offered IPT if they report:

- Acute or chronic liver disease. Signs and symptoms suggestive of active hepatitis are: nausea, vomiting, right upper quadrant pain, jaundice, dark urine.
- Regular and heavy alcohol consumption.
- Symptoms of severe peripheral neuropathy
- History of epilepsy or convulsions.
- Kidney failure.

The absence of baseline liver function tests should not preclude the initiation of IPT. However, as all HIV-infected patients have a baseline lab assessment, the most recent ALT result should be reviewed if available.

TABLE 7.2: INTERPRETATION OF ALT LEVELS IN THE CONTEXT OF INITIATING IPT

Baseline Liver Function Tests	Course of action
Normal up to 2x the upper limit of normal (ULN) in the absence of symptoms of hepatitis	Initiate IPT, no further testing required
2-5x the ULN in the absence of symptoms of hepatitis	Initiate IPT<br>Check ALT monthly
Greater than 5x the ULN and/or symptoms of hepatitis	Do not initiate IPT

The standard IPT regimen is:

Adults
Isoniazid (INH): 300 mg/day x 6 months
Pyridoxine (Vitamin B6): 25 mg/day x 6 months

Children
Isoniazid (INH) 10 mg/kg/day (max 300 mg/d) x 6 months
Pyridoxine (Vitamin B6): 12.5-25 mg/day x 6 months

Pyridoxine should be given concomitantly with isoniazid to prevent the occurrence of peripheral neuropathy. Isoniazid preventive therapy should be given once daily for 6 months. Strict adherence to IPT is essential. If a patient has an interruption in IPT for no more than three months, he/she can be restarted if still asymptomatic. Thus in case of interruption of less than 3 months, the treatment can be completed over 9 months.

Patients on IPT should be monitored through monthly clinical assessment to include:

- Screening for symptoms and signs of active TB (i.e. cough of any duration, fever, night sweats or weight loss).
- Screening for possible side-effects of isoniazid (e.g. rash, peripheral neuropathy, convulsions, or any signs/symptoms of hepatitis including nausea and vomiting, jaundice, right upper quadrant pain and dark urine).
- Adherence to isoniazid.

If a patient on IPT develops symptoms of active TB or possible side-effects of isoniazid:

- Discontinue IPT immediately
- Investigate for active TB disease:
  - Send sputum specimen (morning) for GeneXpert or smear microscopy
  - Refer if needed to ensure that investigations are completed
- If active TB is confirmed, a full TB treatment regimen should be started
- Perform other laboratory investigations as clinically indicated

Routine laboratory monitoring of liver function tests (e.g. ALT) is not required during IPT.

However, if a patient is known to have an elevated ALT at baseline (2-5x ULN); then monthly monitoring of the ALT is indicated. An ALT should also be ordered if symptomatic hepatitis develops while on IPT. If the ALT is greater than 5x the ULN, then IPT should not be restarted and the patient should be referred for further investigations.

All other laboratory tests should be ordered as clinically indicated.

Isoniazid (INH) is safe in pregnancy and during breastfeeding. IPT should be offered to all eligible HIV-infected pregnant and breastfeeding women after TB screening and exclusion of active TB. IPT can be started at any time during pregnancy. In the event that a woman on IPT becomes pregnant, IPT should be continued. Following delivery, IPT should be continued during breastfeeding to complete the six month course of therapy

All HIV-infected children exposed to TB through household contacts but with no evidence of active disease, should begin Isoniazid Preventive Therapy (IPT).

Infants<12 months HIV infected and TB exposed should receive IPT and be followed clinically as part ofa comprehensive package of HIV care.However, children in this age range who are not TB exposed should not receive IPT.

TABLE 7.3: SIMPLIFIED WEIGHT-BASED DOSING FOR ISONIAZID 10MG/KG/DAY

Weight (kg)	Number of 100mg tablets of INH to be administered per dose	Dose given (mg)
<5	½ Tablet	50
5.1 - 9.9	1 Tablet	100
10 – 13.9	1 ½ Tablets	150
14 – 19.9	2 Tablets	200
20 – 24.9	2 ½ Tablets	250
>25	3 Tablets	300

Adapted from WHO Guidelines of Antiretroviral Therapy for HIV infection in Infants and Children: Towards Universal Access. 2010 Revision

IPT provides protective benefit to patients who have successfully completed TB treatment.

All HIV-infected patients should take IPT for six months immediately after completion of TB treatment.

IC

Persons with undiagnosed, untreated and potentially contagious TB are often seen and managed in health care settings; such frequent exposure to patients with infectious TB disease may put the health worker at risk. Furthermore HCW and staff may be immunosuppressed themselves due to HIV infection and be at higher risk of developing TB disease.

Nosocomial transmission of *M. tuberculosis* has been linked to close contact with persons with TB disease during aerosol-generating or aerosol-producing procedures, including bronchoscopy, endotracheal intubation, suctioning, other respiratory procedures, open abscess irrigation, autopsy, sputum induction, and aerosol treatments that induce coughing.

All health facilities should be made aware of the need for preventing transmission of *M. tuberculosis* especially in settings where persons infected with HIV might be encountered or might work. All HCWs should be sufficiently informed regarding the risk for developing TB disease after being infected with *M. tuberculosis*.

All health-care settings should develop a TB infection-control plan designed to ensure prompt detection, airborne precautions, and treatment of persons of confirmed TB disease. TB infection control measures can be divided into three categories namely: managerial/administrative, environmental (or engineering), and personal respiratory protection controls.See Chapter 12 for additional details.

TB/HIV Co-Infection: Clinical Presentation

The clinical picture of TB varies with the level of immunity of the patient. PLHIV are more likely to present with sputum smear-negative PTB, disseminated TB, or EPTB, especially as the immunosuppression progresses. Therefore a high index of clinical suspicion is needed to avoid misdiagnosis or delays in TB diagnosis, which may lead to increased morbidity and mortality.

TABLE 7.4: MANIFESTATIONS AND CLINICAL FEATURES OF TUBERCULOSIS

Form of TB	Symptoms	Signs
Pulmonary	Infants: fever, cough, dyspnea<br>Children: cough<br>Adolescents/adults: fever, cough, sputum production, anorexia, weight loss, night sweats, hemoptysis	Crackles, wheezing, decreased breath sounds
Pleural	Chest pain, cough, dyspnea, fatigue, anorexia, weight loss, night sweats	Decreased breath sounds, dullness to percussion, asymmetric chest movement, pleural rub, decreased vocal resonance and fremitus, bronchial breathing and egophony above the effusion
Peripheral lymphadenitis	Painless, slowly enlarging lymph nodes over wks to mos without erythema or warmth, fever, weight loss, fatigue, night sweats	
Intrathoracic lymphadenopathy	Usually none; rarely causes symptoms associated with extrinsic airway compression or trachea-esophageal fistula formation	
Disseminated, including miliary	Fever, cough, dyspnea, weight loss, anorexia, night sweats, rigors	Fever, cachexia, tachypnea, hypoxemia, abnormal lung exam, hepatosplenomegaly, lymphadenopathy, erythematous macular or papular skin lesions, choroid tubercles
Pericardial	Fever, chest pain, cough, dyspnea, orthopnea, weight loss	Hepatomegaly, jugular venous distension, pulsus paradoxus, pericardial friction rub/knock, ascites, pedal edema
Meningitis	Adults: headache, fever, neck stiffness, lethargy<br>Children: nausea, abdominal complaints, lethargy, irritability	Stage I (early): nonfocal signs<br>Stage II (intermediate): Brudzinski's and Kernig's signs, tripod phenomenon, cranial nerve palsies<br>Stage III (advanced): hemiplegia or paraplegia, hypertonia, hypertension, hemodynamic instability, decerebrate posturing, seizures, coma
Tuberculous brain abscess	Headache, fever, delirium	Cranial nerve palsies, papilledema, hemiparesis, seizures
Laryngeal	Cough, hemoptysis, odynophagia, hoarseness, pain or weakness with speaking, ear pain	Laryngeal edema and/or hyperemia; nodular swelling of interarytenoid space, aryepiglottic folds and/or epiglottis; vocal cord paralysis; laryngeal stenosis
Otologic	Painless otorrhea, hearing loss	Tympanic perforations, thickened tympanic membrane, facial nerve paralysis, bony labyrinthitis
Abdominal (enteritis, peritonitis)	Abdominal pain, weight loss, fever, weakness, nausea, vomiting, anorexia, abdominal distension, night sweats, constipation, diarrhea	Cachexia, abdominal mass, abdominal distension, "doughy" abdomen, ascites, rebound tenderness
Hepatobiliary	Abdominal pain, fever, malaise, fatigue, night sweats, anorexia, weight loss	Right upper quadrant tenderness, hepatosplenomegaly, jaundice, ascites
Kidney, ureters, and bladder	Fever, night sweats, weight loss, dysuria, flank pain, hematuria, urinary frequency/urgency, nocturia	

Form of TB	Symptoms	Signs
Fallopian tubes, endometrium, and ovaries	Pelvic pain, infertility, amenorrhea, dysmenorrheal, abnormal uterine bleeding, fever, weight loss, fatigue	Adnexal masses, uterine enlargement
Spinal (Pott's) disease	Back and neck pain, weakness, numbness, changes in gait	Flank mass, paraparesis, paraplegia, spinal deformity
Adrenal gland	Fatigue, anorexia, nausea, abdominal pain, diarrhea, arthralgias, myalgias	Orthostatic hypotension, hyperpigmentation
Mastitis	Unilateral breast lump/swelling	

Clinical Assessment in TB/HIV Co-Infection

All TB/HIV co-infected patients should be provided with comprehensive clinical assessment prior to initiation of treatment. The assessment should include:

- A review of patient history
- Physical examination
- Laboratory investigations:
    - Full blood count
    - CD4 cell count
    - Pregnancy test for women
    - Liver Function Test-ALT
- Others (as determined by the patient's presentation)

However it should be noted that lack of capacity to perform CD4 cell counts should not be a barrier to initiate HIV treatment for the TB/HIV co-infected patients.

Treatment of TB in PLHIV

TB treatment is effective for both HIV-infected and uninfected persons alike. The same regimens used for HIV-uninfected persons should apply for the HIV-infected patients, with the same duration of treatment. There is a need for prompt initiation of TB treatment because of the increased morbidity and mortality associated with dual infection.

Enhanced DOTs (support for TB and HIV by same treatment supporter) should be provided to the patients with TB/HIV co-infection, and comprehensive adherence preparation needs to be availed both to the patient and treatment supporter. This is in view of the increased morbidity and mortality (especially in the initial two months of treatment initiation), increased pill burden, and overlapping side effects from concurrent ARVs and anti-TB medicines.

CTX

In all HIV-infected TB patients, cotrimoxazole preventive therapy should be initiated as soon as possible and given throughout TB treatment. Cotrimoxazole preventive therapy substantially reduces mortality in HIV-infected TB patients.

HIV treatment for TB/HIV co-infected patients

TB/HIV co-infected patients have an increased risk of dying before TB treatment is completed, and case-fatality occurs mainly in the first two months of TB treatment. There is need to fast track these patients for both TB and HIV care and treatment alike. Delaying ART initiation increases the mortality due to HIV infection. Early initiation on ART while on TB treatment will reduce mortality and morbidity among HIV co-infected TB patients.Improved immune system functioning from ART helps to cure TB and decreases infectiousness and transmission of HIV.

Even though co-treatment is associated with drug-drug interactions, overlapping toxicities, pill burden with risk of poor adherence, and increased frequency of immune reconstitution inflammatory syndrome (IRIS), the reduced morbidity and mortality accruing from early initiation of ART far outweigh any adverse events.

When to start ART in TB/HIV co-infected patients

All TB/HIV co-infected patients should be started on ART within 2-4 weeks of TB treatment initiation, irrespective of the CD4 cell count.Laboratory investigations enhance clinical evaluation. Clinical assessment is the primary tool for evaluating patients both before initiation and after ART treatment has been initiated. Laboratory investigations can help inform which regimen to choose but are not essential for ART initiation. Inability to perform laboratory investigations should not prevent patients from being initiated on ART!

Comprehensive patient preparation should be provided in view of the needed adherence to both TB and HIV treatments. Adherence counselling should be offered on an ongoing basis.

What ART regimens to use for TB/HIV co-infected patients

The preferred 1st-line ART regimen for adults is TDF + 3TC + EFV.

Use Efavirenz (EFV) as the preferred NNRTI in patients on TB treatment.The preferred 1st line ART regimen for children is ABC + 3TC + EFV (NVP if <3 years or <10 kg).

Patients who Develop TB while on ART

If TB is diagnosed after a patient has already been initiated on ART, then start TB treatment, and switch adults who are on Nevirapine-based regimens to Efavirenz.

LPV/r and other PIs have significant interactions with rifamycins and should not routinely be used together. There may be situations when LPV/r is the only option for patients on concomitant TB treatment, in which case it should be used. If a patient is on LPV/r, add Ritonavir (RTV) in a ratio of 1:1 (LPV: RTV) to achieve the full therapeutic dose of RTV.

Drug-Resistant TB and HIV

Drug-resistant forms of TB (such as MDR-TB and XDR-TB) have been reported to be more common among HIV-infected populations in some studies. Thus, any patient not clinically or bacteriologically responding to TB treatment after two months who is receiving good DOT should have a culture sent for drug susceptibility testing (DST). For management of patients with suspected or confirmed drug-resistant TB, please refer to National TB Guidelines.

Immune Reconstitution Inflammatory Syndrome (IRIS)

Following the initiation of ART the immune system is reconstituted and begins to respond to antigens more vigorously, which may result in a paradoxical reaction with worsening symptoms and signs of TB despite a response to TB therapy.

TB IRIS can present in two ways.

- Paradoxical TB IRIS – a patient is diagnosed with TB, starts TB treatment followed by ART after a few weeks, and then develops worsening TB signs and symptoms.
- Unmasking TB IRIS – a patient is screened for TB before initiation of ART and no TB is found. The patient then starts ART, followed by onset of TB symptoms and signs.

TB IRIS usually occurs within the first 2-12 weeks of initiating ART.

The key risk factors for IRIS include the following:

- Severe immune suppression (CD4 count <50)
- High viral load (>100,000 copies/ml)
- Early initiation of ART
- Marked rise of CD4 count and fall of viral load following ART initiation
- Presence of subclinical opportunistic infections

Management of IRIS is to continue TB treatment and provide non-steroidal anti-inflammatory drugs (NSAIDs). Corticosteroids may be used in cases with severe signs. Admit all patients with danger signs. In general, do not to stop ART! The possibility of IRIS should be explained to patients prior to initiation of ART (i.e. the patient may become worse before becoming better).

Danger signs include, but are not limited to:
- Respiratory distress (RR > 30)
- Fever ($T>39^0$)
- Tachycardia (HR > 120)
- New or worsening adenitis, with obstructive symptoms

IRIS is a diagnosis of exclusion and particular attention should be paid to assess/exclude the following:
- TB treatment failure or drug resistant TB
- ART treatment failure
- Other opportunistic infections
- Side effects of TB treatment and/or ART
- Drug fever
- Other HIV-related diseases (lymphoma, Kaposi's Sarcoma)

SECTION 7.3MANAGEMENT OF CRYPTOCOCCAL DISEASE

Prevention of Cryptococcal Disease

Patients initiating ART with undiagnosed cryptococcal disease are at higher risk of early mortality than patients who are diagnosed and treated for cryptococcal disease.All patients initiating ART should be clinically screened for evidence of symptomatic cryptococcal disease – headache, neck stiffness, fever, focal neurologic signs, confusion, altered mental status, vomiting. All those who screen positive should be referred for further diagnostic work up for meningitis. Screening of asymptomatic ART naïve individuals with CD4 count <100 cells/mm3 is recommended and should be done with a *Cryptococcus neoformans* antigen test (CrAg) on serum, plasma or CSF. A lumbar puncture should be offered to individuals who screen positive for cryptococcal antigen, as a positive cryptococcal antigen may precede the onset of clinical cryptococcal meningitis by many weeks.

TABLE 7.5: TREATMENT DECISIONS FOR ASYMPTOMATIC CRYPTOCOCCAL DISEASE

Serum CrAg negative	No LP necessary. No fluconazole required.<br>Initiate ART.
Serum CrAg positive	Perform LP.<br>If CSF CrAg positive, manage for cryptococcal meningitis(Table 7.8)<br>If CSF CrAg negative, treat with Fluconazole 800mg orally once daily for 2 weeks, then Fluconazole 400mg orally daily for 8 weeks, followed by maintenance therapy with Fluconazole 200mg orally daily until CD4>200 cells/mm^3 for 6 months

Initiate ART 2-4 weeks after initiation of antifungal therapy in individuals who screen positive for serum CrAg without any evidence of disseminated cryptococcal meningitis.

Treatment of Cryptococcal Meningitis

Cryptococcal meningitis remains a major cause of death in HIV infected patients. Early diagnosis and prompt treatment is critical to improve clinical outcomes. The mainstay of treatment is rapid diagnosis, prompt initiation of appropriate antifungal therapy and management of raised intracranial pressure. Patients at greatest risk of cryptococcal meningitis are those with very low CD4 counts, and clinical suspicion must be high for all patients presenting with headaches, confusion, altered mental status.

Diagnosis of cryptococcal meningitis is made by lumbar puncture. Lumbar puncture is both diagnostic and therapeutic.Elevated opening pressure is characteristic of cryptococcal meningitis. If a manometer is not available, intravenous tubing and a tape measure may be used to measure the column of CSF fluid. CSF samples can be tested for cryptococcus by india ink staining and/or CSF cryptococcal antigen test. Sensitivity and specificity for india ink staining are not as high as cryptococcal antigen testing, and a negative test does not exclude cryptococcal meningitis in the right clinical setting.

Treat cryptococcal disease with amphotericin B based regimens. Combination therapy with amphotericin B and fluconazole is strongly recommended. In the absence of amphotercin B, high dose fluconazole can be used as alternative therapy (See Table 7.8).Therapy is characterized by a 2-week induction phase, followed by an 8-week consolidation phase, and maintenance therapy which is continued until adequate immune reconstitution is achieved.

TABLE 7.6: RECOMMENDED THERAPY FOR CRYPTOCOCCAL MENINGITIS

	Treatment phase	Regimen	Duration of therapy
**Preferred**	Induction phase	Amphotericin B 0.7-1mg/kg/day IV + Fluconazole 800mg orally once daily	2 weeks
	Consolidation phase	Fluconazole 800mg orally once daily	8 weeks
	Maintenance/Secondary prophylaxis	Fluconazole 200mg orally once daily	Until CD4 count >200 cells/mm^3 for 6 months
**Alternate**	Induction Phase	Fluconazole 1200mg orally once daily	2 weeks
	Consolidation Phase	Fluconazole 800mg orally once daily	8 weeks
	Maintenance/Secondary prophylaxis	Fluconazole 200mg orally once daily	Until CD4 count >200 cells/mm^3 for 6 months

Management of Raised Intracranial pressure

Mortality and morbidity from cryptococcal meningitis is high with a significant proportion attributable to raised intracranial pressure. Management of raised ICP is critical to ensure good clinical outcomes. If the intracranial pressures is >25cm of water, remove 10-30ml of CSF and continue with daily lumbar punctures until CSF pressures have normalized (<25cm of water). Lumbar puncture should be performed even if manometer is not available.Doctors can monitor pressure clinically.Failure to adequately manage intracranial pressures can result in persistent headache, cranial nerve abnormalities which include hearing loss, vision loss, and death.

Management of Amphotericin B associated toxicities

Amphotericin B is associated with renal tubular toxicities and can lead to electrolyte abnormalities such as hypokalemia and hypomagnesemia. It can also result in anaemia and administration-related febrile reactions.

- Amphotericin B is often provided as a powder and should be mixed with 5% dextrose water. It should never be mixed with normal saline or half normal saline as this will result in precipitation of the amphotericin B. To minimize renal toxicities, administer slowly over 4 hours.
- Prehydration with 500ml-1L of normal saline with 20mEq of potassium chloride is recommended based on the volume status of the patient.
- Provide oral potassium supplementation – e.g. 1200mg twice a day. The potassium supplementation minimizes the extent of hypokalemia that can develop. Where available, supplementation with magnesium trisilicate 500mg orally twice daily is also recommended.
- Renal function must be monitored at baseline. Monitor renal function (urea&electrolytes) twice weekly.

If the creatinine doubles, a dose of amphotericin B can be omitted, and prehydration increased to 1L of normal saline every 8 hours and creatinine rechecked. If creatinine normalizes, prehydrate with IL normal saline with 20mEq KCL and restart at amphotericin B (0.7mg/kg/day) given over 4 hours.

If repeat creatinine remains elevated or continues to increase, amphotericin B should be discontinued and high dose fluconazole 1200mg orally once daily initiated. Monitoring of haemoglobin at baseline and weekly is also recommended.

Timing of ART in Cryptococcal Meningitis

Early initiation of ART is recommended for all OIs except for intracranial OIs such as TB meningitis and cryptococcal meningitis. In cryptococcal meningitis ART can be initiated 2- 4 weeks after initiation of antifungal therapy with amphotericin B based regimens. In patients who are predominately treated with fluconazole monotherapy, ART should be initiated at least 4 weeks after initiation of antifungal therapy.

ART should NOT be commenced at the same time that amphotericin B and/or fluconazole therapy is commenced for cryptococcal meningitis.

SECTION 7.4PNEUMOCYTIS JIROVECII PNEUMONIA (PCP)

*Pneumocystis jirovecii*pneumonia is caused by a fungus, *Pneumocystis jirovecii*, which is common in the environment and does not cause disease in immunocompetent people.

Clinical presentation and diagnosis

Diagnosis of PCP is mainly clinical, and patients commonly present with sub-acute onset and progression of shortness of breath, non-productive cough and chest pain. Fever is not always present but can be high.PCP is common in HIV-infected children and may be the presenting condition.The peak age for PCP in children is six months, and any exposed infant with presumptive PCP disease needs an immediate DNA PCR to confirm HIV infection status.

Patients commonly have severe immunosuppression, CD4 <200 cells/mm^3, or CD4% <15% in infants and young children. Physical examination shows tachycardia, increased respiratory rate ± rales. Hypoxia is common, and cyanosis may be present. Auscultation may be normal in many cases and in the presence of hypoxia should raise the suspicion of PCP.

Chest X-ray findings: often bilateral symmetrical interstitial infiltrates ("ground-glass" appearance) but may be normal in up to 30% of cases.

Treatment

High dose cotrimoxazole: 120mg/kg/day in 3 divided doses for 21 days (typical adult dose is 2 double –strength tablets three times a day).

Alternatives: Dapsone 100mg once daily + Trimethoprim 5mg/kg/day TID for 21 days or Primaquine 15-30mg once daily + clindamycin 600mg IV 6 hourly for 21 days.

Patients with severe disease (PaO_2 < 70 mmHg at room air) should receive corticosteroids (prednisolone 40mg twice daily for 5 days, then 40 mg once daily for 5 days, then 20 mg once daily for the remaining 11 days of antibiotic therapy).

Secondary prophylaxis with cotrimoxazole 960mg once daily should be given until the patient is stable on ART with immune recovery - CD4 > 350 cells/$mm^3$for six months.

SECTION 7.5: OTHER HIV- ASSOCIATED ILLNESSES

Hepatitis B

TDF-3TC based regimen is preferable for patients with HIV/HBV co-infection as both drugs are also active against hepatitis B.All patients need baseline screening for hepatitis B (HBsAg) and vaccination if no infection is detected.When switching patients with hepatitis B infection to second-line regimens, close monitoring for worsening of hepatitis B status should be done.Start ART in all HIV/HBV-coinfected individuals irrespective of the CD4 cell count or WHO clinical stage.

Syphilis

Syphilis is a common sexually transmitted disease that can occur in the presence of HIV infection.All HIV-infected patients over 12 years of age should have a baseline RPR or VDRL test done and be treated with benzathine penicillin 2.4 MU IM every week for 3 consecutive weeks if the test is positive.Patients with presumed neurosyphilis need additional investigation and treatment with aqueous Penicillin G 3-4 MU IV q4 hours x 14 days.Point-of-care tests for syphilis are now available and should enable timely treatment.

Human papilloma virus

HPV has been linked to cervical cancer; a major cause of morbidity and mortality among women with HIV.For this reason, all sexually active HIV-infected women should undergo yearly cervical cancer screening.The HPV vaccine is given to all girls from 9-15 years as part of cervical cancer primary prevention.

Other Sexually Transmitted Infections

Patients with HIV are at an increased risk of contracting other STIs.Conversely, other STIs (especially ulcerative ones such as HSV) may increase the risk of HIV transmission.Patients should therefore be asked a series of screening questions at each encounter regarding the presence of genital ulcers and/or discharge and managed accordingly.Refer to National Guidelines for Syndromic Management of STIs for more details.

Immune Reconstitution Syndrome

IRIS is a phenomenon that occurs when a patient on ART begins to have immune recovery in the presence of an untreated or partially treated OI.This may lead to a paradoxical

transient worsening of symptoms, despite favourable recovery of the immunological status (CD4 count/percentage).IRIS usually occurs within the first 6 weeks to 6 months of initiating ART. All efforts must be made to diagnose and treat the offending OI.In general, do not to stop ART.The condition can be managed with proper OI treatment and non-steroidal anti-inflammatory drugs (NSAIDs).Corticosteroids may be used in severe cases.The possibility of IRIS must be explained to patients prior to initiation of ART (i.e. the patient may become worse before becoming better).Forewarning a patient of this possibility will assist with future adherence and help the patient to come early for care and management if symptoms occur. IRIS is most commonly associated with TB, toxoplasmosis, Kaposi Sarcoma, and cryptococcus.

SECTION 7.6MENTAL HEALTH PROBLEMS IN RELATION TO HIV

Mental health problems such as depression, anxiety, substance abuse and confusion are more common in PLHIV and can be a significant contributor to poor adherence to treatment.

DEPRESSION

Depression is linked to the psychological impact of HIV status on the patient or from a close relative (child, parent, spouse) and may impair adherence to treatment. Symptoms of major depression are chronic (at least 2 weeks) and impact the patient's life.

The main criteria are a pervasive sadness and a lack of interest in daily activities.Additional symptoms include:

- Significant loss of appetite.If depression is untreated, patients will also lose weight.
- Insomnia, with early waking
- Psychomotor retardation or agitation (uncommon)
- Significant fatigue
- Difficulty concentrating or making decisions
- Feeling guilty, worthless, despair
- Suicidal ideation

Treatment involves a combination of cognitive/behavioural therapy and medication.Exclude any other causes of depression: hypothyroidism, Parkinson syndrome, Efavirenz intolerance (associated with insomnia, nightmares, loss of memory), recent family death, etc.

Pharmacological treatment is often needed and speeds recovery.Many patients benefit from 3-6 month courses of therapy, but some will need longer-term treatment.Medication is encouraged for those with suicidal ideation, repeat episodes of depression and insufficient response to psychological support alone.All patients on anti-depressant medication also benefit from psychological support, and combination therapy is highly recommended.

Selective serotonin reuptake inhibitors (SSRIs) such as fluoxetine are the drugs of choice. Dosage is 10-20mg daily initially but can be titrated up to 60mg daily.Older anti-depressants such as amitriptyline may also be considered.Dosage is 25mg initially but may be titrated up to 100mg daily. Lower doses should be used in older patients.In patients with combination depression and anxiety, diazepam 5 to 10 mg/day in 2 divided doses can be added during the first two weeks of anti-depressive treatment.

Weekly consultations the first month are necessary, to follow the symptoms, the side-effects and to refill medications.It is recommended to not prescribe too many tablets initiallydue to risk of suicide. The treatment should always be stopped gradually, over a 2-week period for fluoxetine and a 4-week period for amitriptyline.

GENERALISED ANXIETY DISORDER

Symptoms: feeling tense; nervous with poor concentration; restlessness; agitation; termers; sleep disturbances; palpitations; dry mouth or dizziness.

Mostly anxiety is associated with depression.

Management:

- Detailed history taking and physical examination.
- Mental state assessment.
- Rule out medical condition mimicking symptoms like Thyroid disorders; adrenal tumours; hypertension or cardiac dysrhythmias.

Drug treatment:

- Treat underlying medical condition if found.
- SSRI- Fluoxetine/ paroxetine 20mg daily for 3-4 months; night time if insomnia Diazepam 2-5 mg at night for a week.
- Alternatively amitriptyline 25mg – 75mg daily in divided doses.

Provide counselling and support

Treatment is based on psychosocial support and counseling.Involvement in support groups is also helpful.Some patients will benefit from medication (Diazepam).

SUBSTANCE ABUSE DISORDERS

Use of alcohol or other drugs is a common reason for poor adherence.

Management involves regular support counseling.Co-morbid depression or anxiety should be diagnosed and treated.

Acute Psychosis:

Symptoms include: restlessness, agitation, shouting, aggressive and violent behaviour, running away, destructive to property, setting fire to house and property, illogical, irrational and irrelevant talk, sees and hears voice from nowhere, and poor hygiene.

Management:

- Brief history taking from relative/ escortee.
- Physical examination if possible.
- Look and note injuries.

Drug Treatment:

Inj. Haloperidol 5mg I/M.

Inj. Diazepam 5-10mg I/M; repeat inj if necessary after4-6 hrs.

Once patient is calm and cooperative do thorough physical examination and laboratory investigations including radiological investigation.

Start Oral treatment:

Haloperidol 5mg twice daily or Resperidon 2mg – 4mg daily orally in divided doses.

Watch for extra pyramidal symptoms like termers, stiffness in lounge, rolling of eye balls and excessive salivation. If there are extrapyramidal side effects, give Artane 2mg twice a day or Akineton 2mg twice a day.

Note- Patients with organic brain syndrome are very prone to develop severe side effects like EPS, tardive dyskinesia and neuroleptic malignant syndrome

CONFUSION

Confusion, disorientation in time and space,impaired consciousness, concentration problems, may all be associated with HIV infection. The cause is often organic: cerebral toxoplasmosis, meningitis or encephalitis, or medicationside-effect: Efavirenz (rare). Identification and treatment of the underlying cause is essential.Direct HIV effects will improve with ART.

HIV/AIDS related Dementia:

Symptoms: Memory impairment in abstract thinking and judgement; loss of attention and concentration; restlessness, agitation and wandering around; suspicious and accusing people around; motor imbalance, lack of coordination or urinary incontinence.

Management:

Detailed history and previous medical record and medications.

Medical examination/ Neurological examination.

Lab. Investigations- urea and electrolytes, thyroid functions, L F T, Serum B12 and folic acid level, Lumber puncture if indicated, CT / MRI of brain.

Treatment of medical condition- Urinary or respiratory tract infection, Cardio pulmonary dysfunctions, R/out sub dural haematoma.

Drug treatment:

INJ. haloperidol 2.5 – 5mg I/M; Inj Diazepam 5mg.I/M.

Start oral treatment when pt. is calm and co-operative: Haloperidol 2.5 – 5mg twice a day or Risperdal 2mg- 4mg daily orally.

Watch for Extra pyramidal symptoms if it appears then Artane2mg twice a day orally.

Other measures: frequent reminders of time and place to patient to reduce confusion; assess ability to perform daily task; reduce stress in care givers in long term management.

SECTION 7.7: MALIGNANCY AND HIV

Kaposi's sarcoma

Kaposi's sarcoma (KS) is the most common malignancy in patients with HIV infection. HIV-associated KS does not have a preferential pattern of localization and may affect all skin and mucous membranes. Lymph nodes and internal organs such as stomach, gut, lung or liver

may also be involved. The progression of HIV-associated KS is variable: the tumors can remain unchanged for months to years, or grow rapidly within a few weeks and disseminate.

Typical findings at manifestation are a few asymptomatic purple macules or nodules. Rapid growth can lead to localized pain and a discoloration of the area around the tumor as a result of hemorrhage. Further progression of the tumor can lead to central necrosis and ulceration. The tumors may bleed easily. Plaque-like and nodular KS lesions often become confluent and can be accompanied by massive lymphoedema. In the oral cavity, the hard palate is frequently affected. Lesions begin with purplish erythema and progress to plaques and nodules that ulcerate easily. KS lesions may also involve the external genitalia including the foreskin and glans penis.

Diagnosis

Diagnosis of cutaneous KS is usually made based on clinical findings. However, in all inconclusive or questionable cases a histologic diagnosis is recommended. Differential diagnosis includes other neoplasia such as cutaneous lymphomas or angiosarcoma, but also infectious diseases such as syphilis and bacillary angiomatosis. Histological findings include spindle-shaped cells with vascular channels lined by abnormal endothelial cells.

In all cases of KS, clinical staging procedures are recommended, including:

- Complete inspection (oral and genital mucous membranes)
- Abdominal ultrasound
- Gastroduodenoscopy and colposcopy (both procedures obligatory when mucous membranes are involved)
- Chest radiography (exclusion of a pulmonary KS)

Treatment

If KS is newly diagnosed in an HIV-infected patient naïve to antiretroviral therapy, ART should be initiated: in early KS, additional chemotherapy is only required in 20% of adult cases. With viral suppression and immune reconstitution, many KS lesions stabilize or even resolve completely without specific treatment.In contrast, children with KS almost always need chemotherapy in addition to ART.Patients with KS should be referred to an oncologist to determine regimen and timing of chemotherapy.

Cervical intraepithelial neoplasia (CIN) and cervical cancer

In Sub-Saharan Africa, cervical cancer is the most common cancer in women aged below 35 years. Worldwide, however, cervical cancer is the second most common cancer in women. The incidence of some HIV-associated cancers, including Kaposi sarcoma and non-Hodgkin lymphoma, has fallen markedly in populations who have been treated with ART. In contrast, the incidence of cervical cancer has not changed significantly. Women with HIV infection are more likely to have infection with HPV 16 or 18 and to have a higher prevalence and incidence of CIN than HIV-negative women.

Diagnosis and management

Women with HIV and invasive cervical cancer should be managed in the same manner as HIV-negative women, according to specialized guidelines. Diagnosis is based on histopathological examination of cervical biopsies, and clinical staging. Regular screening allows for early diagnosis and treatment of cervical cancer.See National Guidelines for Cervical Cancer Screening for additional information.

Malignant lymphomas

Malignant lymphomas are neoplastic diseases of the lymphatic system that grow rapidly and aggressively, and lead to death within a few weeks or months if left untreated. Hodgkin's lymphoma (HL) is distinguished from the large group of non-Hodgkin's lymphomas (NHL). In comparison to the general population, HIV-infected patients are affected significantly more frequently by all types of lymphoma.Aggressive non-Hodgkin's lymphomas of B-cell origin are particularly frequent. The incidence of lymphomas has been markedly reduced by the introduction of ART.

Malignant lymphomas in HIV-infected patients are also biologically very heterogeneous and differ in several aspects. The extent of immunodeficiency also varies significantly. Burkitt's lymphoma and Hodgkin's lymphoma frequently occur even when immune status is good. In contrast, immunoblastic and primary CNS lymphoma (PCNSL) are almost always associated with severe immunodeficiency. It has also been noted that HIV-associated lymphomas – both NHL and HL – have numerous common clinical features. Characteristics include the usually aggressive growth, diagnosis in advanced stages with frequent extranodal manifestations, poorer response to treatment, high relapse rates and an overall poor prognosis.The treatment of such cases should follow the recommendations for HIV-negative patients in specialized centers.

Systemic non-Hodgkin lymphomas (NHL)

A close association between systemic NHL and AIDS has been described for a long time. More than 90% of HIV-associated NHLs are of B-cell origin. They are almost always of high-grade malignancy. Two main histological types dominate: Burkitt's lymphomas, which comprise 30–40% of cases, and diffuse large-cell B cell lymphomas, comprising 40–60%.

Prevention and early detection

There is no data supporting specific therapies or diagnostic procedures for prevention or early detection of malignant lymphomas. Antiretroviral therapy seems to be the best protection against lymphoma. ART not only improves the immune status but it also reduces chronic B-cell stimulation, a risk factor for the development of lymphoma. Viral suppression is important as cumulative HIV viremia is an independent and strong predictor of AIDS-related lymphoma among patients receiving ART.

Signs and symptoms

The main symptom is lymph node enlargement. Lymphomas are firm, immobile or barely mobile and painless. A large proportion of patients have advanced-stage lymphoma at the time of diagnosis. B symptoms with fever, night sweats and/or weight loss are found in the majority of cases (60–80%). General asthenia, significant malaise and rapid physical deterioration are also frequently seen.

Diagnosis

Rapid histological diagnosis is essential. If bone marrow biopsy cannot secure the diagnosis, then excision lymph node (e.g., cervical, axillary or inguinal) biopsy is recommended. All patients with suspected NHL should be staged. Basic diagnostic tests for staging include chest radiography; abdominal ultrasound; CT scans of the neck, thorax and abdomen; and bone marrow biopsy; aspiration alone is not enough. In addition to an updated immune status and viral load, the following should be determined at the very least: blood count, ESR, CRP, uric acid, LDH, liver and kidney parameters and electrolytes. ECG and echocardiography are also important right away.

Therapy

Due to extremely rapid generalization, even “early stages” move quickly. Every HIV-associated lymphoma is considered aggressive and requires systemic chemotherapy with a curative intent. Surgery or radiation therapy alone is not sufficient. Treatment should be started rapidly due to the aggressive nature of these lymphomas.

Special entities of lymphoma

Burkitt’s lymphomas: the particularly high proliferative capacity and aggressiveness of Burkitt’s lymphomas is a problem even in HIV-negative patients. Specific chemotherapy regimens are recommended.

Plasmablastic lymphomas: are relatively “new” entities in HIV-infected patients. Plasmablastic lymphomas probably belong to the diffuse large cell NHLs, but display a completely characteristic immune phenotype. The oral cavity is the site of involvement, although extra-oral manifestations do occur. Like Burkitt’s lymphoma, plasmablastic lymphomas have a very high rate of proliferation and are extremely aggressive. Prognosis remains poor.

Primary effusion lymphoma (PEL): a relatively rare entity, also called body cavity lymphoma. These lymphomas are often very difficult to diagnose histologically. A visible tumor mass is usually absent, and malignant cells can only be found in body cavities (e.g., pleural, pericardial, peritoneal). Every pleural or pericardial effusion occurring in an HIV-infected patient and containing malignant cells is suspicious of PEL. The involved pathologist should always be informed about this suspicion. Recent reports indicated encouraging results with a combined chemotherapy with high-dose methotrexate.

Primary CNS lymphoma

Primary CNS lymphomas (PCNSL) are a late complication of HIV-infection and were previously seen in up to 10% of AIDS patients. Histologically, findings are consistent with diffuse large cell non-Hodgkin’s lymphomas. CD4 is almost always below 50cells/mm^3 at the time of diagnosis. In the pre-HAART era, PCNSL had the poorest prognosis of all the AIDS-defining illnesses, with a median survival of less than three months. In more recent years, this bleak picture has changed significantly. In the ART era, survival may be several years and complete remission has become possible.

Signs and symptoms

Different neurological deficits occur depending on the location. Epileptic seizures may be the first manifestation of disease. Personality changes, changes in awareness, headaches and focal deficits such as paresis are also frequent. Fever is usually absent. As patients are almost always severely immunocompromised, constitutional symptoms may mask the real problem.

Diagnosis

Cranial CT or (better) MRI should be performed rapidly. The most important differential diagnosis is cerebral toxoplasmosis. A solitary mass is usually more indicative of PCNSL. However, 2–4 lesions may be present, which are usually fairly large at more than 2 cm in diameter. More than four lesions of a PCNSL are rarely found. In addition to the physical examination, a minimal diagnostic program (chest radiography, abdominal ultrasound) should clarify whether the CNS involvement is secondary to systemic lymphoma. This should always include fundoscopy to exclude ocular involvement (up to 20%).

Treatment

Cranial radiation therapy is only option for patients with PCNSL, independent of HIV status. All patients with PCNSL should be treated intensively with antiretroviral therapy, to achieve the best possible immune reconstitution.

Hodgkin's disease (HD)

The incidence of HD is elevated in HIV-infected patients by a factor of 5–15 compared to the HIV-negative population. Worrisome data indicate that the incidence of HIV-related HD is increasing in the setting of improved immunity. An advanced stage of disease at diagnosis is typical, as is frequent extranodal involvement and a trend towards prognostically poorer subtypes. Mediastinal disease is significantly less frequent than in HIV-negative patients.

Signs and symptoms

B symptoms occur in the majority of cases. Extranodal and advanced stages are almost always the rule. Lymphomas are firm, immobile or hardly mobile and painless, and the distinction from HIV-related lymphadenopathy or tuberculous lymphadenitis is not always possible.

Diagnosis

Staging is necessary as for non-Hodgkin lymphomas. Diagnostic lymph node excisional biopsy is even more important here than with NHL. As with NHL, specimens should be sent to reference laboratories if possible.

Treatment

Risk-adapted treatment strategy in patients with HIV-related HD in accordance with standard treatment procedures established for HIV-negative patients is recommended.

Non-AIDS-defining malignancies

HIV-infected patients have an increased risk of cancer. The risk for non-AIDS-defining malignancies is approximately two to three times higher in HIV-infected patients than in the non-infected population.

Anal carcinoma

Infections with human papilloma virus (HPV) are among the most frequently sexually transmitted virus infections. HIV-infected patients have a 2-to 6-fold higher risk for anal HPV infection, independent of gender and sexual practices.

Testicular tumors

Testicular tumors are the most frequently occurring cancer in men between 20 and 35 years of age. The relative risk factor for HIV-infected patients in the same age group is 2.5-fold. HIV-infected patients should be treated with the standard regimens that are also recommended for negative patients. Treatment should be performed in cooperation with a urologist experienced in oncology and an HIV specialist.

Lung cancer

In the general population, lung cancer is the most frequent cancer disease that leads to death in male patients. This tendency is increasing in women and already ranks third. The risk seems to be rising with HIV-infected patients. Overall risk seems to rise as immunodeficiency worsens.

CHAPTER8: NUTRITION AND HIV

Introduction

The 2013 Mid-Term Review of the Lesotho National HIV and AIDS Strategy revealed that food insecurity was increasingly becoming an important driver of the HIV epidemic. The link between HIV and nutrition is often described as a vicious cycle: both malnutrition and HIV weaken the immune system. HIV infection increases nutrient requirements and at the same time impairs nutrient intake and absorption. On the other hand, poor nutrition increases the risk of opportunistic infections and accelerates the progression of HIV and AIDS.

Good nutrition is an important component in the comprehensive care of people living with HIV.Additional intake enhances immune rehabilitation and adherence to ART.PLHIV will therefore benefit from referral to supplementary food programmes.

Macronutrients

The major cause of HIV related weight loss and wasting is a combination of low energy intake and increased energy demands as a result of HIV and related infections. Energy requirements increase by 10% to maintain body weight and physical activity in asymptomatic HIV infected adults and growth in asymptomatic children.

Symptomatic HIV infected adults have to increase their energy intake by 20 to 30% whereas children experiencing weight loss need an additional 50% to 100% energy intake on top of their normal requirements.

Micronutrients

PLHIV should consume diets that ensure micronutrient intake meets the recommended daily allowance (RDA) levels.Multivitamin and mineral supplementation should be considered for those at risk of vitamin or mineral deficiencies.

HIV-Infected Children

HIV-infected children should be routinely assessed for nutritional status, including weight and height at scheduled visits.Nutritional assessment and support should be an integral part of the care plan for any HIV-infected infant or child, irrespective of whether the child is on ART.Several anthropometric indices are used to assess nutritional status in children:weight for age (underweight), weight for height (wasting), and height for age (stunting).All indices are compared against a reference population of healthy children.

To define malnutrition in a clinical setting, wasting is a commonly-used indicator.It is defined by weight (kg) for height (cm) in standard deviations from the median (Z-score) or percentage of the median, as indicated below.In addition, mid-upper arm circumference (MUAC) may be used for assessment of nutritional status of infants and children 6-59 months of age (see below).A child who fallsinto moderare malnutrition should be enrolled on supplementary feeding (super cereal), while the one who falls into severe malnutrition should be enrolled on therapeutic feeding based on the following: if there are no complications give Plumpy nut ((RUTF), in case there are complications complications admit and give F75 or F100.See Annex 13 for reference values for weight for length/height Z-scores (to assess the degree of malnutrition) and Annex 14 for the dosing of RUTF in children.Refer to the National Guidelines for the Integrated Management of Acute Malnutrition for further details.

TABLE 8.1: Z-SCORE AND MUAC INTERPRETATIONS

Definition	Z Score Range	MUAC (cm)	Comment
Normal	Median to -1 SD		
Mild wasting	-1 to -2 SD<br>≤1 SD to ≤ 2 SD (Between -1SD and 2SD)	-	
Moderate wasting	≤-2 to ≤-3 SD (Between -2SD and -3SD)	11.5– 12.5	Supplementary feeding (CSB/supercereal)
Severe wasting	Below -3 SD	< 11.5	Inpatient if complications (loss of appetite, oedema)<br>If no complications give RUTF (plumpynut)

Infant Feeding and Maternal Nutrition in the context of HIV

The nutrition of children is critically important.Safe Infant feeding practices can reduce the likelihood of MTCT and the risk of infant death from malnutrition and other childhood infections.Furthermore, women have the right to full information to help them decide how to feed their children and to appropriate support.

Infant feeding counselling should begin during pregnancy to enable HIV-infected pregnant women to make informed infant feeding decisions.Every HIV infected mother should receive counselling which includes general information about the risks and benefits of the various infant feeding options and specific guidance on selecting the option most suitable for her particular situation including her health status and home environment.

Feeding for Infants ≤ 6 Months of Age

- **Exclusive breastfeeding is recommended for the first six months of life.**
- As life-long ARVs will be given to HIV-infected lactating women, the infant should be given daily Nevirapine (NVP) from birth until 6 weeks of age
- Replacement feeding (with commercial infant formula) should only be given to HIV exposed infant if **all** of the following conditions are met:
  - Safe water and sanitation can be assured at the household level and in the community
  - The mother or other caregiver can reliably provide sufficient infant formula milk to support normal growth and development of the infant
  - The mother or caregiver can prepare the milk cleanly and frequently enough so that it is safe and carries a low risk of diarrhoea and malnutrition
  - The mother or caregiver can exclusively give infant formula milk for the first six months
  - The family is supportive of the practice
  - The mother or caregiver can access health care that offers comprehensive child health services
- For details on how to assess whether the mother meets the above conditions please refer to the National PMTCT Guidelines.
- All mothers should be counselled on the management of breast conditions such as nipple cracks, fissures, etc., and on how to cope with breastfeeding difficulties.

HIV can be transmitted from the mother to the infant during breastfeeding.However, the risk can be reduced to a minimum by providing antiretrovirals to the mother and infant and by feeding the baby **exclusively** with breast milk for the first 6 months. **Exclusive breastfeeding means that nothing else (i.e. water, porridge, etc.) should be given to the infant.**Prescribed medications can be given.

Benefits of Exclusive Breastfeeding

- Breast milk provides complete nutrition for the infant for the first six months of life
- Colostrum, the milk produced during the first few days of the infant's life, is rich in vitamins and antibodies.It has other anti-infective properties as well.
- Breast milk contains antibodies from the mother which are beneficial to the infant as the infant's own immune system is not yet completely developed during the early months of life.
- Breast milk provides vital protection against deadly childhood illnesses such as diarrhoea and respiratory infections.
- Breast milk is easily digested and its composition changes to meet the developmental needs of the growing infant.
- Breast milk contains enzymes that help in the digestion of fat.
- Breast milk is natural and does not add extra costs.
- Breastfeeding promotes bonding between mothers and their babies.
- Breastfeeding helps the uterus to contract after delivery and reduce the risk of post-partum haemorrhage.
- Breast milk is always available and no special preparation is needed.

Challenges of Exclusive Breastfeeding

- Exclusive breastfeeding can be difficult, particularly for mothers working away from home
- It may be difficult to withstand family or community pressure to give other liquids or foods
- The mother requires additional calories to support breastfeeding

Benefits of Replacement Feeding with Commercial Infant Formula

- There is no risk of HIV transmission to the baby
- Commercial infant formula contains most of the nutrients an infant needs
- Other people (besides the mother) can feed infant

Challenges of Replacement Feeding with Commercial Infant Formula

- The infant does not benefit from the protective effects of colostrum
- Infant formula does not contain the antibodies found in breast milk
- There is an increased risk of diarrhoeal illnesses and respiratory infections with an increased risk of infant mortality, particularly in the first 6 months of life.
- There is an increased risk of malnutrition due to inadequate supply of infant formula or inappropriate feeding
- Commercial infant formula is expensive (and a regular supply must be assured)
- Requires a regular supply of fuel and clean water for preparation
- Infant formula cannot be stored; it must be freshlyprepared each time it is needed

Although exclusive breastfeeding is recommended for the first six months of life for infants born to HIV-infected mothers, each mother should be informed of all available options and taken through an assessment of her individual circumstances to identify her best infant feeding option. A mother should be supported whatever choice she makes, with emphasis on the importance of exclusively practicing the option taken.

Mothers should be warned against mixed feeding (breast milk plus other foods or liquids) during the first six months of life as this is associated with a **higher risk** of HIV transmission than breastfeeding exclusively.

Infant Feeding from Six to Twelve Months of Age

- HIV infected mothers (and those whose infants are HIV uninfected or with unknown HIV status) should continue breastfeeding for the**first 12 months of life,** while antiretrovirals are provided to the mother for her own health and for reducing the risk of MTCT of HIV.
- After six months, all infants should start receiving complementary foods in addition to breast milk (or replacement milk). This is a high-risk time for all infants as it is often associated with growth faltering, illness, and increased risk of malnutrition. HIV exposed infants must be monitored closely since they are at an increased risk of these complications. HIV-infected mothers should therefore receive regular support and counselling for appropriate complementary feeding.
- Health care providers should promote and encourage responsive (active) feeding, applying the principles of psychosocial care as well as supporting the maintenance of food safety and hygiene to avoid food borne diseases. Food demonstrations should be used to introduce mothers to safe and nutritious meals for their infants. Guidance should focus on the quantity, quality and frequency of feeding.

Infant Feeding from Twelve to Twenty-Four Months

- After twelve months of age, breastfeeding for all infants **should only** stop if a nutritionally adequate and safe diet can be provided to the infant.
    - Appropriate complementary foods should still be provided during this time.
- Studies indicate improved survival among HIV infected infants who are breastfed.All HIV-infected infants should therefore be breastfed exclusively for the first six months and continue breastfeeding for as long as possible while receiving complementary foods.

When Mothers Decide to Stop Breastfeeding

- When HIV-infected mothers stop breastfeeding, they should do so gradually within one month as stopping breastfeeding abruptly is not advisable.
- If the infant is younger than 6 months of age when breastfeeding ceases:
    - Commercial infant formula milk should be provided exclusively
    - Note that modified animal milk is not recommended for children under 6 months.
- If the infant is older than 6 months when breastfeeding ceases, feeding options include:
    - Commercial infant feeding formula milk
    - Animal milk, which should be boiled for infants under 12 months

Interim Feeding Strategies

- HIV infected mothers may consider expressing and heat-treating breast milk as an interim feeding strategy under the following conditions:
    - In the neonatal period if the infant is born with low birth weight or is otherwise ill

    - and unable to breastfeed
    - The mother is unwell and temporarily unable to breastfeed or has a temporary breast health problem such as mastitisor
    - Antiretroviral drugs are temporarily not available
- Some mothers may also consider using expressed and heat-treated breast milk as a step towards stopping breastfeeding if the baby is younger than six months of age.
- Mothers choosing to utilize these interim feeding strategies should receive appropriate instructions from health care providers describing the appropriate procedure for heat-treating breast milk in order to ensure that the milk retains its full nutritional benefits.

Maternal Nutritional Support

Good nutrition for pregnant and breastfeeding mothers is important for the survival and well being of the developing baby.In addition, an HIV positive mother's nutrition before, during and after pregnancy can influence her own health and the risk of transmitting HIV to her child. HIV positive mothers are at a higher risk of malnutrition and illness while pregnant and breastfeeding.During pregnancy or lactation, the mothers' need for energy and other nutrients increases to meet the demands of:

- Adequate weight gain due to pregnancy
- Development of the baby
- Milk production

In order to maintain good health, HIV positive mothers therefore need additional food to meet the extra demands associated with HIV, pregnancy and lactation.Food intake for pregnant women includes a variety of macro and micronutrients.For further management of diet and related conditions refer to the Lesotho Food Based Dietary Guidelines for people living HIV and AIDS.

HIV-Infected Adults

Nutritional assessment and management is a central component to the comprehensive care of PLWHIV. Numerous studies have shown the association between indices of nutritional states such as body mass index (BMI) and mortality.Body mass index is the main indicator for malnutrition in adults.Defined as body weight in kilograms divided by the height in meters squared [Weight (kg)/ Height (m^2)], BMI interpretations are as follows:

TABLE 8.2: BMI INTERPRETATIONS

BMI Range	Interpretation
18.5 – 25.00	Normal
17.0 – 18.49	Mildly Underweight
16.0 – 16.99	Moderately Underweight
< 16.0	Severely Underweight

When BMI below 18.5 is diagnosed, therapeutic food supplementation is recommended*(refer to the National Guidelines for the Integrated Management of Acute Malnutrition)*

TABLE 8.3: LIST OF MACRONUTRIENTS

Nutrient	Sources	Functions
Protein	▪ Meat-chicken, pork, beef, fish<br>▪ Dairy-milk, yoghurt, cheese<br>▪ Egg<br>▪ Nuts/grains-peanuts, bread<br>▪ Legumes-beans	▪ Provide necessary materials for building and repairing worn-out tissues<br>▪ Develops the immune systems and resistance to infections
Carbohydrates	▪ Vegetables	▪ Provide energy for the body

	▪ Papa, samp, potatoes<br>▪ Fruits - peaches, bananas, apples<br>▪ Grains - bread, rice, cereal	▪ Fibre (a non-digested type of carbohydrate found in grains, fruits and green vegetables) prevents constipation, coronary heart disease and diabetes<br>▪ Soluble fibre is used in diarrhoea treatment
Fats	▪ Cooking oil, butter and animal fats	▪ Provide energy and heat; important for weight gain<br>▪ Aid in the absorption of and transportation of fat-soluble vitamins

TABLE 8.4: LIST OF MICRONUTRIENTS

Nutrient	Source	Function
Vitamin A	Carrots, spinach, pumpkin, peaches, tenane, sepaile,milk eggs,liver,fish,oils	▪ Good for white blood cells, vision and bone development<br>▪ Anti-oxidants needed for immune function and resistance to infections
VitaminB1 (thiamine)	Milk, eggs, beans, liver, fish, Likhobe tsa poone,tsa mabele, tsa koro, pork	▪ Used in energy production<br>▪ Supports heart, muscles, and central nervous system
Vitamin B2 (riboflavin)	Milk, eggs, beans, nuts, dairy, nama ea khoho, fish, likhobe	▪ Energy production, good vision, making blood cells
Vitamin B3 (niacin)	Milk, eggs, red meat, poultry, peanuts, likhobe	▪ Energy production, healthy skin, supports the nervous system
Vitamin B6	Likhobe, potatoes, bananas, beans, poultry, greenvegetables, tomatoes, liver, fish, watermelon	▪ Breakdown protein and fat, production of antibodies<br>▪ Assists in production of red blood cells and supports function
Vitamin B12	Fish, liver, poultry, kidneys, sardines, milk, cheese, yoghurt, eggs	▪ Formation of red blood cells<br>▪ Maintains nerve and digestive tissues
Vitamin C (ascorbic acid)	Oranges, tenane, lshoabe, theepe, spinach, tomatoes, bell peppers, apples	▪ For healthy teeth, gums and bones<br>▪ Fights infection<br>▪ Helps iron absorption<br>▪ An anti-oxidant
Vitamin E	Sunflower oil, likhobe, beans, peas, lentils, cabbage, tenane, leshoabe, eggs	▪ An anti-oxidant that helps prevent cells from damage, increase disease resistance, and aids healing of scar tissue
Folate (folic acid)	Poultry, liver fish, beans, peas, green leavy vegetables, oranges	▪ Builds new cells, especially red blood cells
Calcium	Milk, mafi, yoghurt, spinach, cabbage, sepaile,beans, peas, lentils	▪ Builds strong bones and teeth<br>▪ Necessary for normal muscle function and blood clotting
Iodine	Fish,iodized salt, meroho ea Sesotho(e.g. theepe, tenane, leshoabe, seruoe)	▪ Development and proper thyroid function<br>▪ Important for normal growth and development, and prevent goiter
Zinc	Theepe, sepaile, pumpkin, likhobe, nuts, beans,corn, milk, cheese, liver, eggs, garlic, poultry, fish, red meat	▪ Important for growth and development<br>▪ Supports the immune system and improves wound healing
Selenium	Fish, red meat, likhobe, eggs, rice, sepaile	▪ An anti-oxidant<br>▪ Helps prevent breakdown of cells
Magnesium	Beans, peas, lentils, likhobe, spinach, sepaile	▪ Supports muscle and nerve function<br>▪ Releases energy from fats, proteins and carbohydrates<br>▪ Build strong bones and teeth
Iron	Red meat, pork, liver, eggs, green leafy vegetables, beans, peas, lentils, mangangajane	▪ Needed for the production of red blood cells and the delivery of oxygen to body tissues

CHAPTER9: WELLNESS INFORMATION

Introduction

Many people living with HIV and AIDS need counselling and support in order to learn how to care for themselves and lead healthy, positive lives.A healthy lifestyle can help to slow disease progression and promote safer sexual practices, which in turn will reduce the risk of transmitting the virus to others.Some aspects of a 'Wellness' Programme include:

9.1 Healthy diet

- PLHIV should eat healthy foods
- Eat a balanced diet, which includes many fresh fruits and vegetables

9.2 Consider nutritional supplements

- The use of nutritional supplements can be of value if the patient is unable to eat a balanced diet.

9.3 Avoid smoking

- Tobacco smoke (first or second-hand) harms lung immunity
- Since respiratory infections account fora large proportion of opportunistic infections, a healthy respiratory system is important.
- Patients shouldbe encouraged and assisted to stop smoking.

9.4 Avoid alcohol intoxication

- Too much alcohol is harmful to one's health
- Since many drugs used in HIV disease are potentially toxic to the liver, a healthy liver is important.
- Advise patients to minimize alcohol intake, which among other things, will have a negative effect on adherence to ARVs and other important medications.

9.5 Keep fit and well exercised

- Exercise helps to keep the body in good physical shape and will help patients to feel well and strong.
- However, advise patients not to over-stress the body, especially when symptoms of disease are present (diarrhoea, cough, fever, etc).

9.6 Avoid taking unnecessary drugs

- Any drug has potential side effects.
- The potential risk of medication must always be weighed against the potential benefit.
- Patients should only take medication which has been prescribed by a trained health care provider.

9.7 Get a lot of rest and sleep

- Rest regularly and get enough sleep.
- If at all possible, patients should avoid too much stress.

9.8 Have a positive mental attitude

- A positive mental attitude promotes well-being, and helps to keep patients well for longer.

9.9 Alternative therapies

- Alternative therapies such as acupuncture, massage, homeopathy, herbal medicine and traditional healing may be of some benefit, but their value has yet to be proven in HIV/AIDS care.
- They can be considered as supportive therapy and should not be discouraged in patients with strong beliefs in such therapy.

9.10 Seek treatment early for medical problems

- It is important to seek treatment for medical problems as soon as symptoms appear.
- Many HIV-related conditions can be effectively treated if they are diagnosed early enough.
- Encourage patients to come for assessment as soon as they notice any problems.

9.11 Safer sexual practices

- It is important for patients to prevent spreading HIV infection to others.
- It is also considered harmful to get repeated HIV infection from others.
- Condoms are recommended for all sexual encounters.

9.12 Illegal Drugs (including marijuana)

- These should be discouraged as they can have a negative impact on adherence and influence people to make unwise and unsafe decisions.

9.13 Advice on vaccines

- All HIV-positive people are advised to have an annual Influenza vaccine.
- Hepatitis B immunization may be given if the person has not already been infected with Hepatitis B.
- Live vaccines should be avoided in those with weakened immune systems (particularly if CD4 < 200). The effectiveness of vaccines in HIV+ patients is higher when CD4 cell count is > $200/mm^3$.

CHAPTER10: POST EXPOSURE PROPHYLAXIS (PEP)

SECTION 10.1: INTRODUCTION

Definition: The term *post-exposure prophylaxis* is generally understood to mean the medical responsegiven to prevent the transmission of blood-borne pathogens following a potential exposure toHIV.In the context of HIV, post-exposure prophylaxis refers to the set of services that areprovided to manage the specific aspects of exposure to HIV and to help prevent HIV infection.

The services include first aid; counselling including the assessment of risk of infection from the exposure; HIV testing; anddepending on the outcome of the exposure assessment; a prescription for a 28-day course of ARVs with appropriate support and follow-up.

People exposed to HIV and other pathogens through sexual assault or occupational exposure merit close monitoring.In addition to the risk of infection, these experiences are psychologically devastating.For these reasons, avoidance of occupational exposure and proper management of sexual assault victims should be given top priority.

It is imperative that HIV post-exposureprophylaxis policies reinforce the importance of primary risk preventioninall settings where HIV could be transmitted. PEP should never be provided in isolation butshould form part of a wider strategy for preventing exposure to HIV.It should also be associatedwith measures to prevent other bloodborne diseases such as Hepatitis B and C.

Non-DiscriminativeProvision of PEP

The policy for PEP eligibility should be founded on the principle of equity.Decisionsabout whether or not to offer post-exposure prophylaxis should be based purely on clinicalconsiderations of risk and should not be tied in any way to a person’s decision to file a policereport or to pursue legal action.

Individuals should be assessed for PEP regardless of theirinvolvement in any activities considered to be illegal by national legislation such as injecting drug use; sex work; or men having sex with men.Nor should there be any barriers to access PEP forfinancial or administrative reasons.

SECTION 10.2: INDICATIONS FOR PEP

PEP is aimed at preventing HIV from invading sites such as the lymph nodes and testis. Within the first 72 hours of HIV exposure, these sites are thought to be invaded by the virus, which remains there permanently. Therefore, administering HIV PEP within 72 hours of exposure to HIV is the most effective way of preventing HIV infection and should NOT be initiated beyond 72 hours after the event.

Occupational Exposure Incidents

Management of occupational Exposure

- Cleanse the wound thoroughly with soap and water. Do not use antiseptics or squeezethe injured site
- Report the incident to the supervisor
- Document the incident
- Evaluate the exposure incident
- Evaluate the exposure source

When available, obtain the **HIV status and Hepatitis B status**of the exposure source. If the source is HIV-infected and information is available, determine:

a. Stage of disease
b. Current and previous antiretroviral therapy
c. Viral load
d. Antiretroviral resistance information

It is also important to know the HIV status and Hepatitis B status of the exposed person.A complete history should be taken to identify other medical conditions(renal disease, liver disease, diabetes, or mental health diagnosis), drug allergies, and pregnancy or breast-feeding status.

Non healthcare related Exposures

- Sexual Assault
- Police service men
- Crime scene attendants
- Accident attendants
- Unprotected consensual sexual intercourse
- Condom breakage

SECTION 10.3: PROVIDING PEP

TABLE 10.1: RECOMMENDATIONS FOR PEP

Exposure	HIV status of source patient		
	Unknown	***Positive***	***High Risk***
Intact Skin	No PEP	No PEP	No PEP
Mucosal splash/non intact skin	Consider 2-drug regimen	Recommend 2-drug regimen	Recommend 2-drug regimen
Percutaneous (sharps)	Recommend 2-drug regimen	Recommend 2-drug regimen	Recommend 3-drug regimen
Percutaneous (needle in vessel or deep injury)	Recommend 2-drug regimen	Recommend 3-drug regimen	Recommend 3-drug regimen

TABLE 10.2: RECOMMENDED PEP DRUG REGIMEN

Drug	Dose	Frequency	Duration
Zidovudine (AZT)<br>Lamivudine	300mg<br>150mg	12hourly<br>12hourly	28days
Lopinavir/ritonavir (3rd drug)<br>In cases of high exposure	400mg/100mg	12hourly	28days

STEP 1: REPORT AND DOCUMENT

Document all HIV exposures in the PEP register, incident report book and exposed person's bukana.

Confidentiality - PEP evaluations for both the exposed and source individual (if known) should be treated with confidentiality.

Circumstances of the exposure and PEP management should be recorded in the exposed person's incident report. Details should include:

- Date and time of exposure

- Details of the incident: where and how the exposure occurred, exposure site(s) on body
- Details of the exposure: type and amount of fluid or material, severity of exposure

STEP 2: EVALUATE THE EXPOSURE

The exposure should be evaluated for potential to transmit HIV based on the type of body substance involved, the route, and HIV status of the source patient.

FIGURE 10.1: ALGORITHM FOR EVALUATION AND TREATMENT OF POSSIBLE NON OCCUPATIONAL HIV EXPOSURES

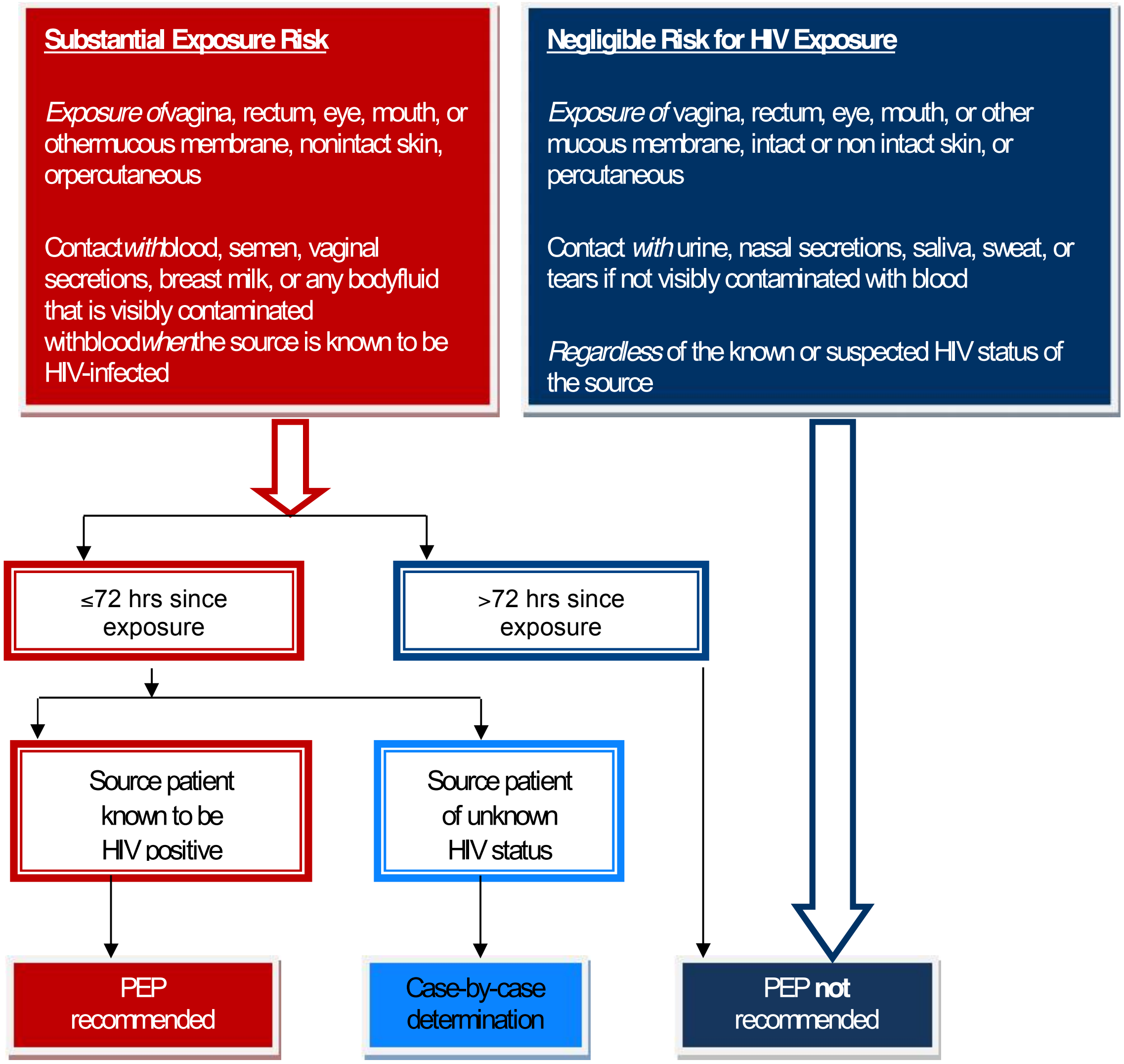

STEP 3: EVALUATE THE EXPOSURE SOURCE

Regardless of HIV status, assess and assist with access to medical care, social support services, and risk reduction counseling.

TABLE 10.3: RELEVANT HISTORY

Known HIV infection	▪ Obtain history of antiretroviral medications, recent viral load,CD4 cell count and date of results<br>▪ Consider drawing HIV viral load, CD4 cell count and resistance testing<br>▪ Consider evaluation and testing for other sexually transmitted infections, including Hepatitis B and Hepatitis C
Unknown HIV infection	▪ Obtain risk history and rapid HIV test<br>▪ Consider evaluation and testing for other sexually transmitted infections, including Hepatitis B

STEP 4: MANAGEMENT OF POTENTIAL EXPOSURES

1. Baseline Evaluation

For all exposed people, the following baseline tests are recommended:

- HIV testing
- Serologic testing for Hepatitis B
- Pregnancy testing, as appropriate

Exposures from sexual assault, consensual sex, condom breakage also need:

- Screening and prophylaxis for other sexually transmitted infections
- Assess need for emergency hormonal contraception

Note:If consultation is not immediately available, PEP should not be delayed; changes can be made as needed after PEP has been started. If the source patient is found to be HIV uninfected, PEP should be discontinued.If the exposed person found to be HIV infected, eligibility to initiate ART should be assessed.

- Exposed persons are frequently unable to complete PEP regimens due to side effects. Providing prophylactic symptom management can improve adherence.
- A starter pack of 3days may be prescribed initially with a scheduled follow-up appointment for HIV test, evaluation for potential medication side effects, and continue counseling on risk reduction, support and adherence.

2. Counseling after HIV exposure:

- Advise an exposed person to refrain from donating blood and utilize risk reduction methods including use of condoms during sex, not sharing injection equipment, and/or abstaining from high risk behaviors.
- Offer mental health counseling as needed.
- Counsel the exposed individual about the signs and symptoms of acute retroviral syndrome (flu-like syndrome), and the need to come in for additional testing should these symptoms develop.

SPECIAL CONSIDERATIONS

1. Pregnancy risk
   - o Provide emergency contraception
   - o Bear in mind drug interactions
2. Known or suspected pregnancy
   - o Does not preclude the use of optimal PEP regimens
   - o Do not deny PEP on the basis of pregnancy.
3. Children
   - o PEP should be offered, usingpaediatric dosing of ARVs.
   - o Offer psychosocial support and counselling for assault victims.

- Provide prophylaxis or treatment for STIs as needed
    - Offer emergency contraception for all pubertal girls – Tanner Stage 3 and above
4. Sexual assault survivors
    - Provide prophylaxis or treatment for STIs.
    - Offer emergency contraception for any woman who may become pregnant
    - Offer psychosocial support and counselling
    - **Do not deny or delay PEP if a patient does not have a Sexual Assault Form (police form) or intention to pursue legal action.**
5. Injection-drug users/sex workers
    - PEP should not be refused for high risk incidents and risk reduction strategies should be discussed.

STEP 5: FOLLOW-UP

TABLE 10.4: HIV FOLLOW UP TESTING

Test	Baseline	6 Weeks	3 Months	6 Months
HIV Testing	X	X	X	X
Pregnancy	X	X		
Hepatitis B	X			X

Resistance of the source virus to antiretroviral agents

- If the source person's virus is known or suspected to be resistant to one or more of the drugs considered for the standard PEP regimen, alternate drugs are to be selected in consultation with an HIV Expert.
- If a person with a drug-resistant strain of HIV subsequently infects someone else with HIV, drug resistance spreads within the community.

Toxicity of the initial PEP regimen

- Adverse symptoms such as nausea, diarrhea, and headaches are common with PEP.
- Symptoms can often be managed without changing the PEP regimen by prescribing analgesic, antidiarrhoea and/or antiemetic agents.

CHAPTER 11: INFECTION CONTROL

An effective infection control programme includes several components that work to prevent healthcare providers and patients from suffering needle stick and other sharps-related injuries.Avoidance of occupational exposure should be given top priority.Each health facility in Lesotho should have an Infection Control focal person and a plan. Measures to be undertaken include the use of gloves (i.e. "Universal precautions") in all settings and proper disposal of containers for sharps and other contaminated materials.

A model of quality improvement for a prevention programme and operational processes should foster a culture of safety, reporting injuries and accessing care and treatment.Surveys of healthcare providers indicate that 50% or more do not report their occupational percutaneous injuries.

Universal Precautions are designed to prevent transmission of human immunodeficiency virus (HIV), hepatitis B virus (HBV) and other blood borne pathogens when providing health care.Under universal precautions, blood and certain body fluids of all patients are considered potentially infectious for HIV, HBV and other blood-borne pathogens.

Universal precautions involve the use of protective barriers such as gloves; gowns; aprons; masks or protective eyewear; which reduce the risk of exposure to potentially infective materials.Universal precautions are applied in a two-way fashion: healthcare provider to patient and patient to healthcare provider:

- Hand-washing before and after patient contact
- Decontaminate equipment and devices
- Use and disposal of needles and sharps safely (avoid recapping, especially two-handed)
- Wear protective gear
- Promptly clean up blood and body fluid spills
- Use safe disposal systems for waste collection and disposal

All healthcare providers should routinely take appropriate barrier precautions to prevent skin and mucous membrane exposure during contact with any patient's blood or body fluids.

Hand Hygiene

Hand-washing is the **single most important measure** to reduce the risks of transmitting microorganisms from one person to another.Washing hands as promptly and thoroughly as possible between patient contacts and after contact with potentially infectious material is an important component of infection control.In addition to hand-washing, gloves play an important role in reducing transmission of microorganisms.

- Use soap and water for hand washing under running water for at least 15 seconds.
- Use alcohol-based hand rubs (or antimicrobial soap) and water for routine decontamination

Basic Personal Protective Equipment

- Gloves of correct size
- Apronsas waterproof barriers
- Eyewearto avoid accidental splashes
- Footwear such as rubber boots or clean leather shoes

Gloves should be worn:

- When touching blood and body fluids, mucous membranes or non-intact skin of all

patients.
- When handling items or surfaces soiled with blood or other body fluids.

Change gloves after contact with each patient.Wash hands and other skin surfaces immediately if contaminated with blood or other body fluids.Immediate hand washing is also recommended on removal of gloves.Routine use of gloves should reduce the incidence of blood contamination of hands during phlebotomy but cannot protect against penetrating injuries caused by needles or other sharp instruments.Gloves should never be washed for reuse.Use of gloves is obligatory when the health care provider has cuts, scratches, or other breaks in his/her skin.Performing finger and/or heel sticks on children requires gloves.

Airborne Precautions

Airborne precautions are designed to reduce the risk of airborne transmission of infectious agents.Airborne transmission occurs by dissemination of nuclei of evaporated droplets that may remain suspended in the air for long periods of time.N95 masks (N category at 95% efficiency) should be used by health care providers in situations where exposure to TB is probable.TB infection control has 3 components; listed in their order of importance:

Administrative controls

- Prompt identification of infectious TB cases
- Physical isolation of patients known or suspected to have TB
- Coughing patients should be separated from other outpatients in waiting areas
- Physical separation of TB suspects from HIV-infected people (patients and staff) is especially important
- Reduce the length of admission if possible, to prevent nosocomial infection

Note: These controls are most effective but least expensive.

Environmental (or engineering) controls

These are important in triage rooms in OPD and rooms where TB patients (suspected or confirmed) have been admitted.

- Natural ventilation, whichcan be as simple as opening windows.
- Mechanical ventilation such as using extraction fans.
- Ultraviolet irradiation
- Air filtration

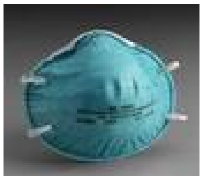
N 95 Respirator Mask

Personal respiratory protection

- Use of N95 or any other respirator masks
- The masks must be properly fitted in order to protect against

Note that surgical masks **donot** protect against TB.

Handling and Disposal of Sharps

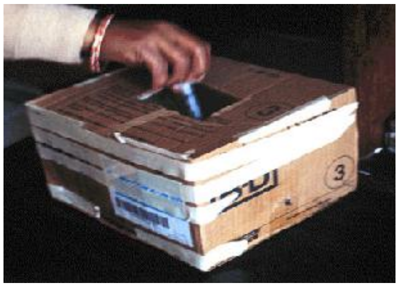

- Use syringe and needle once only.
- Do not recap the needle after use.
- Do not bend or break needles.
- Use puncture-proof containers for disposal.
- Clearly label container: “SHARPS”.
- Never overfill or reuse sharps containers.

- Dispose of sharps according to hospital guidelines.
- Use a puncture-proof container for storage and/or disposal.
- Do not recap a needle before disposal nor use the one-hand technique; it is high risk behaviour. Use needle removers which remove the needle from the syringe by cutting the hub of the syringe and/or the needle.
- Use auto-disable syringes or automatically retractable syringes: The advantage is that they cannot be re-used and they save time for healthcare providers from the burden of sterilization.

Sterilization and disinfection of medical devices

In general, medical devices or equipment for patient use that enters sterile tissue or the vascular system or through which blood flows should be sterilized before each use.Sterilization means the use of a physical or chemical procedure to destroy all microbial life, including highly resistant bacterial endospores.

Disinfection means the use of a chemical procedure that eliminates virtually all recognized pathogenic microorganisms but not necessarily all microbial forms (i.e. bacterial endospores) on inanimate objects.

There are three levels of disinfection: high, intermediate and low.High-level disinfection kills all organisms, except high levels of bacterial spores.It is effected with a chemical germicide cleared for marketing as a sterilant.Intermediate disinfection kills mycobacterium, most viruses, and bacteria with a chemical germicide (Sidex).Low-level disinfection kills some viruses and bacteria with a chemical germicide registered as a hospital disinfectant.**Gloves should always be worn during the sterilization process.**

Apply risk reduction strategies:

- Assess condition of protective equipment
- Safely dispose waste materials
- Avail appropriate cleaning and disinfecting agents
- Decontaminate instruments and equipments
- Monitor skin integrity

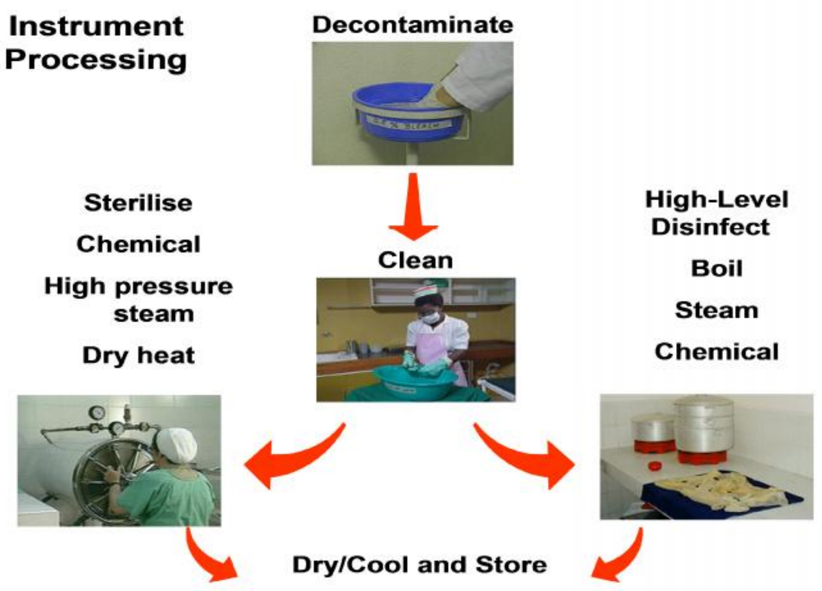

Ongoing Education for health care providers in infection prevention is essential to make all staff aware of established infection control policies.

Management of Occupational Exposure

- Provide immediate care to the exposed site
- Evaluate the exposure
- Give post exposure prophylaxis (PEP) for exposures posing risk of infection transmission.
- Perform follow up testing and counselling

Favero MS, Bond WW.Sterilization, disinfection, and antisepsis in the hospital. In: Manual of Clinical Microbiology, 1991; chapter 24:183-200. American Society for Microbiology.Washington, DC; RutalaWA.APIC guideline for selection and use of disinfectants. Am J Infect Control 1996;24:313-342.

Glossary

Blood-borne pathogens: Pathogenic microorganisms that are present in human blood and can cause disease in humans.These pathogens include but are not limited to hepatitis B virus (HBV), hepatitis C virus (HCV) and human immunodeficiency virus (HIV).

Occupational Exposure: Means reasonably anticipated skin, eye, mucous membrane, or parenteral contact with blood or other potentially infectious materials that may result from the performance of a health care provider's duties.

Percutaneous: Effected or performed through the skin.

Phlebotomy: The sampling of blood for transfusion, pheresis, diagnostic testing or experimental procedures.

Recapping: The act of replacing a protective cap on a needle

Seroconversion: The development of antibodies in the blood of an individual following exposure to an infectious agent.

Sharps Injury: An exposure that occurs when any sharp medical instrument penetrates the skin

Standard Precautions: An approach to infection control recommended by the centres for disease Control and Prevention since 1996.Standard precautions synthesize the major features of universal precautions and apply to blood and all moist body substances, not just those associated with blood borne virus transmission.Standard precautions are designed to prevent transmission of infectious agents in the health care.

Universal Precautions: Designed to prevent transmission of human immunodeficiency virus (HIV), hepatitis B virus (HBV), and other blood borne pathogens when providing health care.Under universal precautions, blood and certain body fluids of all patients are considered potentially infectious for HIV, HBV and other blood borne pathogens. (www.cdc.gov)

Note that Universal Precautions do not apply to faeces, nasal secretions, sputum, sweat, tears, urine, saliva and vomitus unless they contain visible blood.

CHAPTER 12: OPERATIONAL AND SERVICE DELIVERY

This chapter provides guidance on some key operational and service delivery issues that need strengthening to ensure comprehensive delivery of HIV and AIDS prevention, care and treatment services. The chapter covers the following areas: retention across the decentralization of HIV treatment and care, retention across the continuum of care, service integration and linkages (ART in TB treatment settings and TB treatment in HIV care settings, ART in MNCH), human resources, laboratory and diagnostic services and procurement and supply management system.

SECTION 12.1 DECENTRALIZATION OF HIV CARE AND TREATMENT

Decentralization can be defined as the transfer of authority and technique or dispersal of power, in public planning, management and decision making from the national level to the sub-national levels or more generally from higher to lower government levels in a country[1]. With approximately 60% of people in Lesotho residing in rural areas; the country adopted decentralization as a public health approach to improve access to ART and increase the health and survival of PLHIV. Decentralization also helps to decongest hospitals so that clinicians can focus on managing more serious health conditions.

Possible benefits of decentralization include[2]:

- Integrated health service delivery at lower levels, particularly for primary health care
- Integration of public and private entities and improved inter-sectoral coordination
- Reduction in inequalities and promotion of equity between different geographic settings and between urban and rural areas.
- Cost containment and reduction in duplication of services at secondary level of health care delivery
- Greater community involvement in management of their own health thus leading to more appropriate health plans in relation to local health needs and problems
- Greater community ownership, participation and willingness to contribute to financing of their health needs at local level
- Overcoming problems and delays due to factors such as long distances, inadequate communication and poor road networks/terrain
- Some models of decentralization such as Community ART Groups (CAGs) demonstrate increased levels of retention in care

ART initiation and maintenance of patients on ART should be provided at hospitals and health centres by trained nurses. Patients no longer have to travel long distances to access ART services unless faced with complications that may require specialized medical services at hospital level. HIV testing and counselling services should be offered at all health facilities and in the communities through trained community-based cadres including trained lay counsellors and village health workers.

Patients who are stable on ART can receive follow up care/maintenance at community level by trained community-based cadres between regular clinical visits at health centres. The follow up care package may include adherence counselling and support, supply of pre-packed ARVs, simplified clinical assessments and recording patient information.

---

[1] RONDINELLI, D.A (1981).Government decentralization in comparative theory and practice in developing countries.International review of administrative sciences, 47 (2): 133- 145

[2] Zimbabwe Guidelines for the Decentralization of HIV and AIDS Care and Treatment Services, 2009, MOHCW

SECTION 12.2: RETENTION ACROSS THE CONTINUUM OF CARE

The cascade of HIV care and treatment includes: HIV testing, linkage to and retention in longitudinal care, initiation of antiretroviral treatment (ART), adherence to antiretroviral medications, and, lastly, viral load suppression, which results directly in improved health status and reduced risk of HIV transmission. With high attrition rates along the HIV care and treatment cascade; the result is low overall coverage and impact of the final critical steps of the cascade. Literature from sub-Saharan Africa has shown that 54% of those who are not yet eligible for ART were lost to follow up before becoming eligible, while 32% of the PLHIV who were eligible for ART were lost before initiating treatment[3].

FIGURE 12.1 FACTORS AFFECTING RETENTION AND POSSIBLE INTERVENTIONS

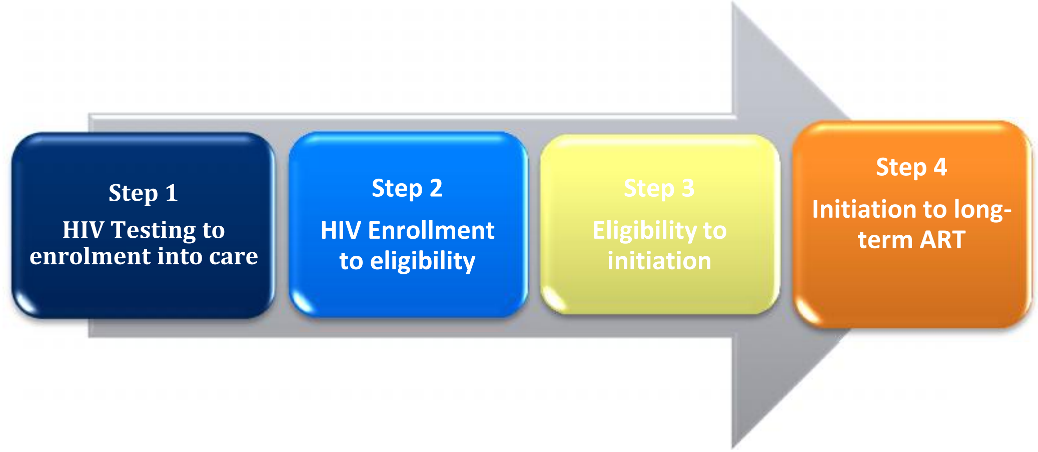

Multiple interventions at different levels of care are required to optimize patient retention. It is important to identify specific barriers to retention and address them. Several factors may impact patients accessing services and affecting retention in care such as costs associated with accessing services, stock-outs of ARVs, lack of effective referral systems, lack of monitoring system, comorbidities, and forgetfulness.

TABLE 12.1- FACTORS AFFECTING RETENTION AND POSSIBLE INTERVENTIONS

Factors affecting retention	Possible Interventions
**High costs of receiving care**	Continue to decentralize ART services<br>Provide family-centred care<br>Reinforce the appointment system for patients
**Weak systems for monitoring patient retention**	Implement patient tracking systems including cohort monitoring and electronic patient monitoring systems
**Weak patient referral system**	Strengthen patient referral system including use of patient referral forms<br>Use of unique patient identifiers to track patients across different points of care
**Adherence support**	Using community health workers to provide adherence support<br>Peer support
**Forgetfulness**	Linking to support groups<br>Using text message reminders

[3]Fox MP, Rosen S. Patient retention in antiretroviral therapy prorammes up to three years on treatment in sub-Saharan Africa, 2007-2009: systematic review. *Tropical Medicine and International Health*, 2010, 15(Suppl.1):1-16.

SECTION 12.3: SERVICE INTEGRATION AND LINKAGES

Programmes of HIV and AIDS, SRH, maternal and child health, and TB need to collaborate so as to minimize missed opportunities while at the same time provide more comprehensive health care to patients. Areas of collaboration may include training, supervision and mentorship, supply chain management, resource mobilization and monitoring and evaluation.

Delivering ART in ANC and MNCH settings

Studies have shown that provision of ART within ANC and MNCH settings increases the uptake of ART among HIV infected pregnant women. As many women access maternity services during pregnancy; this serves as an opportunity to also provide HIV testing and counseling services to all pregnant women and offering life- long ART for those found to be HIV infected. Nurses and midwives should be trained, mentored, and supervised to provide ART initiation and follow up care within the MNCH settings and refer mother-baby pair at 24 months to ART clinic for follow up care. Where possible and depending to clinic setup partners of HIV infected pregnant and lactating women should also be offered PITC and HV services within the ANC/MNCH settings.

Delivering ART in TB treatment settings and TB treatment in HIV care settings

Collaboration between TB/HIV services is crucial to address the impact from the two diseases. This should span all the key levels from the national program through the district health system and facilities to the communities.

This collaboration should embrace the following thematic areas:

- Establishing mechanisms for collaboration between HIV and TB services
- Reducing the burden of TB in PLHIV and
- Reducing the burden of HIV in TB patients

For efficient coordination of TB/HIV activities the following areas are being addressed:

- Coordinating body at central level (TB/HIV Technical Advisory Committee), at district level (HIV/TB Coordination Meetings), at facility level (Multidisciplinary Teams) to manage TB/HIV services.
- Surveillance of HIV prevalence among TB patients. All presumptive and confirmed TB patients are offered HIV counselling and testing upon diagnosis.
- Joint TB/HIV planning, joint resource mobilization (both financial and human), capacity development (including training),TB/HIV advocacy, communication and social mobilization (ACSM)
- Joint operational research activities to inform national policy and strategy development so as to improve service delivery.
- Joint monitoring & evaluation of collaborative TB/HIV activities. This ensures timely assessment of quality, effectiveness, coverage and delivery of collaborative TB/HIV activities.
- Joint enhancement of community involvement in collaborative TB/HIV activities through support groups for PLHIV, DOT supporters, and community-based organizations. Communities can also be mobilized to help implement collaborative TB/HIV activities.

Integration of TB and HIV services at the facility level is necessary for ensuring effective TB/HIV collaboration.

TB and HIV interventions should be introduced at all levels within the district health system.

TABLE 12.2: TB AND HIV INTERVENTIONS AT VARIOUS LEVELS OF THE HEALTH SYSTEM

LEVEL OF HEALTH CARE	TB/HIV INTERVENTIONS
**HOME AND COMMUNITY**<br>Community based organizations, NGOs, faith based organizations, government community health programmes	• IEC activities regarding TB, HIV, and STI Condom promotion<br>• Nutritional advice and support<br>• Psychological support<br>• Community HTC<br>• Community screening and DOT for TB<br>• Community-based palliative and terminal care
**PRIMARY CARE**<br>Government health centres or clinics, mission health centres, NGO health centres, private health centres	• HTC and HIV prevention<br>• TB case finding and treatment<br>• Intensified case finding<br>• Isoniazid/Cotrimoxazole provision<br>• Condom promotion<br>• STI syndromic management<br>• Management of HIV related opportunistic infection and palliative care<br>• Prevention of mother to child transmission<br>• ART
**SECONDARY CARE**<br>Government hospitals, mission hospitals, private hospitals	• Diagnosis and treatment of HIV-related diseases<br>• In patient palliative care<br>• Diagnosis and management of complications or severe presentations of TB/HIV disease

SECTION 12.4: HUMAN RESOURCES

Human resources are a critical component of the health system in the delivery of HIV services. Both pre-and in-services training for health workers play a key role in building competences and skills to support rapid scale up of ART programmes. In addition, mentorship and supervision of health workers and community-based care providers is necessary to ensure high quality HIV care services. Newer approaches to learning, including distance learning and online courses, should be used to support the classroom- based learning which may be costly and more time-consuming.

The increase in the burden of chronic care for PLHIV requires adequate numbers of care providers to provide life-long care to ensure good treatment outcomes. This is against a backdrop of a mismatched and constrained staffing establishment within the MOH to adequately meet the HIV burden. The situation demands proactive human resource strategies such as task-shifting to meet the current challenges.Task-shifting involves the rational redistribution of tasks among health workforce such that specific tasks are reassigned as appropriate from highly qualified to lesser qualified health workers to improve efficiencies and effectiveness in the use of available workforce. Task-shifting helps to improve access to services at health centres where there are no physicians. On the other hand, physicians are able to devote more time managing complicated cases including drug toxicities, treatment failure and comorbidities. It is important to establish and maintain mentoring and supportive supervision to ensure quality is not compromised by task-shifting.

- **Trained nurses, midwives and other clinicians should initiate first-line ART**
- **Trained nurses, midwives and other clinicians should maintain ART**
- **Trained and supervised community health workers can supply stable patients pre-packed ARVs between regular clinical visits**

SECTION 12.5: LABORATORY AND DIAGNOSTIC SERVICES

Laboratory and diagnostic services are an essential component of comprehensive ART service package. Since HIV diagnostic services occur at health facility and community level by different health workers, the need for strengthening relevant quality assurance systems is paramount.The 2013 WHO HIV guidelines strongly recommend use of viral load monitoring as a better tool for monitoring HIV treatment response. Consequently, laboratory services will support the expansion of viral load testing capacity to meet the country requirements.

In order to strengthen the network of laboratory and diagnostic services, it is important to consider the following:

- Expand laboratory networks to support and monitor decentralization and integration of testing services or to provide effective referral system for laboratory services
- Strategic deployment of diagnostics platforms to optimize utilization
- Standardize testing methods to streamline procurement, quality assurance, maintenance and training
- Use of high quality and evaluated diagnostics before introduction into the system
- Design/strengthen suitable supply chain management system for laboratory commodities and equipment maintenance
- Mobilize sufficient resources to support laboratory services

> The need for strategic planning between the laboratory directorate and HIV programme manager for phasing- in of viral load technology is critical to inform the quantification of the requirements, resource mobilization, training and implementation.

In addition to the above considerations; laboratory services should build an efficient system for sample – results transportation and expedited result reporting and data management to reduce turnaround time for EID, CD4 and viral load tests. There is need to leverage on existing CD4 and EID networks. It is recommended that dried blood spots are used as a tool to increase access to viral load testing. Preferably, a centralized laboratory services model for viral load testing should be used.

Quality management systems for laboratory services need to be in-built including external quality assessments and internal controls. Testing sites should be supported to enrol into external quality control programme for proficiency testing. Tools for standard operating procedures should be set up and used at all levels. Service agreements for equipment should be in place and equipment serviced according to service contracts.

SECTION 12.6: PROCUREMENT AND SUPPLY MANAGEMENT SYSTEM

The need to ensure continuous availability of quality and affordable medicines cannot be over emphasized. PSM system should be strengthened to cope with an increasing volume of patients that require medicines as the ART proramme scales up. With decentralization and integration of ART services with other services such TB and MNCH, more should be done to ensure uninterrupted supply of ARVs and OI medicines at peripheral health centres and in multiple care settings. The entire PSM cycle including selection, procurement, storage and distribution, use and monitoring, should be well managed.

Table 12.3 - Checklist of pharmaceutical supply chain management issues

Phase	Activities
Planning and Product Selection	Update National Medicines List to include newer ARV regimens, OI medicines and diagnostics<br>Quantify and forecast medicine requirements considering ART scale up plan
Procurement	Procure medicines from pre-qualified suppliers<br>Procure generic medicines to reduce medicines costs<br>Establish a system that fosters openness and transparency in engaging potential suppliers<br>Establish and implement robust system for testing quality of medicines before use<br>Ensure sufficient buffer stock of medicines at central and service delivery level
Storage, distribution, rational use and monitoring	Secure appropriate medicines storage capacity at central and facility levels<br>Establish/strengthen effective distribution mechanism for medicines and related commodities<br>Implement effective monitoring and management systems for PSM including logistics management information system and electronic medicine dispensing system<br>Institute a pharmaco-vigilance system to monitor adverse drug events

Source: Adapted from 2013 WHO HIV Guidelines

CHAPTER 13: PROGRAMME MONITORINGAND EVALUATION

SECTION 13.1: DEFINITIONS

What is Patient Monitoring?

Patient monitoring is the routine collection; compilation; analysis and use of individual patient data or a ***group (cohort) of patients*** for decision making. Data is collected over time and across service delivery points. The information can be paper based or electronic. This is also called "**patient tracking**" and it provides important information for ***patient management.***

What is Patient Management?

Based on the relationship between providers on a clinical team and the ***individual patient*,** this is generating, planning, organizing, and administering medical and nursing care services for patients, assisted by written records. It is also called "**clinical management**" or "**clinical monitoring**".

What is Programme Monitoring?

This is on-going collection of priority information about a programme to determine if it is operating according to plan. It provides ongoing information on programme implementation and functioning*.* It is done at facility, district and national levels.

Purpose of patient monitoring

Patient monitoring is an important part of high quality patient care. Monitoring involves documenting all patient encounters by keeping regular and accurate records of key aspects of the care and treatment that is offered. This makes it possible to capture the history of a patient or group of patients over time and across different clinical sites and to collect data for reporting on and evaluating patient care at regular intervals.

In the context of facility-based HIV and AIDS care, monitoring offers three major benefits:

- It provides essential information for individual case management.
- It provides key information for managing the health facility (e.g., for ordering drugs and supplies or for making quality improvements).
- It provides information for operating and improving an HIV/AIDS programme at the facility, district, national, and international levels.

SECTION 13.2: OVERVIEW OF THE PATIENT MONITORING SYSTEM

1. The paper patient monitoring system includes eight paper items:
2. A short patient-held card (Bukana)
3. HIV Care/ART card (which is kept at the facility)
4. HIV Care Pre-ART Register
5. ART register
6. HIV Care /ART monthlyreportbook
7. Cohort analysis report
8. Appointment book
9. ART Referral form

FIGURE 13.1: OVERVIEW OF DATA FLOW FROM PATIENT CARD TO THE TWO REGISTERS AND TWO

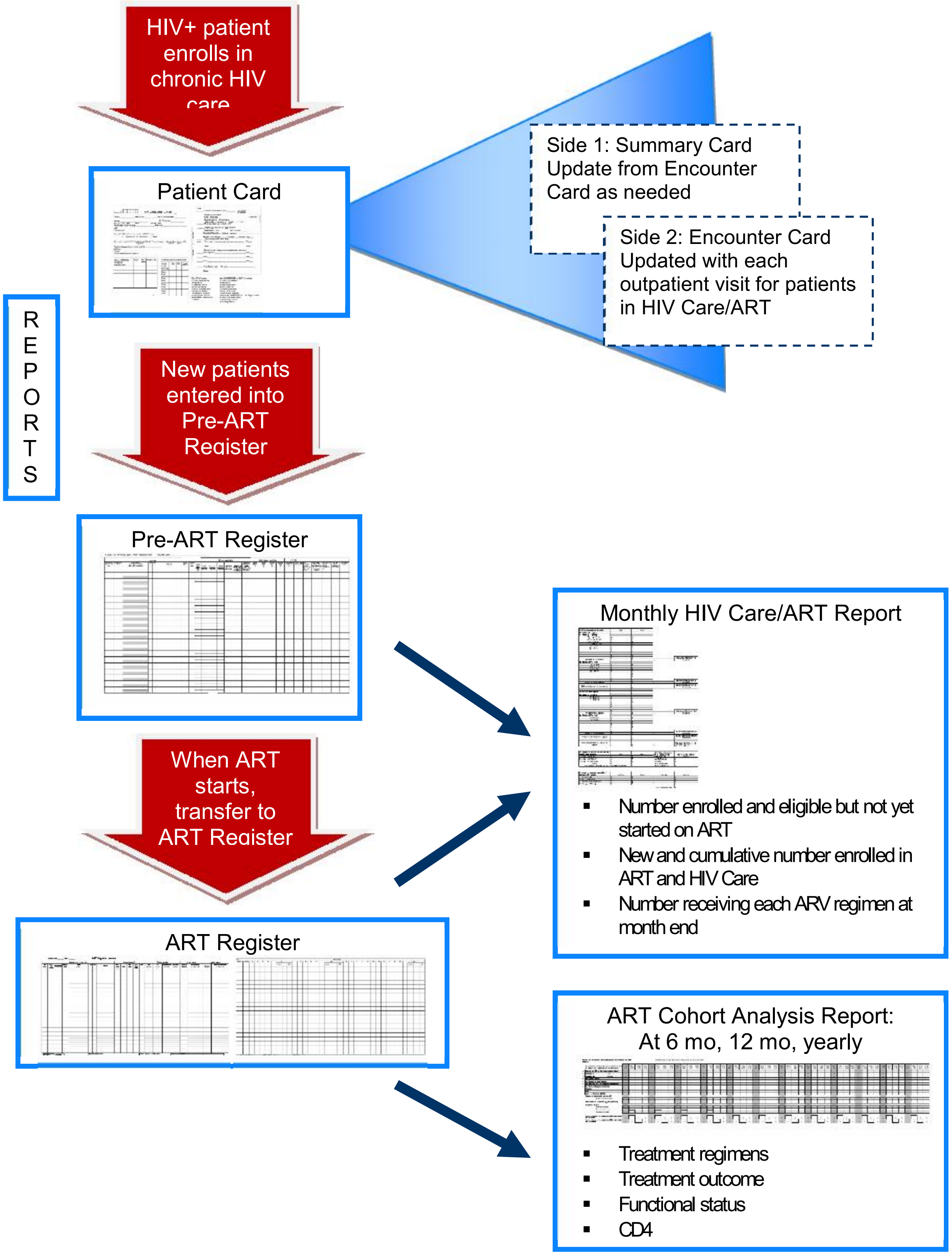

SECTION 13.3: MONITORING IMPLICATIONS OF 2013 RECOMMENDATIONS

TABLE13.1: IMPLICATIONS FOR MONITORING OF THE KEY RECOMMENDATIONS

Summary of new recommendation areas	Implications for monitoring
HIV testing and counselling	• Monitor the uptake of community-based HIV testing strategies and testing servicesfor adolescents,including systemsfor linkages to care
When to startART	• Monitor the number and percentage of different populations (such as adults, adolescents, children and pregnant and breastfeeding women) who have initiated ART based on the new eligibility criteria<br>• Review the monitoring system to assess what disaggregation is needed for what purpose (such as CD4 counts ≤200 cells/mm³ to routinely monitor late diagnosis or CD4 counts ≤350 cells/mm³ and 350–500 cells/mm³ to periodically assess the distribution of CD4 when ART is initiated) and how to best collect the relevant data, and age disaggregation of children (such as <2 years and <5 years).
Which ART regimen to start	• Monitor the first- and second-line ART regimens people are receiving<br>• Monitor the phasing out and/or introduction of specific drugs<br>• Monitoring tools may need to be adjusted to reflect new regimen options
Response to ART and diagnosing treatment failure	• Monitor the percentage of people receiving ART who had a viral load test and received the results<br>• Monitor the reasons for switching ART regimen
Service Delivery	• Monitor retention and adherence among various populations<br>• Monitor the integration of ART into facilities providing maternal and child health services, TB services and drug dependence services if planned by documenting the facilities providing ART<br>• Monitor whether the initiation and maintenance of ART has been decentralized as planned at various facilities by documenting the expansion of ART facilities<br>• Monitor the functionality of linkages from maternal and child health services, TB services and drug dependence services to HIV care and ART and linkages between communities, peripheral facilities and hospitals by documenting transfers
Task shifting	• Monitor the number of non-physician clinicians, midwives and nurses who are trained in ART<br>• Monitor the number of non-physician clinicians, midwives and nurses who are initiating first-line ART and maintaining ART and the number of people they have initiated or maintained on ART<br>• Monitor the number of community health workers who are trained and are dispensing ART between regular clinical visits, and capture the number of people to whom they dispense ART

SECTION 13.4: OTHER MONITORING CONSIDERATIONS

HIV drug resistance poses a significant threat to the success of the national HIV programme. Drug resistance results in more rapid virologic failure among people receiving first-line regimens and increases the need for second-line regimens, which may be associated with greater toxicity, adverse events, poorer adherence and higher costs. Drug resistance may also affect the ability to prevent HIV transmission using ARV-based pre- or post-exposure prophylaxis or topical microbiocides. Surveillance of drug resistance should be an integral component of national HIV programme.

Surveillance data should inform the selection of first- and second-line regimens for ART, as well as ARV drugs for PMTCT, to optimize treatment outcomes within a public health approach.

Monitoring early warning indicators for HIV drug resistance

Early warning indicators use existing clinic and pharmacy records to assess the factors associated with the emergence of HIV drug resistance at the level of ART programmes and clinics. These factors include ART prescribing practices; drug supply continuity; adherence to ARV drug regimens measured by on-time pick-up of ARV drugs; retention in care; and viral load suppression, when available.

The monitoring of early warning indicators will be integrated into a national monitoring and evaluation system and provides the information needed to address practices that may lead to poor outcomes and HIV drug resistance.

Surveys to monitor acquired HIV drug resistance and associated factors in populations receiving ART.

The WHO generic protocol for monitoring acquired HIV drug resistance uses a standardized survey methodology to assess population-level virologic suppression at the national level and the emergence of HIV drug resistance among populations receiving treatment. Performed regularly at representative sites, these surveys provide evidence for action at the programme and clinic level to minimize HIV drug resistance. They also provide evidence to optimize the selection of first- and second-line ART regimens.

Surveys to monitor pre-treatment HIV drug resistance

The WHO generic protocol for surveillance of pre-treatment HIV drug resistance provides a nationally representative estimate of HIV drug resistance in populations initiating therapy. Performed regularly at representative ART clinics, these surveys support national, regional and global decision-making regarding the choice of first-line regimens.

Surveillance of transmitted HIV drug resistance among individuals recently infected with HIV.

The WHO generic protocol for surveillance of transmitted HIV drug resistance provides estimates of transmitted HIV drug resistance in recently infected populations, and the results should contribute to ART policy decisions, including guidelines on ART regimens and HIV prophylaxis.

TABLE 13.2NATIONAL ARV PROGRAMME INDICATORS

Level	Area	Indicator	Recommended method	Frequency
Input	National policy & guidelines	Core 1: Existence of national policies, strategy, and guidelines for ART programmes	Key informant survey	Every 2 years
Process	Programme coverage (initial scale-up)	Core 2: Percentage of districts or local health administration units with at least one health facility providing ART services in-line with national standards	Record or programme reviews, or health facility survey	Annual during scale-up, every 2 years thereafter
	Drug supply	Core 3: Percentage of ARV storage and delivery points experiencing stock-outs in the previous 6 months<br><br>Additional Indicator 3.1: Percentage of ARV storage and delivery points	Drug tracking system, programme reports	Annual during scale-up, every 2 years thereafter

		meeting the minimum quality criteria (in addition to having no stock-outs).		
	Human resources	Core 4: Number of health workers trained on ART delivery in accordance with national or international standards	Programme records, or health facility surveys	Annual during scale-up, every 2 years thereafter
Output	ART programme coverage	Core 5: Percentage of health facilities with systems and items to provide ART services	Health facility survey with observation component	Annual during scale-up, every 2-4 years thereafter
	Comprehensive care coverage, including prevention	Core 6: Percentage of health facilities with ART services that also provide comprehensive care, including prevention services, for HIV positive clients	Health facility surveys	Annual during scale-up, every 2-4 years thereafter
Outcome	People on treatment	Core 7: Percentage of people with advanced HIV infection receiving ARV combination therapy	Review of programme monitoring data	Six-monthly during scale-up, annually thereafter
	Continuation of first-line regimens	Core 8: Continuation of first-line regimens at 6, 12 and 24 months after initiation	Review of patient registers	Continuous data collection, aggregated on yearly basis
Impact	Survival	Core 9: Survival at 6, 12, 24, 36, etc. months after initiation of treatment	Review of patient registers	Continuous data collection, aggregated on yearly basis

ANNEXES:

ANNEX 1: DEVELOPMENTAL MILESTONES IN INFANTS AND YOUNG CHILDREN

Age	Psychosocial	Gross Motor	Fine Motor/Visual	Communication / Hearing
1 month	▪ follows faces to the midline	▪ moves all extremities equally<br>▪ lifts head when lying on stomach	▪ opens hands spontaneously	▪ startled by loud sounds<br>▪ cries<br>▪ quiets when fed and comforted
2 months	▪ follows faces past midline<br>▪ smiles responsively	▪ lifts head up 45 degrees when on stomach	▪ looks at own hand	▪ makes baby sounds (cooing, squealing, gurgling)
3 months	▪ recognizes mother<br>▪ smiles responsively	▪ supports head for a few seconds when held upright	▪ opens hands frequently	▪ responds to voices<br>▪ laughs
4 months	▪ follows an object with eyes for 180 degrees<br>▪ regards own hand<br>▪ anticipates food on sight	▪ bears weight on legs<br>▪ good neck control when pulled to sitting<br>▪ lifts chest and supports self on elbows when pulled to sit	▪ brings hands together in midline (claspshands)<br>▪ grabs an object (such as a rattle)<br>▪ reaches for objects	▪ turns head to sound
6 months	▪ reaches for familiar people	▪ rolls from stomach to back or back to stomach<br>▪ sits with anterior support	▪ plays with hands by touching them together<br>▪ sees small objects such as crumbs	▪ responds to name<br>▪ babbles
9 months	▪ indicates wants/desires<br>▪ waves bye-bye<br>▪ stranger anxiety	▪ can sit without support<br>▪ creeps or crawls on hands and knees	▪ looks for a toy when it falls from his/her hand<br>▪ takes a toy in each hand<br>▪ transfers a toy from one hand to the other	▪ responds to soft sounds such as whispers
12 months	▪ has separation anxiety<br>▪ social interactions<br>▪ intentional and goal-directed	▪ pulls self up to standing position<br>▪ walks with support	▪ points at objects with index finger	▪ says at least one word<br>▪ makes “ma-ma” or “da-da” sounds<br>▪ locates sounds by turning head
15 months	▪ imitates activities<br>▪ finds a nearby hidden object	▪ can take steps by himself<br>▪ can get to a sitting position from a lying position	▪ can stack one cube on top of another	▪ able to say mama and dada to respective parents
18 months	▪ initiates interactions by calling to adult	▪ walks without help	▪ takes off own shoes<br>▪ feeds self	▪ says at least 3 words
2 years	▪ does things to please others<br>▪ parallel (imitative) play	▪ runs without falling	▪ looks at pictures in a book<br>▪ imitates drawing a vertical line	▪ combines two different words

ANNEX 2: DEVELOPMENTAL RED FLAGS

Birth to 3 months	▪ Failure to respond to environmental stimuli<br>▪ Rolling over before 2 months (hypertonia)<br>▪ Persistent fisting at 3 months
4-6 months	▪ Poor head control<br>▪ Failure to smile<br>▪ Failure to reach for objects by 5 months
6-12 months	▪ No baby sounds or babbling<br>▪ Inability to localize sounds by 10months
12-24 months	▪ Lack of consonant production<br>▪ Hand dominance prior to 18 months (contralateral weakness)<br>▪ No imitation of speech and activities by 16 months
Any age	▪ Loss of previously attained milestones

ANNEX 3: GROSS MOTOR MILESTONES IN INFANTS AND YOUNG CHILDREN

Windows of achievement for six gross motor milestones

World Health Organization

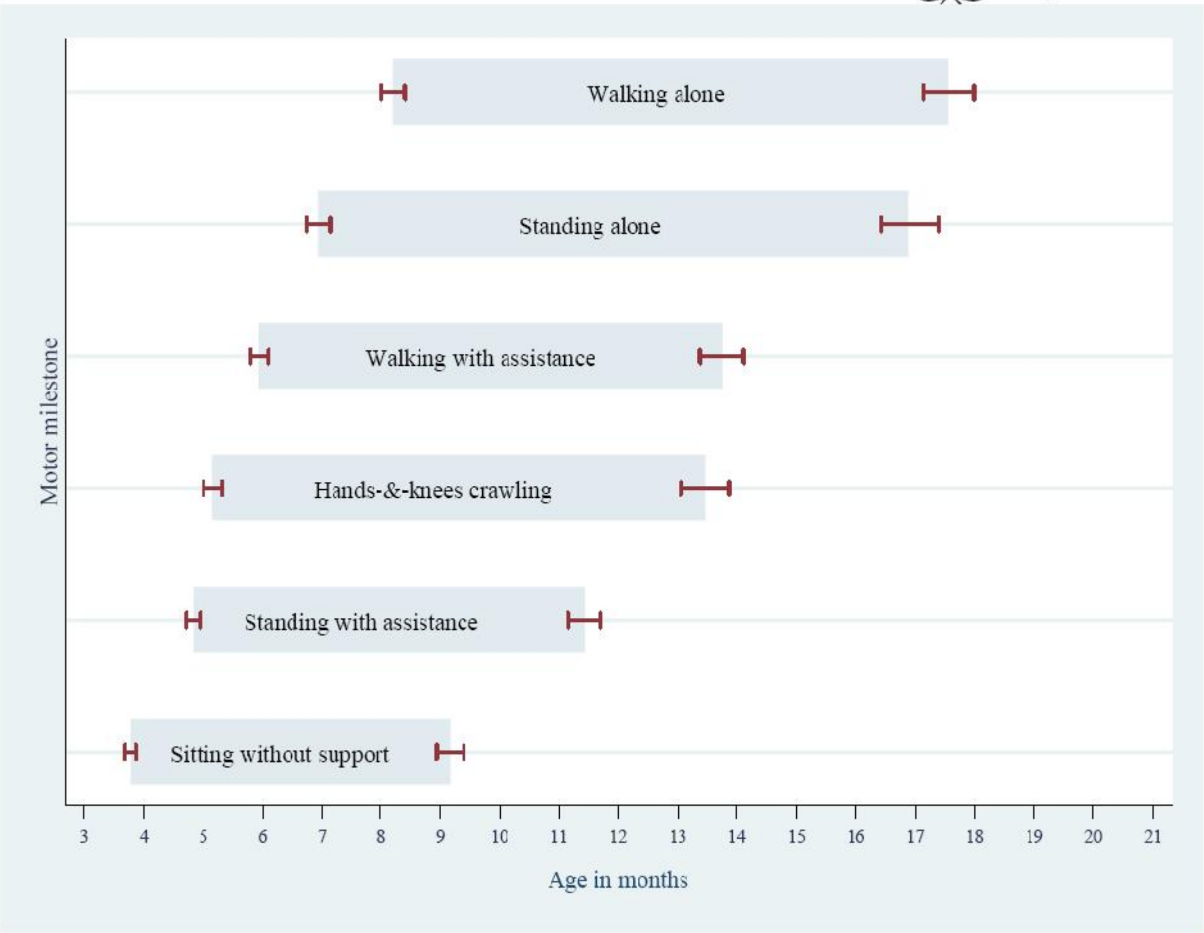

Reference: WHO Multicentre Growth Reference Study Group. WHO Motor Development Study: Windows of achievement for six gross motor development milestones. Acta Paediatrica Supplement 2006;450:86-95.

ANNEX 4: WEIGHT-BASED DOSING OF ANTIRETROVIRALS AND PROPHYLACTICS

Ministry Of Health - Lesotho
Weight-Based Dosing Chart for Antiretroviral Drugs and Prophylactics

	Medication	Strength	Medication dosages by weight band					Strength	Dosages by weight band	Strength	Dosages by weight band
			3-5.9 kg	6-9.9 kg	10-13.9 kg	14-19.9 kg	20-24.9 kg		25-34.9 kg		>35 kg
Once Daily ART	ABC/3TC	60/30 mg	2	3	4	5	6	600/300 mg	1	600/300 mg	1
	EFV	200 mg	-	-	1	1.5	1.5	200 mg	2	600 mg	1
	ATV/r		-	-	-	-	-		-	300/100mg	1

	Medication	Strength	3-5.9 kg		6-9.9 kg		10-13.9 kg		14-19.9 kg		20-24.9 kg		Strength	25-34.9 kg		Strength	>35 kg	
			AM	PM	AM	PM	AM	PM	AM	PM	AM	PM		AM	PM		AM	PM
Twice Daily ART	ABC/3TC	60/30 mg	1	1	1.5	1.5	2	2	2.5	2.5	3	3	600/300 mg	-	1	600/300 mg	-	1
	LPV/r	80/20 mg/ml	1 ml	1 ml	1.5 ml	1.5 ml	2 ml	2 ml	2.5 ml	2.5 ml	3 ml	3 ml	100/25 mg	3	3	200/50 mg	2	2
	LPV/r	100/25 mg	-		-		2	1	2	2	2	2						
	AZT/3TC	60/30 mg	1	1	1.5	1.5	2	2	2.5	2.5	3	3	300/150 mg	1	1	300/150 mg	1	1
	AZT/3TC/NVP	60/30/50 mg	1	1	1.5	1.5	2	2	2.5	2.5	3	3	300/150/200	1	1	300/150/200	1	1
	NVP	10 mg/ml	5 ml	5 ml	8 ml	8 ml	10 ml	10ml	-		-		200 mg	1	1	200 mg	1	1
	NVP	200 mg	-		-		0.5	0.5	1	0.5	1	0.5						
	RAL		-		-		-		-		-			-		400mg	1	1

	Medication	Strength	3-5.9 kg	6-9.9 kg	10-13.9 kg	14-19.9 kg	20-24.9 kg	Strength	25-34.9 kg	Strength	>35 kg
Prophylaxis	CTX	240 mg/5ml	2.5 ml	5 ml	5 ml	-	-	960 mg	1	960 mg	1
	CTX	480 mg	-	0.5	0.5	1	1				
	INH	100 mg	0.5	1	1.5	2	2.5	300 mg	1	300 mg	1

Weight-based ART Dosing for Children< 3 yrs and < 10kg on Concomitant Anti-Tuberculosis Therapy**

Weight (kg)	Paediatric Zidovudine/Lamivudine/Nevirapine (AZT/3TC/NVP) 60/30/50mg tabs (maintenance)		Additional Nevirapine (NVP) 10mg/ml Syrup to add	
	**AM Dose**	**PM Dose**	**AM Dose**	**PM Dose**
3-3.9	1	1	0	0
4-4.9	1	1	1ml	0
5-5.9	1	1	1.5ml	1.5ml
6-6.9	1.5	1.5	0	0
7-7.9	1.5	1.5	1ml	0
8-8.9	1.5	1.5	1.5ml	1.5ml
9-9.9	1.5	1.5	2ml	2ml
10-10.9	2	2	0	0

***Note that since Rifampicin is known to reduce levels of NVP, lead-in (once-daily)dosing of NVP for the first 2 weeks is not necessary when initiating NVP-containing ART with concomitantTB treatment.*

ANNEX 5: TALKING ABOUT HIV TO HIV-INFECTED CHILDREN

**<6 YEARS** Most children will not understand HIV or be able to keep it private
Suggestions for explaining HIV:
• You have a germ in your blood
• The germ hurts the healthy parts of your blood
• When the health parts are hurt, you get sick with coughing or diarrhoea or other things that make you feel bad
• The medicine will kill the germs so that your blood can become healthy again
• If you take your medicine every day you can stay healthy and stop the germ from making you sick
• You can always talk to your family (indicate which members) and to your doctors and nurses about being sick
Some questions that may come up with answers:
• Q: How did I get this germ?
• A: You were born with it, you have had it since you were a baby
• Q: Can you get rid of this germ?
• A: The medicine can get rid of most of it so you can stay healthy, but we cannot get rid of all of it.
• Q: When can I stop taking my medicine?
• A:You have to take your medicine everyday so that you can stay healthy, maybe one day doctors will be able to get rid of all the germs, but for now you have to take your medicine everyday.
**7-11YEARS** Not all children seek the same amount for information. Take your lead from the child as to how much information to provide. You can and should explain infection, immune depletion and the reason for taking drugs - without mentioning HIV in children where the child or the family is not ready for full disclosure. Keep information simple.
Suggestions for explaining HIV:
• You have come to the doctor because you have an illness-you may get sick some times
• You have a germ (virus) that lives in your blood – Ask what the child knows about germs and illness and correct misinformation
• Viruses make you sick and the doctors visits and medicines are needed to help you stay healthy
• The virus (HIV) kills the cells in your blood that helps you stay healthy
• The name of these cells are T- cells – the virus (HIV) kills T cells
• Without T-cells your body struggles to stay well and you get sick with coughing or other things that make you feel bad
• The medicine kill the virus (HIV) so that your T-cells can grow back and they can help you stay healthy
• If you stop taking your medicine the virus (HIV) will get stronger again and kill your T-cells then you will get sick again.
• We take blood so that we can measure the T-cells and as well as how much virus is in your blood.
• When you are doing well, we see lots of T-cells and very little virus
Explaining transmission:
• You got this virus when you were born. Your mother has the same virus. You got this virus from your mother
• You cannot get this virus by being friends or hugging or touching. It is ok to play and go to school. If you hurt yourself, you must not let other people touch your blood
Regarding privacy:
• We are explaining all this to you so that you can take better care of yourself
• This is private information. Indicate the person the child can discuss this with.
Some questions that may come up with answers:
• Q: Can you get rid of this virus?
• A: The medicine can get rid of most of it, so you can stay healthy, but cannot get rid of all of it. Currently there is no cure.
• Q: When can I stop taking medicine?
• A: You have to take your medicine everyday so that you can stay healthy. Maybe one day doctors will be able to cure

	HIV, but for now you have to take your medicine everyday
•	Q: Am I going to die?
•	A:If you take medicines everyday, you can stay healthy for a long time.
•	Q: How did my mom get HIV? – ALWAYS DEFER TO THE MOTHER

ANNEX 6: GRADING OF ARV TOXICITIES

Symptom (and diagnoses to consider, plus likely ARV responsible)	Grade 1	Grade 2	Grade 3	Grade 4
Abdominal pain +/- nausea	▪ Mild and transient (<24 hr)	▪ Food intake decreased (24 - 48 hrs)	▪ Minimal food intake (> 48 hrs)	▪ Patient too sick for outpatient treatment
▪ NRTI-associated pancreatitis or lactic acidosis<br>▪ NVP-related hepatitis	▪ No treatment needed, but have patient return early if pain worsens	▪ Encourage frequent small meals<br>▪ Give Metoclopramide 10 mg every 12 hours prn<br>▪ Take blood for ALT and Lipase (or Amylase) and reassess in 2-3 days	▪ Consider stopping all ARVs* if lipase or amylase > 4 times normal, or ALT > 400<br>▪ Also, check lactate level if patient has been on d4T for more than 4 months, to rule out high lactate as the cause	▪ Stop all ARVs and refer to hospital*
Vomiting	▪ Once per day and/or lasting < 3 days	▪ < 4 episodes per day and not dehydrated	▪ Vomits > 3 times per day, and dehydrated	▪ Dehydrated and too sick for outpatient treatment
▪ NRTI-associated pancreatitis or lactic acidosis<br>▪ NVP-related hepatitis	▪ Reassure patient, but have patient return early if worsens<br>▪ Consider giving Metoclopramide 10 mg every 12 hours prn	▪ Give ORT<br>▪ Encourage frequent small meals<br>▪ Give Metoclopramide 10 mg every 12 hours prn<br>▪ Take blood for ALT and Lipase (or Amylase) and reassess in 2-3 days	▪ Give ORT<br>▪ Give Metoclopramide 10 mg every 12 hours prn<br>▪ Consider stopping all ARVs* until blood results (Lipase and ALT) are available<br>▪ Check lactate level if patient has been on d4T for more than 4 months, to rule out high lactate as the cause	▪ Stop all ARVs and refer to hospital*<br>▪ Rehydrate with intravenous (IV) normal saline<br>▪ Check lactate level to rule out high lactate as the cause
Psychological	▪ Dizziness	▪ Vivid dreams	▪ Mood changes or persistent disturbing dreams	▪ Acute psychosis, hallucinations, confused behaviour
▪ EFV	▪ Reassure patient<br>▪ Confirm EFV is being taken at night	▪ Reassure patient<br>▪ Symptom will go away after few weeks	▪ Give Chlorpromazine 50 mg at night as needed	▪ Stop all ARVs and refer to hospital*<br>▪ Perform Lumbar Puncture to rule out meningitis<br>▪ Only restart ARVs when symptoms have fully resolved (use NVP instead of EFV)
Skin rash	▪ Red, itchy	▪ Maculo-papular rash or dry scales	▪ Blisters or moist loss of skin	▪ Rash involves mucous membranes or eyes +/- sloughing of skin

Symptom (and diagnoses to consider, plus likely ARV responsible)	Grade 1	Grade 2	Grade 3	Grade 4
▪ NVP (more commonly)<br>▪ EFV(but also consider TB meds or Co-trimoxazole as possible causes)	▪ Reassure, but have patient return early if worsens<br>▪ Consider giving Chlorpheniramine 4 mg every 8 hours prn, if itch is significant	▪ Give Aqueous cream +/- 0.1% Betamethasone<br>▪ Consider giving Chlorpheniramine 4 mg every 8 hours prn<br>▪ Check ALT, and reassess in 2-3 days<br>▪ Patient to return early if rash worse, or abdominal pain	▪ Stop all ARVs*, check ALT, and refer to doctor<br>▪ Give Chlorpheniramine 4 mg every 8 hours as needed<br>▪ When symptoms have resolved, restart ARVs using EFV (if rash was due to NVP)	▪ Stop all ARVs and refer to hospital<br>▪ ARVs can be restarted once patient is stable but avoid NVP or EFV in the future (instead, use Kaletra in the first-line regimen)
Elevated ALT *(in U/L)*	▪ 50 - 100	▪ 100 - 200	▪ 200 - 400	▪ > 400
▪ NVP (more commonly)<br>▪ EFV	▪ Continue ARVs, but recheck ALT in 1 month	▪ Continue ARVs if no other problem<br>▪ Recheck ALT again after 2 weeks	▪ Switch NVP to EFV (unless patient is in the first trimester of pregnancy)<br>▪ Monitor ALT weekly to ensure a fall in ALT	▪ Stop all ARVs and refer to hospital*<br>▪ Check ALT frequently to ensure it returns to normal<br>▪ Restart ARVs with EFV (unless in the first trimester of pregnancy)
Anaemia *(low Haemoglobin, in gm/dl)*	▪ 8 - 9,4	▪ 7 - 7,9	▪ 6,5 – 6,9	▪ < 6,5
▪ AZT	▪ Examine patient to rule out bleeding, or serious problem (including active TB)<br>▪ If no problem, continue ARVs<br>▪ Recheck Hb in 2 weeks	▪ Examine patient to rule out bleeding, or other serious problem (including disseminated TB)<br>▪ If no problem, continue ARVs<br>▪ Recheck Hb in 7 days	▪ Examine patient to rule out bleeding, and refer to doctor for assessment<br>▪ If no problem, switch AZT to ABC (or TDF, if contraindication to ABC)<br>▪ Recheck Hb weekly, to ensure rise in Hb<br>▪ Consider sending blood to lab for FBC (to rule out coexistent Neutropenia)	▪ Examine patient to rule out bleeding, and refer to hospital<br>▪ Consider blood transfusion<br>▪ Switch AZT to ABC (or TDF), or consider stopping all ARVs*
Neutropenia *(low absolute neutrophil count)*	▪ 1 - 1,5 x 106	▪ 0,75 - 1,0 x 106	▪ 0,5 - 0,75 x 106	▪ <0,5 x 106

Symptom *(and diagnoses to consider, plus likely ARV responsible)*	Grade 1	Grade 2	Grade 3	Grade 4
▪ AZT	▪ Continue ARVs<br>▪ Recheck FBC (+ differential) in 2 weeks	▪ Examine patient for any signs of infection<br>▪ Continue ARVs<br>▪ Recheck FBC (+ diff) in 2 weeks	▪ Examine patient for any signs of infection<br>▪ If no serious infection, switch AZT to d4T (or TDF, if d4T contraindicated)<br>▪ Recheck FBC (+ diff) weekly to ensure rise in absolute neutrophil count	▪ Examine patient for any signs of infection<br>▪ If serious infection, refer to doctor for assessment<br>▪ Switch AZT to d4T (or TDF), or consider stopping all ARVs*
Hyperlactatemia = High Lactate<br>*(which will progress to lactic acidosis if not identified early, or managed appropriately!)*	▪ Asymptomatic<br>▪ and/or Lactate < 2.5	▪ Symptomatic hyperlactatemia<br>▪ (weight loss, fatigue, peripheral neuropathy, nausea, etc)<br>▪ Lactate between 2.5 – 3.5	▪ Symptomatic hyperlactatemia with risk of progression to lactic acidosis<br>▪ (Think of lactic acidosis if symptoms of hyperlactatemia, plus abdominal pain, vomiting, shortness of breath, and ketones on urine dipstick.)<br>▪ Lactate between 3.6 – 4.9	▪ Lactic acidosis (note that this condition can be fatal, if not managed appropriately and immediately!)<br>▪ Lactate 5.0 or greater, or RR > 20 (even if lactate level is < 5.0)
▪ AZT *(less commonly)*	▪ Continue ARVs	▪ Examine patient to rule out new infection<br>▪ Check urine for ketones (using dipstick) to rule out acidosis<br>▪ Monitor lactate level weekly until lactate normalizes	▪ Examine patient to rule out new infection and/or acidosis<br>▪ Check urine for ketones (using dipstick) to rule out acidosis<br>▪ Monitor lactate level weekly until lactate normalizes<br>▪ If lactate does not improve, stop all ARVs (and consider giving Kaletra 4 tabs BD for one week to prevent resistance to NNRTI)<br>▪ When lactate level is normal, use TDF (avoid AZT)	▪ Admit to hospital<br>▪ Rehydrate with intravenous fluid (+/- bicarbonate)<br>▪ Investigate for new infection (pneumonia, sepsis, TB, etc)<br>▪ Consider giving i.v. Ceftriaxone for 3 days<br>▪ Monitor lactate level frequently until normal<br>▪ When lactate level is normal, restart ARVs with Tenofovir (TDF)<br>▪ Avoid d4T, ddI, and all other NRTIs in the future

**Whenever possible, use 'tail protection' to prevent the development of resistance to NVP or EFV. This means stopping NVP or EFV first, and continuing 2 NRTIs (TDF/3TC, AZT/3TC or d4T/3TC, as appropriate) for one week*

ANNEX 7: HIV CHRONIC CARE/ART REFERRAL FORM

Referral From:

Referral to: .

Name: Age:

Physical Address: sex:

Date confirmed HIV positive: dd [ ] mm[ ] yy [ ]

Pre-ART information(if client is referred before starting ARVs)
HIV Chronic Care no:<br><br>Date Enrolled in Chronic Care: ..<br><br>Recent CD4 Count & Clinical Stage: [CD4] .Clinical stage:<br><br>Date of last assessment: ..
ART information: (if client is referred already on ARVs)
ART Unique number: .Date started ART: dd[ ] mm [ ] yy [ ]<br><br>COHORT: mm [ ] yy [ ]<br><br>At Start of ART: Weight: : .Functional status:<br><br>Clinical Stage: [1] [2] [3] [4] (please tick)<br><br>CD4 Count: ..Date done: .<br><br>Initial regimen:<br><br>Current regimen (During transfer):

Laboratory Investigations:

Date	Hb/FBC	ALT/LFT	CD4	Creatinine	HBs(AG)	HCV

Comments:

. .

Name of referring Doctor: .Date
Signature: Phone no:

ANNEX 8: ADHERENCE CONTRACT

ADHERENCE PLAN:

Please tick after each statement once it has been reviewed with the applicable individual(s):

1.I understand that antiretroviral drugs (ARVs) against HIV stop the virus from multiplying, leading to a better quality of life, although they are not a cure for HIV.HIV is a lifelong infection and ARVs are a lifelong treatment.Therefore, even if I/my child feels better after starting the ARVs, I understand that if the ARVs are stopped, sickness will resume.

[ ] Primary Caretaker [ ] Patient (if disclosed to) [ ] Caretaker #2, if applicable [ ] Caretaker #3, if applicable

2. I understand that taking all of the ARV medications together as prescribed is critical to treatment success, and that even missing 1 dose may result in permanent drug failure and sickness. I will not miss any doses. If I do miss doses, I will ask the clinic for help since it is so important.

[ ] Primary Caretaker [ ] Patient (if disclosed to) [ ] Caretaker #2, if applicable [ ] Caretaker #3, if applicable

3. I understand that I/my child cannot miss any doses. Therefore, I will return on time for each clinic appointment for ARV refills. If I run out of ARVs in advance of my/mychild's appointment due to an accident/spill, then I will return to the clinic immediately for a refill on a clinic day.

[ ] Primary Caretaker [ ] Patient (if disclosed to) [ ] Caretaker #2, if applicable [ ] Caretaker #3, if applicable

4. I understand that since all the ARVs must be taken together to work, I will not stop any one of the medications without consultation with a doctor. I will not give away or sell the ARVs to anyone since this will be hurtful to me/my child and to the other person. I understand that if I stop one or more ARVs without the advice of a doctor, I may seriously hurt my/my child's future treatment options because of HIV resistance. The first ARV regimen is the most important/effective. If it fails, the options are limited.

[ ] Primary Caretaker [ ] Patient (if disclosed to) [ ] Caretaker #2, if applicable [ ] Caretaker #3, if applicable

5. I understand that the ARVs must be taken at the same time of day every day. However, should I forget to administer/take a dose, I should administer/take it as soon as I remember. However, if it is 6 hours past the time that the dose was due (for twice daily ARVs) or 12 hours past the time the dose was due (for once daily ARVs), then skip the missed dose and continue your regular dosing schedule. Do not take a double dose to make up for a missed one.

[ ] Primary Caretaker [ ] Patient (if disclosed to) [ ] Caretaker #2, if applicable [ ] Caretaker #3, if applicable

6. I understand that all medications may have associated side effects. These may include temporary weakness, rash, tiredness or lack of blood, loose stools, tingling sensation in the feet, vivid dreams, or others. I will come to the clinic if any side effects occur and will not stop any medications unless directed to do so by a doctor.

[ ] Primary Caretaker [ ] Patient (if disclosed to) [ ] Caretaker #2, if applicable [ ] Caretaker #3, if applicable

7. If I/my child vomits within 30 minutes of taking the medication, or if I can see the ARVs in the vomit itself, then I will repeat the dose.
[ ] Primary Caretaker [ ] Patient (if disclosed to) [ ] Caretaker #2, if applicable [ ] Caretaker #3, if applicable

8. I will bring my/my child's ARVs and/or pill box to every visit.

[ ] Primary Caretaker [ ] Patient (if disclosed to) [ ] Caretaker #2, if applicable [ ] Caretaker #3, if applicable

9. For caretaker(s): I am fully committed to making certain that the child I am caring for receives his/her ARVs. If I can no longer care for the child, I will let the clinic counselor know in as far advance as possible, so that another adult may be counseled to do so.

[ ] Primary Caretaker [ ] Caretaker #2, if applicable [ ] Caretaker #3, if applicable

10. For caretaker(s) of children only: I understand that I should encourage my child to be responsible for taking their ARVs; however, I understand that children must be directly monitored while swallowing the ARVs and I will closely supervise them successfully taking them.

[ ] Primary Caretaker [ ] Caretaker #2, if applicable [ ] Caretaker #3, if applicable

For all patients if disclosed to: I understand that since ARVs are not a cure, it is still possible for me to pass on the virus to someone else through my blood or through sexual intercourse. I understand how to avoid passing the virus to others, and I understand that it is possible for me to contract another strain of HIV, such that it is also in my best interest to protect myself from further infection.

[ ] Patient (if disclosed to)

12. For all female patients if disclosed to: I understand that it is still possible for me to transmit HIV mother-to-child during pregnancy, delivery, or breastfeeding even while on ARVs, although this risk is lower than among HIV-positive women not on ARVs. Since some ARVs may harm the developing fetes, I will inform my doctor if I am or plan to become sexually active or pregnant.

[ ] Patient (if disclosed to)

ADHERENCE COMMITTMENT:

By signing below, I commit to adhering to each and every dose of ARV medication for the rest of my/the child I care for's life:

______________________ Primary Caretaker Name	______________________ Primary Caretaker Signature	________ Date
______________________ Patient's Name (*if disclosed to)*	______________________ Patient's Signature	________ Date
______________________ Caretaker #2 Name *(if applicable; required, if High Risk)*	______________________ Caretaker #2 Signature	________ Date
______________________ Caretaker #3 Name *(if applicable)*	______________________ Caretaker #3 Signature	________ Date
______________________ Counselor Name	______________________ Counselor Signature	________ Date

TUMELLANO KAPA BOITLAMO BA HO NOA LITLHARE KA NEPO

Letsatsi:_______________ Nomoro ea Faele:______

U koptjoa ho ts'oaea ka mor'a hoba polelo ka 'ngoe e hlahlojoe le motho /batho ba lokelang.

1. Kea utloisisa hore litlhare tsa li ARV khahlanong le HIV, li thibela kokoana-hloko ho ikatisa, e leng ho lebisang bophelong ba boleng bo betere, leha e se pheko ea HIV. HIV ke ts'oaetso ea bophelo bohle 'me li ARV ke kalafo ea bophelo bohle. Kahoo, leha eba 'na kapa ngoan'aka a ikutloa a le betere kamor'a ho qala li ARV, kea utloisisa hore ha li ARV li khaotsoa, bokuli botla tsoela-pele hape.
[ ] Mohlokomeli oa mathomo [ ] Mokuli (haeba a se a boleletsoe boemo)
[ ] Mohlokomeli oa bobeli, haeba a le teng [ ] Mohlokomeli oa boraro haeba a le teng.

2. Kea utloisisa hore ho noa li ARV hammoho, joalo ka ha ho boletsoe, ho bohlokoa bakeng sa kalafo e atlehileng, 'me le hore leha ele ho fosa ho noa le se le seng sa litlhare ho ka baka ho hlolehela ruri hoa litlhare le bokuli. Nkeke ka fosa ho noa litlhare leha e le ha 'ngoe. Ha nka fosa, ke tla kopa cliniki hore e nthuse ka ha hoo ho le bohlokoa.
[ ] Mohlokomeli oa mathomo [ ] Mokuli (haeba a se a boleletsoe boemo)
[ ] Mohlokomeli oa bobeli, haeba a le teng [ ] Mohlokomeli oa boraro haeba a le teng.

3. Kea utloisisa hore 'na kapa ngoana'ka a keke a fosa ho noa litlhare, kahoo ke tla khutlela cliniking ka nako bakeng sa matsatsi ao ke a behetsoeng ho lata litlhare. Haeba litlhare li mphella pele ho nako e behiloeng ka baka la tsietsi kapa ho qhalana, ke tla khutlela cliniking hang-hang, ka letsatsi la cliniki la ts'ebetso.
[ ] Mohlokomeli oa mathomo [ ] Mokuli (haeba a se a boleletsoe boemo)
[ ] Mohlokomeli oa bobeli,haeba a le teng [ ] Mohlokomeli oa boraro haeba a le teng.

4. Kea utloisisa hore ka ha li ARV li lokela ho nooa kaofela, hammoho hore li tle li sebetse, nke ke ka khaotsa ho noa kapa ho noesa ngoana setlhare sefe kapa sefe ntle le ho botsa ngaka. Nke ke ka fana kapa ka rekisa li ARV ho mang kapa mang, ka ha hoo ho ba kotsi ho 'na kapa ngoana'ka le ho motho eo e mong. Kea utloisisa hore ha ke khaotsa ho noa kapa ho noesa ngoana'ka setlhare se le seng, kapa ho feta, ntle le boeletsi ba ngaka, nka baka kotsi kalafong ea kamoso ea ka kapa ea ngoana'ka, ka lebaka la manganga a HIV. Mokhahlelo oa pele oa kalafo ke 'ona o bohlokoahali. Ha o hloleha, menyetla ea kalafo e se e fokola.
[ ] Mohlokomeli oa mathomo [ ] Mokuli (haeba a se a boleletsoe boemo)
[ ] Mohlokomeli oa bobeli, haeba a le teng [ ] Mohlokomeli oa boraro haeba a le teng.

5. Kea utloisisa hore li ARV li lokela ho nooa ka nako e le 'ngoe letsatsi le letsatsi. Leha ho le joalo, ha nka lebala ho noa kapa ho noesa ngoana litlhare, ke lokela ho noa kapa ho mo noesa lithlare tseo hang ha ke hopola. Empa haeba ke hopola kamor'a lihora tse ts'eletseng (bakeng sa litlhare tse nooang habeli ka letsatsi), ke tla tlola litlhare tseo tse fetiloeng ke nako, ke tsoele-pele ka nako ea mehla. Nke ke ka noa kapa ka noesa ngoana litlhare habeli ele ho lefa tse fetiloeng ke nako kapa tseo nako ea tsona e fositsoeng.
[ ] Mohlokomeli oa mathomo [ ] Mokuli (haeba a se a boleletsoe boemo)
[ ] Mohlokomeli oa bobeli, haeba a le teng [ ] Mohlokomeli oa boraro haeba a le teng.

6. Kea utloisisa hore litlhare tsohle li ka ba le litla-morao. Litla-morao tsena li ka kenyeletsa ho fokolloa ke matla ha nakoana, lekhopho, mokhathala kapa khaello ea mali, ho choachoasela ha maoto, litoro tse matla, le tse ling. Ke tla tla cliniking haeba se seng sa litla-morao se iponahatsa, 'me nke ke ka khaotsa ho noa kapa ho noesa ngoana litlhare, ntle le ha ngaka e bolela joalo.

[ ] Mohlokomeli oa mathomo [ ] Mokuli (haeba a se a boleletsoe boemo)
[ ] Mohlokomeli oa bobeli, haeba a le teng [ ] Mohlokomeli oa boraro haeba a le teng.

7. Haeba 'na kapa ngoana'ka a hlatsa nakong ea metsotso e mashome a mararo a ho noa litlhare, kapa haeba nka ka bona li ARV ka bo tsona mahlatseng, ke tla pheta ho noa kapa ho noesa ngoana litlhare.
[ ] Mohlokomeli oa mathomo [ ] Mokuli (haeba a se a boleletsoe boemo)
[ ] Mohlokomeli oa bobeli, haeba a le teng [ ] Mohlokomeli oa boraro haeba a le teng.

8. Ke tla tla le lebokose la ka kapa la ngoana'ka la litlhare nako eohle ha ke khutlela cliniking.
[ ] Mohlokomeli oa mathomo [ ] Mokuli (haeba a se a boleletsoe boemo)
[ ] Mohlokomeli oa bobeli, haeba a le teng [ ] Mohlokomeli oa boraro haeba a le teng.

9. Bakeng sa bahlokomeli: ke itlama ka botlalo ho netefatseng hore ngoana eo ke mo hlokomelang o fumana li ARV. Haeba ke se ke sitoa ho tsoela-pele ho mo hlokomela, ke tla bolella mohlabolli oa cliniki kapele kamoo ho ka khonahalang, ele hore motho e mong e moholo a tle a hlabolloe ho etsa joalo (ho hlokomela ngoana).
[ ] Mohlokomeli oa mathomo [ ] Mokuli (haeba a se a boleletsoe boemo)
[ ] Mohlokomeli oa bobeli, haeba a le teng [ ] Mohlokomeli oa boraro haeba a le teng.

10. Bakeng sa bahlokomeli ba bana feela: kea utloisisa hore ke lokela ho khothaletsa ngoana'ka ho ba le boikarabello ba ho noa li ARV; leha ho le joalo, kea utloisisa hore bana ba lokela ho supisoa ka kotloloho ha ba e-noa kapa ba koenya li ARV, 'me ke tla ba tataisa ka hloko ho li noeng.
[ ] Mohlokomeli oa mathomo [ ] Mokuli (haeba a se a boleletsoe boemo)
[ ] Mohlokomeli oa bobeli,haeba a le teng [ ] Mohlokomeli oa boraro haeba a le teng.

11. Bakeng sa bakuli bohle, haeba ba boleletsoe boemo ba bona: kea utloisisa hore kaha li ARV hase pheko, ho ntse ho khonahala hore nka fetisetsa kokoana-hloko ho ba bang, 'me kea utloisisa hore hoa khonahala hore nka fumana mofuta o mong oa HIV, hoo ho molemong oa ka ho its'ireletsa khahlanong le ts'oaetso e 'ngoe. [ ] Mokuli (haeba a boleletsoe boemo ba hae).

E tekennoe ke: Mohlokomeli oa mathomo: ______________________
Mohlokomeli oa bobeli kapa mots'ehetsi:______________________
Mokuli (haeba a le kaholimo ho lilemo tse 12):____________________

ANNEX 9: DRUG-DRUG INTERACTIONS

(Modified from WHO Recommendations Aug 2006)

	NVP	EFV	LPV/r
Anti-mycobacterium/Antibiotics			
Rifampin	↓ NVP by 20-58%. Per Lesotho Guidelines, ok to coadminister for concurrent ATT/ART in children < 3yrs or < 10kg	↓ EFV by 25%	↓ LPV by 75% Double lpv/r dose while on concurrent ATT/ART. Do not forget to change dose once ATT completed.
Clarithromycin	None	↓clarithro by 39% Monitor efficacy or use alternative drugs	↑clarithro by 75% Adjust clarithro dose if renal impairment
Antifungals			
Ketoconazole	↑ketoconazole by 63% ↑ NVP by 15-30% DO NOT COADMINISTER	No significant change in ketoconazole or EFV levels	↑ LPV ↑ketoconazole 3x Do not exceed 200mg/day ketoconazole
Fluconazole	↑ NVP Cmax, AUC, Cmin by 100% No change in fluc level Possible increase in hepatotoxicity	No data	
Oral Contraceptives			
Ethinyl estradiol	↓ethinyl estradiol by 20% USE ALTERNATIVE OR ADDITIONAL METHODS	↑ethinyl estradiol by 37% USE ALTERNATIVE OR ADDITIONAL METHODS	↓ethinyl estradiol by 42% USE ALTERNATIVE OR ADDITIONAL METHODS
Lipid lowering agents			
Simvastatin, lovastatin	No data	↓simvastatin by 58% EFV unchanged	Potential large ↑ statin DO NOT COADMINISTER
Anti-epileptics			
Carbamazepine Phenytoin	USE WITH CAUTION	USE WITH CAUTION	↑levels when coadministerd with RTV USE WITH CAUTION
Phenytoin	USE WITH CAUTION	USE WITH CAUTION One case report showed low EFV with phenytoin	↓levels of LPV/r and ↓ levels of phenytoin DO NOT COADMINISTER
Phenobarbital	USE WITH CAUTION	USE WITH CAUTION	USE WITH CAUTION
Other			
Cisapride		↑cisapride DO NOT COADMINISTER	↑cisapride DO NOT COADMINISTER

	NVP	EFV	LPV/r
Midazolam	Potential interaction, may require dose monitoring	DO NOT COADMINISTER	DO NOT COADMINISTER
Methadone	Methadone ↓ significantly; opiate withdrawal common; increase methadone dose often necessary	Methadone ↓ 60% opiate withdrawal common; increase methadone dose often necessary	Methadone ↓ 53% opiate withdrawal may occur; may require increase in methadone dose

NRTIs and NtRTIs have very few significant intereactions with other drugs. Of note, 3TC should not be coadministered with ganciclovir.

ARV-ARV Interactions

NVP – LPV/r	NVP lowers LPV/r level<br>▪ **Dose of LPV/r must be increased to 600/150 BD (3 tabs) for treatment experienced.**<br>▪ **Standard dose of LPV/r can be used for treatment naïve.**
EFV – LPV/r	EFV lowers LPV/r level<br>▪ Dose of LPV/r must be increased to 600/150 BD (3 tabs) for treatment experienced.<br>▪ Standard dose of LPV/r can be used for treatment naïve.
LPV/r – ddI	LPV/r requires an acidic environment (with meals), ddI requires an antacid for buffering;<br>▪ **LPV/r and ddI should not be coadministered at the same time.**<br>▪ **ddI should be taken before meals (30-60 minutes) and**<br>▪ **LPV/r should be taken with the meal.**
TDF – ddI	TDF increases the levels of ddI; for body weight < 60kg, dose of ddI may be lowered<br>▪ **This combination should be avoided.**
ddI – d4t	▪ **This combination should be avoided due to overlapping toxicities**
d4t – AZT	▪ **This combination should never be used due to overlapping mechanism of action.**

If patient is taking other ARV drugs or other traditional medicines consult with HIV specialist.

ANNEX 10: HOW TO ANALYSE INDICATORS AND IDENTIFY PROBLEMS

Calculating and analysing the indicators listed in the chart below will help to monitor chronic HIV care and ART in your district.

Indicators related to ART at the district level

Indicator	Time frame for cohort	Which number or formula for calculating *(numerator / denominator)*[a]	Sources of data
1. Indicators related to patients accessing HIV care and ART			
**1a**. Number enrolled in HIV care	Last quarter	- New in last month<br>- Cumulative number of persons enrolled in HIV care	Quarterly report form—Table 1
**1b**. Number started on ART	Last quarter	- New in last month<br>- Cumulative number of persons ever started on ART at this facility	Quarterly report form—Table 2
**1b**. Number currently on ART	Cross-sectional—at end of last quarter	Total and disaggregated by sex, adult /child	Quarterly report form—Table 4
**1c**. Number of persons who are enrolled and eligible for ART but have not been started on ART	Cross-sectional—at end of last quarter	Total number enrolled and eligible but not on ART (S1 + S2)	Quarterly report form—Table 1
**1d**. Proportion of those eligible for ART in clinic who have been started on ART	Cross-sectional—at end of last quarter	Cumulative number of persons ever started on ART at this facility<br>————————<br>Total number enrolled and eligible but not on ART (S1 + S2) plus cumulative number of persons ever started on ART at this facility	Quarterly report form
**1e**. Proportion of people with advanced HIV infection receiving ARV combination therapy (UNGASS core indicator)	Cross-sectional	Number currently on ART<br>————————<br>Denominator is an estimate based on HIV prevalence and expected proportion with AIDS (not from register data)	Quarterly report form<br>————————<br>Estimate, HIV prevalence data
2. Indicators related to success of ART			
**2a**. Core indicator 9 Survival at 6, 12, 24, 36 months etc after initiation of ART	6 months on ART, 12 months on ART, 24 months, 36 months etc on ART	H + I + J<br>————<br>N	Cohort analysis form<br>————————<br>Cohort analysis form

Indicator	Time frame for cohort	Which number or formula for calculating *(numerator / denominator)*[a]	Sources of data
**2b.** Core indicator 8 Continuation of first-line ARV regimen at 6, 12 and 24 months after initiating treatment	6 months on ART, 12 months on ART, 24 months on ART	6 months on ART, 12 months on ART, 24 months, 36 months etc on ART<br>————————————<br>Persons who started 1st-line ART for the first time during the time period under consideration.	Cohort analysis form<br>————————<br>Cohort analysis form
**2c.** Proportion of people on ART at 6, 12 and 24 months whose functional status is working	6 months on ART, 12 months on ART, 24 months, 36 months etc on ART	Working<br>————————<br>Working + Ambulatory + Bedridden	Cohort analysis form
**2d.** Median CD4 and increase at 6 and at 12 months on ART compared to baseline.			Cohort analysis form
3. HIV drug resistance early warning indicators			
3a. Proportion of patients who started ART 6 or 12 months ago who picked up ARV medications 6/6 or 12/12 months.	Cross-sectional—at end of last quarter	Persons who started ART 6 or 12 months ago who picked up ARV medications 6/6 or 12/12 months.<br>————————————<br>Persons who started ART 6 or 12 months ago and are still prescribed ART at the end of the time period.	Cohort analysis form
3b. Proportion of patients with (good) adherence to ART			Patient encounter form

Other indicators for facility-level programme monitoring

Indicator	Rationale
**a. Number on cotrimoxazole, fluconazole, INH prophylaxis at end of month**	Drug supply orders
**b. Distribution of entry points of patients enrolled in HIV care**	Identifies linkages between programmes and activities
**c. Distribution of reasons for regimen substitution, switching, termination, interruption, and poor adherence**	Helps clinical team to identify and respond to poor adherence; assists with quality assurance related to regimen substitutions, switches and interruptions.
**d. Distribution of patients not yet on ART by clinical stage**	May help estimate resources to care for patients, drug supply for OI prophylaxis and treatment.
**e. Percentage of patients referred**	Monitoring referral rates may enable facilities to manage referral systems more efficiently
**f. Side effects, OIs, other problems**	Facilitates individual patient management and allows review of side effects and new OIs

Calculating indicators or other aggregated data

Agreed minimum essential data elements	What happens to the data	Indicators or other aggregated data
At baseline, 6, 12 months then yearly; disaggregated by sex and child/adult:<br><br>On ART and:<br>ALIVE<br>DEAD<br>LOST/DROP/Transfer out<br>Current regimen<br>Original 1st-line<br>Substituted to alternative 1st-line<br>2nd-line or higher<br>CD4 test results<br>Functional status<br>Regimen collected in last quarter	Transfer to ART register then to **Cohort Analysis Report**	Based on cohort analysis form, at **6, 12 months** then **yearly** and compared to **baseline**:<br><br>Indicators related to success of ART<br><br>Proportion alive and on ART/Mortality on ART<br>Proportion still on a first-line regimen<br>Proportion working, ambulatory, bedridden<br>Median or mean CD4 counts (optional)<br><br>HIV drug resistance early warning indicators:<br>Proportion switched to a second-line (or higher) regimen<br>Proportion collected ARV drugs 6/6 or 12/12 months
B.<br>When registered for HIV care<br>When medically eligible for ART<br>When medically eligible and ready for ART<br>When ART started<br>Dead before ART	Transfer to pre-ART or ART register then to **Quarterly Report**	Indicators related to patients accessing HIV care and ART:<br>Disaggregated by adult, child, sex, pregnancy status:<br>Number enrolled in HIV care: new and cumulative ever at the facility<br>Number started on ART: new and

Agreed minimum essential data elements	What happens to the data	Indicators or other aggregated data
Lost or Transfer out before ART		cumulative ever started at the facility<br>Number currently on ART at the facility<br>Not disaggregated:<br>Number eligible for ART but not yet started
C.<br>Entry point<br>Why eligible for ART<br>Reasons for:<br>Substitution within first-line<br>Switch/Substitution to or within second-line<br>Stop ART<br>Number and weeks of each ART treatment interruption<br>Pregnancy status<br>Start/stop dates of prophylaxis:<br>Co-trimoxazole<br>Fluconazole<br>INH<br>TB treatment<br>Adherence on ART	Transferred to **Pre-ART or ART Register** but used only by clinical team /district ART coordinator not transferred to quarterly report or cohort analysis	Indicators for patient and programmeme management at the facility/district level:<br>Distribution of entry points in patients enrolled in HIV care<br>Why eligible for ART: clinical only, CD4 or TLC<br>Distribution of patients not yet on ART by clinical stage<br>Distribution of reasons for substitute, switch, stop to investigate problems; whether substitutions and switches are appropriate (use in context reviewing medical officer log)<br>ART treatment interruptions:<br>Number/Proportion of patients<br>Number weeks<br>Proportion of pregnant patients linked with PMTCT interventions (or simply use to generate lists to assure linkage)<br>Number on co-trimoxazole, fluconazole, INH prophylaxis at end of quarter (for ordering prophylaxis drugs)<br>Number/Proportion of patients on both TB treatment and ART<br>% patients with good adherence to ART
D.<br>Date of each encounter<br>Weight (each visit; % wt gain or loss)<br>Adherence on CTX<br>Adherence on INH<br>Potential side effects<br>New OI, other problems<br>TB status (other than treatment or prophylaxis)<br>Referred or consulted with MD<br>Number inpatient days<br>If poor adherence on ART, reasons (coded)	**Patient Card only.**<br>Not transferred to register	Indicators for patient management at the facility level or special studies:<br><br>% patients referred to MD<br>Common side effects, OI, other problems:<br>Patients with special problems<br>Identify patients for review at clinical team meetings<br># or proportion patients hospitalized; number days<br>Reasons for poor adherence

National core indicators

These are used both for individual patient management and for medical officer or clinical mentor review on site visits. For potentially serious side effects which result in a consultation or referral, medical officer needs to put in log and do further adverse even reporting.

Tabulations for special studies

ANNEX 11:WEIGHT-BASED DOSING OF ANTI-TB FIXED-DOSE COMBINATION MEDICATIONS

Weight-based dosing of anti-TB medications using pediatric formulations (2-25 kg body weight)

Weight (kg)	Intensive phase (2 months)		Continuation phase (4 months)
	RHZ (pediatric) 60/30/150 mg	Ethambutol 100 mg	RH (pediatric) 60/60 mg
2 - 3.9 kg	1/2 tablet	1/2 tablet	1/2 tablet
4 - 5.9 kg	1 tablet	1 tablet	1 tablet
6 - 7.9 kg	1.5 tablets	1.5 tablets	1.5 tablets
8 - 10.9 kg	2 tablets	2 tablets	2 tablets
11 - 14.9 kg	3 tablets	2 tablets	3 tablets
14 - 19.9 kg	4 tablets	3 tablets	4 tablets
20-24.9 kg	5 tablets	4 tablets *(or 400mg tablet)*	5 tablets

* Follow adult dosing for children weighing 25kg or above.

Recommended treatment regimen and dosages for new adult TB cases

Phase of treatment	Drugs	Weight in kg			
		30-39	40-54	55-70	>70
Intensive phase of 2 months	(RHZE)* (150mg/75mg/400mg/275mg)	2 tabs daily	3 tabs daily	4 tabs daily	5 tabs daily
Continuation phase of 4 months	(RH) (150mg/75mg)	2 tabs daily	3 tabs daily	4 tabs daily	5 tabs daily

*Fixed-dose combination (FDC) drugs

Dosing for INH prophylaxis for children

Weight (kg)	Daily Dosage (mg) 10 mg/kg/day for 6 months	Number of 100mg Tablets of INH given daily
< 5.0	50 mg	½ tab
5.1-9.9	100 mg	1 tab
10-13.9	150 mg	1.5 tabs
14-19.9	200 mg	2 tabs
20-24.9	250 mg	2.5 tabs
>25	300 mg	3 tabs

ANNEX 12: Reference Values for Weight-for-Height and Weight-for-Length

Weight-for-Height Reference Table (WHO)

Boys weight (kg)						Girls weight (kg)				
-4SD	-3SD	-2SD	-1SD	Median	Length (cm)[a]	Median	-1SD	-2SD	-3SD	-4SD
1,7	1,9	2,0	2,2	2,4	**45,0**	2,5	2,3	2,1	1,9	1,7
1,8	1,9	2,1	2,3	2,5	**45,5**	2,5	2,3	2,1	2,0	1,8
1,8	2,0	2,2	2,4	2,6	**46,0**	2,6	2,4	2,2	2,0	1,9
1,9	2,1	2,3	2,5	2,7	**46,5**	2,7	2,5	2,3	2,1	1,9
2,0	2,1	2,3	2,5	2,8	**47,0**	2,8	2,6	2,4	2,2	2,0
2,0	2,2	2,4	2,6	2,9	**47,5**	2,9	2,6	2,4	2,2	2,0
2,1	2,3	2,5	2,7	2,9	**48,0**	3,0	2,7	2,5	2,3	2,1
2,1	2,3	2,6	2,8	3,0	**48,5**	3,1	2,8	2,6	2,4	2,2
2,2	2,4	2,6	2,9	3,1	**49,0**	3,2	2,9	2,6	2,4	2,2
2,3	2,5	2,7	3,0	3,2	**49,5**	3,3	3,0	2,7	2,5	2,3
2,4	2,6	2,8	3,0	3,3	**50,0**	3,4	3,1	2,8	2,6	2,4
2,4	2,7	2,9	3,1	3,4	**50,5**	3,5	3,2	2,9	2,7	2,4
2,5	2,7	3,0	3,2	3,5	**51,0**	3,6	3,3	3,0	2,8	2,5
2,6	2,8	3,1	3,3	3,6	**51,5**	3,7	3,4	3,1	2,8	2,6
2,7	2,9	3,2	3,5	3,8	**52,0**	3,8	3,5	3,2	2,9	2,7
2,8	3,0	3,3	3,6	3,9	**52,5**	3,9	3,6	3,3	3,0	2,8
2,9	3,1	3,4	3,7	4,0	**53,0**	4,0	3,7	3,4	3,1	2,8
3,0	3,2	3,5	3,8	4,1	**53,5**	4,2	3,8	3,5	3,2	2,9
3,1	3,3	3,6	3,9	4,3	**54,0**	4,3	3,9	3,6	3,3	3,0
3,2	3,4	3,7	4,0	4,4	**54,5**	4,4	4,0	3,7	3,4	3,1
3,3	3,6	3,8	4,2	4,5	**55,0**	4,6	4,2	3,8	3,5	3,2
3,4	3,7	4,0	4,3	4,7	**55,5**	4,7	4,3	3,9	3,6	3,3
3,5	3,8	4,1	4,4	4,8	**56,0**	4,8	4,4	4,0	3,7	3,4
3,6	3,9	4,2	4,6	5,0	**56,5**	5,0	4,5	4,2	3,8	3,5
3,7	4,0	4,3	4,7	5,1	**57,0**	5,1	4,6	4,3	3,9	3,6
3,8	4,1	4,5	4,9	5,3	**57,5**	5,2	4,8	4,4	4,0	3,7
3,9	4,3	4,6	5,0	5,4	**58,0**	5,4	4,9	4,5	4,1	3,8
4,0	4,4	4,7	5,1	5,6	**58,5**	5,5	5,0	4,6	4,2	3,9
4,1	4,5	4,8	5,3	5,7	**59,0**	5,6	5,1	4,7	4,3	3,9
4,2	4,6	5,0	5,4	5,9	**59,5**	5,7	5,3	4,8	4,4	4,0
4,3	4,7	5,1	5,5	6,0	**60,0**	5,9	5,4	4,9	4,5	4,1
4,4	4,8	5,2	5,6	6,1	**60,5**	6,0	5,5	5,0	4,6	4,2
4,5	4,9	5,3	5,8	6,3	**61,0**	6,1	5,6	5,1	4,7	4,3
4,6	5,0	5,4	5,9	6,4	**61,5**	6,3	5,7	5,2	4,8	4,4
4,7	5,1	5,6	6,0	6,5	**62,0**	6,4	5,8	5,3	4,9	4,5
4,8	5,2	5,7	6,1	6,7	**62,5**	6,5	5,9	5,4	5,0	4,6
4,9	5,3	5,8	6,2	6,8	**63,0**	6,6	6,0	5,5	5,1	4,7
5,0	5,4	5,9	6,4	6,9	**63,5**	6,7	6,2	5,6	5,2	4,7
5,1	5,5	6,0	6,5	7,0	**64,0**	6,9	6,3	5,7	5,3	4,8
5,2	5,6	6,1	6,6	7,1	**64,5**	7,0	6,4	5,8	5,4	4,9
5,3	5,7	6,2	6,7	7,3	**65,0**	7,1	6,5	5,9	5,5	5,0
5,4	5,8	6,3	6,8	7,4	**65,5**	7,2	6,6	6,0	5,5	5,1
5,5	5,9	6,4	6,9	7,5	**66,0**	7,3	6,7	6,1	5,6	5,1
5,5	6,0	6,5	7,0	7,6	**66,5**	7,4	6,8	6,2	5,7	5,2
5,6	6,1	6,6	7,1	7,7	**67,0**	7,5	6,9	6,3	5,8	5,3
5,7	6,2	6,7	7,2	7,9	**67,5**	7,6	7,0	6,4	5,9	5,4
5,8	6,3	6,8	7,3	8,0	**68,0**	7,7	7,1	6,5	6,0	5,5
5,9	6,4	6,9	7,5	8,1	**68,5**	7,9	7,2	6,6	6,1	5,5
6,0	6,5	7,0	7,6	8,2	**69,0**	8,0	7,3	6,7	6,1	5,6
6,0	6,6	7,1	7,7	8,3	**69,5**	8,1	7,4	6,8	6,2	5,7
6,1	6,6	7,2	7,8	8,4	**70,0**	8,2	7,5	6,9	6,3	5,8
6,2	6,7	7,3	7,9	8,5	**70,5**	8,3	7,6	6,9	6,4	5,8
6,3	6,8	7,4	8,0	8,6	**71,0**	8,4	7,7	7,0	6,5	5,9
6,4	6,9	7,5	8,1	8,8	**71,5**	8,5	7,7	7,1	6,5	6,0
6,4	7,0	7,6	8,2	8,9	**72,0**	8,6	7,8	7,2	6,6	6,0
6,5	7,1	7,6	8,3	9,0	**72,5**	8,7	7,9	7,3	6,7	6,1
6,6	7,2	7,7	8,4	9,1	**73,0**	8,8	8,0	7,4	6,8	6,2
6,7	7,2	7,8	8,5	9,2	**73,5**	8,9	8,1	7,4	6,9	6,3
6,7	7,3	7,9	8,6	9,3	**74,0**	9,0	8,2	7,5	6,9	6,3
6,8	7,4	8,0	8,7	9,4	**74,5**	9,1	8,3	7,6	7,0	6,4
6,9	7,5	8,1	8,8	9,5	**75,0**	9,1	8,4	7,7	7,1	6,5
7,0	7,6	8,2	8,9	9,6	**75,5**	9,2	8,5	7,8	7,1	6,5
7,0	7,6	8,3	8,9	9,7	**76,0**	9,3	8,5	7,8	7,2	6,6
7,1	7,7	8,3	9,0	9,8	**76,5**	9,4	8,6	7,9	7,3	6,7
7,2	7,8	8,4	9,1	9,9	**77,0**	9,5	8,7	8,0	7,4	6,7
7,2	7,9	8,5	9,2	10,0	**77,5**	9,6	8,8	8,1	7,4	6,8
7,3	7,9	8,6	9,3	10,1	**78,0**	9,7	8,9	8,2	7,5	6,9
7,4	8,0	8,7	9,4	10,2	**78,5**	9,8	9,0	8,2	7,6	6,9
7,4	8,1	8,7	9,5	10,3	**79,0**	9,9	9,1	8,3	7,7	7,0
7,5	8,2	8,8	9,5	10,4	**79,5**	10,0	9,1	8,4	7,7	7,1
7,6	8,2	8,9	9,6	10,4	**80,0**	10,1	9,2	8,5	7,8	7,1
7,6	8,3	9,0	9,7	10,5	**80,5**	10,2	9,3	8,6	7,9	7,2
7,7	8,4	9,1	9,8	10,6	**81,0**	10,3	9,4	8,7	8,0	7,3
7,8	8,5	9,1	9,9	10,7	**81,5**	10,4	9,5	8,8	8,1	7,4
7,9	8,5	9,2	10,0	10,8	**82,0**	10,5	9,6	8,8	8,2	7,5
7,9	8,6	9,3	10,1	10,9	**82,5**	10,6	9,7	8,9	8,2	7,5
8,0	8,7	9,4	10,2	11,0	**83,0**	10,7	9,8	9,0	8,3	7,6
8,1	8,8	9,5	10,3	11,2	**83,5**	10,9	9,9	9,1	8,4	7,7
8,2	8,9	9,6	10,4	11,3	**84,0**	11,0	10,1	9,2	8,5	7,8
8,3	9,0	9,7	10,5	11,4	**84,5**	11,1	10,2	9,3	8,6	7,9

Weight-for-Height Reference Table (WHO)

Boys weight (kg)						Girls weight (kg)				
-4SD	-3SD	-2SD	-1SD	Median	Height (cm)[a]	Median	-1SD	-2SD	-3SD	-4SD
8,4	9,1	9,8	10,6	11,5	**85,0**	11,2	10,3	9,4	8,7	8,0
8,5	9,2	9,9	10,7	11,6	**85,5**	11,3	10,4	9,6	8,8	8,0
8,6	9,3	10,0	10,8	11,7	**86,0**	11,5	10,5	9,7	8,9	8,1
8,7	9,4	10,1	11,0	11,9	**86,5**	11,6	10,6	9,8	9,0	8,2
8,9	9,6	10,4	11,2	12,2	**87,0**	11,9	10,9	10,0	9,2	8,4
9,0	9,7	10,5	11,3	12,3	**87,5**	12,0	11,0	10,1	9,3	8,5
9,1	9,8	10,6	11,5	12,4	**88,0**	12,1	11,1	10,2	9,4	8,6
9,2	9,9	10,7	11,6	12,5	**88,5**	12,3	11,2	10,3	9,5	8,7
9,3	10,0	10,8	11,7	12,7	**89,0**	12,4	11,4	10,4	9,6	8,8
9,3	10,1	10,9	11,8	12,8	**89,5**	12,5	11,5	10,5	9,7	8,9
9,4	10,2	11,0	11,9	12,9	**90,0**	12,6	11,6	10,6	9,8	9,0
9,5	10,3	11,1	12,0	13,0	**90,5**	12,8	11,7	10,7	9,9	9,1
9,6	10,4	11,2	12,1	13,1	**91,0**	12,9	11,8	10,9	10,0	9,1
9,7	10,5	11,3	12,2	13,2	**91,5**	13,0	11,9	11,0	10,1	9,2
9,8	10,6	11,4	12,3	13,4	**92,0**	13,1	12,0	11,1	10,2	9,3
9,9	10,7	11,5	12,4	13,5	**92,5**	13,3	12,1	11,2	10,3	9,4
9,9	10,8	11,6	12,6	13,6	**93,0**	13,4	12,3	11,3	10,4	9,5
10,0	10,9	11,7	12,7	13,7	**93,5**	13,5	12,4	11,4	10,5	9,6
10,1	11,0	11,8	12,8	13,8	**94,0**	13,6	12,5	11,5	10,6	9,7
10,2	11,1	11,9	12,9	13,9	**94,5**	13,8	12,6	11,6	10,7	9,7
10,3	11,1	12,0	13,0	14,1	**95,0**	13,9	12,7	11,7	10,8	9,8
10,4	11,2	12,1	13,1	14,2	**95,5**	14,0	12,8	11,8	10,8	9,9
10,4	11,3	12,2	13,2	14,3	**96,0**	14,1	12,9	11,9	10,9	10,0
10,5	11,4	12,3	13,3	14,4	**96,5**	14,3	13,1	12,0	11,0	10,1
10,6	11,5	12,4	13,4	14,6	**97,0**	14,4	13,2	12,1	11,1	10,2
10,7	11,6	12,5	13,6	14,7	**97,5**	14,5	13,3	12,2	11,2	10,3
10,8	11,7	12,6	13,7	14,8	**98,0**	14,7	13,4	12,3	11,3	10,4
10,9	11,8	12,8	13,8	14,9	**98,5**	14,8	13,5	12,4	11,4	10,4
11,0	11,9	12,9	13,9	15,1	**99,0**	14,9	13,7	12,5	11,5	10,5
11,1	12,0	13,0	14,0	15,2	**99,5**	15,1	13,8	12,7	11,6	10,6
11,2	12,1	13,1	14,2	15,4	**100,0**	15,2	13,9	12,8	11,7	10,7
11,2	12,2	13,2	14,3	15,5	**100,5**	15,4	14,1	12,9	11,9	10,8
11,3	12,3	13,3	14,4	15,6	**101,0**	15,5	14,2	13,0	12,0	10,9
11,4	12,4	13,4	14,5	15,8	**101,5**	15,7	14,3	13,1	12,1	11,0
11,5	12,5	13,6	14,7	15,9	**102,0**	15,8	14,5	13,3	12,2	11,1
11,6	12,6	13,7	14,8	16,1	**102,5**	16,0	14,6	13,4	12,3	11,2
11,7	12,8	13,8	14,9	16,2	**103,0**	16,1	14,7	13,5	12,4	11,3
11,8	12,9	13,9	15,1	16,4	**103,5**	16,3	14,9	13,6	12,5	11,4
11,9	13,0	14,0	15,2	16,5	**104,0**	16,4	15,0	13,8	12,7	11,5
12,0	13,1	14,2	15,4	16,7	**104,5**	16,6	15,2	13,9	12,8	11,6
12,1	13,2	14,3	15,5	16,8	**105,0**	16,8	15,3	14,0	12,9	11,8
12,2	13,3	14,4	15,6	17,0	**105,5**	17,0	15,5	14,2	13,0	11,9
12,3	13,4	14,5	15,8	17,2	**106,0**	17,1	15,6	14,3	13,1	12,0
12,4	13,5	14,7	15,9	17,3	**106,5**	17,3	15,8	14,5	13,3	12,1
12,5	13,7	14,8	16,1	17,5	**107,0**	17,5	15,9	14,6	13,4	12,2
12,6	13,8	14,9	16,2	17,7	**107,5**	17,7	16,1	14,7	13,5	12,3
12,7	13,9	15,1	16,4	17,8	**108,0**	17,8	16,3	14,9	13,7	12,4
12,8	14,0	15,2	16,5	18,0	**108,5**	18,0	16,4	15,0	13,8	12,6
12,9	14,1	15,3	16,7	18,2	**109,0**	18,2	16,6	15,2	13,9	12,7
13,1	14,3	15,5	16,8	18,3	**109,5**	18,4	16,8	15,4	14,1	12,8
13,2	14,4	15,6	17,0	18,5	**110,0**	18,6	17,0	15,5	14,2	12,9
13,3	14,5	15,8	17,1	18,7	**110,5**	18,8	17,1	15,7	14,4	13,1
13,4	14,6	15,9	17,3	18,9	**111,0**	19,0	17,3	15,8	14,5	13,2
13,5	14,8	16,0	17,5	19,1	**111,5**	19,2	17,5	16,0	14,7	13,3
13,6	14,9	16,2	17,6	19,2	**112,0**	19,4	17,7	16,2	14,8	13,5
13,7	15,0	16,3	17,8	19,4	**112,5**	19,6	17,9	16,3	15,0	13,6
13,8	15,2	16,5	18,0	19,6	**113,0**	19,8	18,0	16,5	15,1	13,7
14,0	15,3	16,6	18,1	19,8	**113,5**	20,0	18,2	16,7	15,3	13,9
14,1	15,4	16,8	18,3	20,0	**114,0**	20,2	18,4	16,8	15,4	14,0
14,2	15,6	16,9	18,5	20,2	**114,5**	20,5	18,6	17,0	15,6	14,1
14,3	15,7	17,1	18,6	20,4	**115,0**	20,7	18,8	17,2	15,7	14,3
14,4	15,8	17,2	18,8	20,6	**115,5**	20,9	19,0	17,3	15,9	14,4
14,6	16,0	17,4	19,0	20,8	**116,0**	21,1	19,2	17,5	16,0	14,5
14,7	16,1	17,5	19,2	21,0	**116,5**	21,3	19,4	17,7	16,2	14,7
14,8	16,2	17,7	19,3	21,2	**117,0**	21,5	19,6	17,8	16,3	14,8
14,9	16,4	17,9	19,5	21,4	**117,5**	21,7	19,8	18,0	16,5	15,0
15,0	16,5	18,0	19,7	21,6	**118,0**	22,0	20,0	18,2	16,6	15,1
15,2	16,7	18,2	19,9	21,8	**118,5**	22,2	20,1	18,4	16,8	15,2
15,3	16,8	18,3	20,0	22,0	**119,0**	22,4	20,3	18,5	16,9	15,4
15,4	16,9	18,5	20,2	22,2	**119,5**	22,6	20,5	18,7	17,1	15,5
15,5	17,1	18,6	20,4	22,4	**120,0**	22,8	20,7	18,9	17,3	15,6

[a] Using this table, length should be measured from 45 to 86,9 cm and height should be measured from 87 to 120 cm.

ANNEX 13: READY-TO-USE THERAPEUTIC FOOD (RUTF) DOSING

Weight (kg)	Sachets per Day	Sachets per Week
3.5-3.9	1.5	11
4.0-5.4	2	14
5.5-6.9	2.5	18
7.0-8.4	3	21
8.5-9.4	3.5	25
9.5-10.4	4	28
10.5-11.9	4.5	32
≥ 12	5	35
