# Supplementary material for: Beyond the facility: An evaluation of seven community-based pediatric HIV testing strategies and linkage to care outcomes in a high prevalence, resource-limited setting
Source: PLoS One. 2020 Sep 2;15(9):e0236985. doi: 10.1371/journal.pone.0236985 (PMC7467225; doi:10.1371/journal.pone.0236985)
Supplement: Attachment — Submitted filename: Response to Reviewers.docx [file pone.0236985.s005.docx]

**Reviewer #1:**

There are three main issues I feel need to be addressed before I can commit to a full review of this paper:

1. Although the authors note that M-HIT was a collaborative effort among five partners, including the Lesotho MOH, all of the authors are from CHAI. Including the IPs and MOH in production may have assisted with the two more serious issues that need to be addressed below.

**We agree with this statement, and as a result reached out to the implementing partners (including the Ministry of Health) to request their participation in authoring this paper. Two new co-authors were added, each of whom were involved in the design and supervision of the project and have assisted in clarifying your highlighted concerns.**

2. A description clearly describing the assent/consent process for the children included in this testing initiative is needed.

**This has been added in the paper under the Testing and Follow-Up Procedures subsection and have highlighted its alignment with MoH guidelines. Further clarification was provided on lines 209 - 214 to explain that both the local and international IRBs did not require written consent for the evaluation.**

3. A clear description of the eligibility criteria for testing is needed to assess the results of the paper. Were children of HIV-negative mothers tested in this program? Were children tested without first assessing the status of the mother? There are situations in which that may be appropriate. However, it needs to be clearly described as it has a big impact on how we can assess the results. Please also comment on whether the eligibility criteria for testing is in line with Lesotho MOH policy or whether there was a deviation from standard practice for the purpose of this study.

**All points have been outlined in the Testing and Follow-Up Procedures subsection (lines 155 – 182).**

**Reviewer #2:**

Summary

This article by Sindelar et al, presents a large, unique, and important dataset from Lesotho and compares several different strategies for pediatric HIV testing in community-based settings. The dataset, drawn from a large collaborative implementation program, is valuable and compares head-to-head several strategies for pediatric testing which are often presented separately. As pediatric HIV testing continues to scale up in facilities for older children outside of PMTCT settings, critically examining the comparative effectiveness of different testing strategies is essential. In addition to considering testing coverage/uptake and yield, which are often the focus of case detection strategies, this paper also considers linkage to care and ART initiation, which makes it a valuable addition. However, there are numerous large methodologic issues in data interpretation, presentation, and contextualization that make the current version of the manuscript not scientifically sound. Revising the analysis and interpretation could transform this promising dataset into important lessons for those in the field of pediatric HIV testing. I enjoyed reading this paper and would look forward to reviewing a revision.

Major essential comments

1. Concepts of uptake of testing (proportion testing among those approached), testing yield (proportion testing positive among those testing), and relative numbers testing and testing positive between different strategies and age bands are reported inconsistently throughout manuscript (methods, results, discussion) but could be revised for clarity. Below are specific notes of where these concepts seem to be misinterpreted or inconsistently applied. Suggest reporting % uptake and % yield for each strategy, along with the already presented numbers testing and testing positive for each strategy. Further suggest adjusting these metrics to be per month to address differences in time period of data collection. Current reporting and comparisons are not scientifically valid.

**We agree that uptake is an important metric, however this was not included in the study design and consequently was only measured for one of the strategies. Since we were unable to calculate uptake for all strategies, it was not included.**

**We have adopted your suggestion to report all testing volumes by monthly average and adjusted all results for each strategy. Objective 1 (lines 114-116) provides a description for the term “yield” and the presentation of all testing numbers have been revised.**

a. Lines 134-138: suggest aligning these definitions of outcomes with the aforementioned % uptake, % yield, number tested, and number tested positive. Suggest providing numerator and denominator data (as appropriate) for each outcome. “Contribution of community-based testing…” is unclear as a metric. Does this mean the proportion of all children tested within the program that came from each strategy?

**We have revised the objectives accordingly and aligned terms as suggested. In addition, we have revised three of the visuals, replacing the original Figure 1 with Table 2, the original Figure 2 with Table 3, and the original Figure 4 with Figure 2. We have included facility-based testing numbers in the greatest detail that they were available in DHIS2 (the MoH data warehouse). Tables now include a clearer description of the indicators being presented especially percentages/proportions.**

**“Contribution of community-based testing…”** **was eliminated to avoid confusion – after reconsideration the authors felt it was not a necessary or helpful metric to highlight.**

b. Line 160: unclear what testing volumes and identifications mean, but likely correspond to number tested and number testing positive; the terminology here differs from that mentioned earlier in the methods in lines 134-138. Suggest harmonizing even if my suggested terminology above is not your preferred terminology.

**Most uses of the word “identification” have been adjusted to “positive test”. Those which were not changed were given increased context for clarity. All use of the word “testing volumes” have been replaced to “testing numbers”.**

c. Lines 173-177: “MOCs tested and identified the highest number of children…contributed the smallest numbers in terms of overall children tested and identified, partly due to their shorter implementation timeframe”. Suggest adjusting all of the estimates to be per month of time, otherwise it is not a fair or valid comparison.

**Agreed, as previously mentioned all numbers presented for each testing strategy are now a monthly average (described in lines 216-217).**

d. Lines 212-215: New data are introduced here (e.g. “index case testing which conducted over 80% of all tests on children”) and reflect new metrics that are not described in the results. Is this maybe referring to uptake of testing among those tested? The proportions given in this section do not add to 100%, so don’t seem to be relative contribution to numbers tested or yield. Also see lines 234-235 for 33% and 24% numbers which seem to be related.

**Tables 2 and 3 now display the total number tested, the proportion of tests conducted in each age category, and the yields. This aligns with the written results and clarifies the proportions being presented.**

2. In addition to defining and utilizing terms consistently as mentioned above, suggest harmonizing language about comparisons that are made and what they mean, and using precise language (e.g. avoid “significant” unless in the statistical sense, avoid terms like “incredibly effective” [line 216] if there is no comparison group or reference).

a. Line 214: Suggest not using significant unless these were statistically compared; later in discussion in lines 285-286 it says it was “not possible to compare between strategies” including community and facility-based.

**All uses of the word significant were checked to ensure this referred to statistical significance. Other superfluous verbiage (i.e. “incredibly effective”) has also been removed or replaced.**

b. Line 179-180: In several places, the article describes the proportion of children who were ages 5-14 among those who tested positive. While this is not an incorrect proportion to calculate, it cannot be interpreted as it is in the discussion to reflect prevalence or yield being higher in different groups. Suggest including the age breakdown among those children tested, not just those who test positive to identify whether yield differs between the groups. If n/N and % are included for each metric, all of the important absolute and relative comparisons can be made.

**This has been included in Table 3.**

c. Lines 225-226: It’s not accurate to say that the greater number of children identified as positive using MOCs suggests that “when it is provided alongside other health services”. That would be supported by a comparison of uptake between settings, not the relative number of positives between strategies.

**This is a great point. The sentence was revised so the discussion point is backed up by the data and includes other literature for context (lines 313 – 317).**

d. Lines 239-242: It is not possible to evaluate the accuracy of the first sentence here about the contribution of CBT being relatively more than facility-based, as the authors do not present overall numbers in the results. This statement also contradicts other sections of the manuscript which say that facility based testing still has more positive children identified and higher uptake.

**This point was removed.**

3. Linkage to care and ART initiation data are wonderful to see but should have a time period and window period in their definitions (e.g. linkage to care within 90 days after HIV diagnosis, or presented for scheduled visit within 30 days). Suggest revising lines 139-146 to include both, and also define how “active” was defined in determining who was retained.

**A timebound definition for linkage to care and ART initiation was added, as well as a description of what “active” in care means and how it was documented.**

4. Contextualizing these testing strategies more accurately and precisely within the peds testing literature in terms of uptake, yield, and linkage would help influence decision-making. For example, these data support what we’ve seen in other literature that index case and targeted testing strategies tend to have higher yield than “blanket” testing models, but the discussion in lines 225-252 lacks clarity in interpretation. There are important lessons on how to choose between these strategies, but they are not clearly presented.

**Agreed. The discussion section was revised extensively to include more literature around strategies, challenges, and outcomes within the Sub-Saharan African context.**

**A column was added in Table 1 that classifies each strategy as targeted or blanket for increased clarity and ease of reference.**

5. Lines 203-204: This is a very important analysis that tests an important question of whether financial assistance/incentives/support modifies linkage to care. Given the sample size of positives in this study, it would be crucial to see the data or the point estimates and 95%CI for this analysis in order to conclude that there were no differences. Additionally, later in the paper we see that e-vouchers didn’t make it to families for a variety of reasons, some of which are reasonable to expect are random (e.g. mpesa registration issues) and others which would be expected to be related to the outcome of linkage (e.g. distance to clinic). Therefore, there is a clear potential for confounding (lines 266-267), which should be addressed and presented in this important analysis.

**We have revised this analysis in several ways. First, we took your advice and looked at the potential for confounding before and after the Test and Treat/Treat All guidelines were implemented and decided to stratify all analyses by this variable. Second, instead of using median time to ART initiation as the outcome, we switched to a time-to-event analysis and graphed Kaplan-Meier curves, along with Cox Proportional Hazards on receipt of e-voucher or not. In the Cox regression, we looked at various confounders and ended up adjusting for age, sex and district.**

Minor important comments

1. Lines 107-108: suggest revision to “No sampling procedure was implemented as this program evaluation was conducted to evaluate routine implementation”. Implementation research or implementation science is a scientific discipline that often employs sampling procedures to test hypotheses, so would not be an accurate rationale for not including a sampling process.

**This was rectified by eliminating the sentence that discussed implementation research as part of the rationale for not sampling and explaining that in order to maximize statistical power to detect effects, all available data from the program will be used rather than a sample of program data (lines 133 – 134).**

2. Table 1: Unclear what the factors were that were used to determine “high risk of HIV infection”; would be crucial to define what those are and how they were identified and operationalized. With risk screening, a major barrier is often coverage of risk assessment, so would be good to comment on this.

**Further explanation and examples were given in Table 1.**

3. Lines 162-164: Comparing time to linkage to care and ART initiation should be done using survival analysis (Cox proportional hazards regression, or some other version), not Wilcoxon’s rank sum (which is appropriate for comparing continuous time where all individuals have completed the outcome). Suggest revising the statistical test used to compare these. Did the authors perhaps intend to use a log rank test instead of a rank sum for survival data that do not have proportional hazards?

**Based on your comments we agree that a Cox proportional hazards regression would be a more robust test. Assumptions for this regression were checked and met. Methods (lines 226-229) and Results (lines 282-284) were revised accordingly.**

4. Line 164-165: For the negative binomial regression, suggest including a bit more information on the model itself and how it accounted for the varying length of time at each setting, and how it incorporated facility-based testing. Unclear as is. Additionally, suggest rechecking model results in lines 187-193, which do not reflect what would be expected based on the yield of each testing strategy.

**Thank you for pointing this out. We agree this was unclear and have added additional text in the methods section (lines 219-225). We have also re-checked the model results. As for the yields, our results do match what we'd expect - for instance, for the D2D Index strategy, our predicted number of positives is 4.95 positives per 1000 tests conducted, which is equivalent to a yield of .50%, vs the actual yield for children of 0.66% (see Table 2).**

5. Line 249: related to above, important to include in this sentence that the linkage to care intervention included provision of financial assistance, which is not standard in many settings.

**This section was completely revised for greater accuracy and outside literature was included for context.**

6. New data are introduced on lines 227-230. This is really important and valuable information that sheds light on implementation challenges. Should be included in methods and results as data, not in discussion.

**We did not include any of the qualitative survey results in the Results section as the survey was not conducted on a representative sample. We agree it contains valuable information and therefore relevent inputs were included it in the discussion to shed more light on the challenges.**

7. Glad to see the acknowledgement of need to have cost data in lines 294-295!!! This is really notably missing from the pediatric HIV testing literature and is a crucial decision-making factor for MOH. Suggest that authors consider writing a follow-up paper that documents any cost data they have, even if only number of staff members required to be employed to reach these numbers.

**Thank you, we will strongly consider this recommendation.**

Discretionary comments

1. Abstract: suggest clarifying time period for linkage and ART initiation and suggest providing N and n for the yields.

**The time period has been clarified (lines 205 – 208). Due to word limitations only Ns and yield %s have been included in the results section in Table 2; n’s can all be calculated from the information provided.**

2. Figure 1 and text do not match one another in terms of concepts being explained and relevant denominators.

**Figure 1 has been removed and replaced with Table 3.**

3. Line 198: what were the treatment guidelines for children during this period of time and could the time to ART initiation be confounded by the changes in guidelines that are mentioned in the discussion?

**The treatment guidelines and ART eligibility prior to Treat All policy implementation have been included for further clarity (171 – 173).**

4. Lines 204-206: Interesting finding about ART initiation and e-vouchers; suggest explaining why this might exist in discussion in lines 258-262.

**Analysis was redone to examine the effect of e-vouchers pre/post Treat All (Figure 2). Despite a lot of internal discussion around this no one had a clear understanding of these outcomes occurred (lines 363 – 366). Futher, an extensive literature search was conducted to add more context, but no relevant articles were found.**

5. Lines 219-220: While I agree that community-based testing likely does identify children earlier in their disease progression than facility-based testing, the present manuscript doesn’t have data to support this; suggest rephrasing this sentence to point more to other literature instead of stating as a lesson from these data.

**Agreed. This has been revised to align better with the data being presented and more literature was added.**

6. Lines 221-224: there are many studies in this space, several systematic reviews and broader synthesis papers, and global datasets and national surveys that support this point. Suggest including additional information to contextualize more broadly community testing to reach children sooner and those who slip through PMTCT cascade.

**Several specific studies around gaps in PMTCT have been added (lines 300 – 312).**

7. Line 250: who is the reference group for this?

**This sentence/reference was removed and more appropriate ones were found and discussed in its place.**

8. Line 256: There are systematic reviews about the effect of financial assistance on linkage; suggest offering a broader review of this literature to more appropriately contextualize.

**Great suggestion – this was done to the best of our ability given the scarcity of research that examines the effect of unconditional cash vouchers (most are conditional).**

9. Line 263: not possible to evaluate how recent the data in this reference are as ref is missing; were the data before the era of test and treat all? That could explain gaps. If mixed, suggest restricting to time period of post test and treat so that there shouldn’t be a gap.

**We now present all initiation and retention proportions both before and after Treat All (lines 270-276).**

**We looked extensively for research that examined the effect of Treat All policy implementation on ART initiation (timing and overall outcomes), however could not find any in the Sub-Saharan African context**. **This also would’ve been really helpful in providing more context and possible explanations around the increased effect of e-vouchers in ART initiation post-Treat All.**

10. Line 274: why were children “lost”? this is a large proportion and could influence estimates of linkage and treatment and retention.

**Lost has been defined (lines 395 – 396), as well as its causes in the Challenges subsection in lines 394-397.**

11. Line 280: ANC services? HIV services?

**Replaced ANC services with “sensitive” services (line 403).**

**Reviewer #3:**

Abstract:

• Instead of saying “Majority of children identified were…” include the exact proportion of children identified

**We have updated according to your suggestion (line 39)**.

• It may be better to put the number of children diagnosed rather than 9% of the overall number of people as the paper is about paediatric testing

**We have included the average monthly total number of positives and the overall yield for children testing positive.**

• Abstract doesn’t mention of the 7 strategies, however these where in the title of the paper?

**The abstract states that seven strategies were piloted, however due to word limitations we were not able to highlight all of them individually.**

• Was ART initiated and dispensed in the community for the duration of the follow up period? May need to clarify in the abstract where ART was dispensed. Title and abstract implies that ART was dispensed in community settings.

**This has been clarified in the abstract (line 35-36) and Testing and Follow-up Procedures subsection.**

• Line 33 are “enrolled” children the same as those children diagnosed?

**No – “enrolled” children were those that enrolled in the mHealth system after testing HIV-positive through the project. This has been better delineated in the manuscript (lines 34-36) , and the causes for children failing to be enrolled have been addressed in the Challenges section (lines 392-397).**

Introduction

1. Line 81: What year is this data from?

**The national survey was conducted in 2016 – 2017.**

2. The introduction would benefit from mention of subsequent linkage to care concerns of community-based HIV testing strategies

**We agree it needs to be addressed, but felt it fit better in our Discussion section so it could be addressed in greater detail and with additional context from other studies.**

3. The introduction outlies in great detail the barriers to testing and HIV testing gaps among children but does not bring forth any evidence of how community-based HIV testing could bridge this gap? Has previous work on community-based testing for children been done in other settings? Has this been effective?

**Great suggestion, this has been included in lines 300 – 312 and 328 - 337.**

4. The introduction provides a lot of data on ART coverage, however, is there is any data of HIV testing coverage in the age group? What is covered of early infant diagnosis in Lesotho or the region? Or PMTCT coverage? This data will be useful to give context for the testing gaps that exist if available.

**PMTCT coverage has been included in the introduction in line 98. Additional data from a national survey whose results were only released after the conclusion of the project has been added in the Discussion section to enhance contextual interpretation of the results (lines 294-299).**

5. Line 81: the prevalence is 2.1% among children of what age range?

**The age range is 0 – 14 years. This has been clarified in the manuscript and moved to the Discussion section where we felt it was more appropriate (line 296).**

6. Line 90: is there a reference to M-HIT anywhere? A protocol? This would be helpful for additional context to the study

**This is the program’s first publication, and therefore no prior literature exists on it.**

Methods

7. Is this paper presenting M-HIT or a subcomponent of M-HIT? It may be important to clarify that this paper is focusing on apart of M-HIT and clearly demarcate which part. This is not clear.

**While this paper does not include all the data that was collected for the M-HIT project, it is presenting on the entire project as a whole. The project actually started off with only 4 testing strategies, but as more strategies were added they were included in the evaluation, and thus included here in this paper. The one major component we do not include in this paper is around pregnant and lactating HIV-positive women, which we have now made clear is excluded in lines 148 - 150.**

8. Line 104 that says the testing strategies were focused on rural areas is contradictory to the rest of the section. Are Maseru and Leribe rural areas?

**The testing strategies initially focused on rural and underserved areas, however throughout the life of the project it expanded throughout the catchment areas of all government funded health facilities eliminating the sole focus on rural and underserved areas. We have removed the rural reference to not confuse the reader.**

9. Table 1: The different testing strategies are unclear in their current format. It may be useful if there was some expansion to these as the title of the paper is an evaluation of the 7 strategies and they therefore should play a major role in the paper. E.g. *Targeted testing: what was the selection criteria for a child who is high risk? Where are the venues described? Schools? Shopping complexes? Community halls?

**We have revised Table 1: a column was added that classifies each strategy as targeted or blanket for increased clarity and ease of reference and we have re-worded and/or added further context to the descriptions of the strategies to help clarify.**

*The relationship between facility index testing, door to door testing and door to door index testing is not clear. If testing is offered for all household members in facility index testing, how then do you index an individual in the same household become an index when everyone in that household has been tested already?

**The descriptions of strategies in Table 1 were updated for increased clarity.**

10. Line124: This appears as another intervention in addition to just community-based testing leading to ART initiation i.e. another motivator (economic) was provided which may have resulted in the subsequent high linkage and retention rates.

**Yes, you are correct that the mHealth component could have been another motivator. We have consequently revised our analyses to look at receipt of e-voucher and time to ART Initiation (new Figure 2) and addressed with other research in the Discussion section.**

11. Line 137: Please consider rewording to “Diagnosing children unknown HIV”. Identifying implies that the testing may have been done prior to you finding the child.

**This word selection was purposeful, as after enrollment it was discovered that a small portion of children had been previously diagnosed, however never linked to care or initiated on ART. We have added this text in at lines 163-165.**

12. Line 138: You would need a denominator to quantify this i.e. your denominator would be total number of children tested in each district (community and facility), however, this is not presented in the results?

**We have removed this objective from the manuscript to avoid confusion and after reconsideration the authors felt it was not a necessary metric to highlight.**

13. Line 145: Describing the expected ART visits in the 3 months may be useful in order for the reader to understand how many times a client would have come within the 3 months e.g. are they just supposed to come to the clinic once in the 3 months to be classified as retained?

**We have added in the timeline in lines 176-177.**

14. What is the justification of only 3-month retention given that ART is a lifelong treatment?

**While we did follow-up many patients beyond the three month mark, due to restrictions imposed by project timelines three month retention was the only unbiased time period for retention we could look at (as those that were enrolled at the end of the study did not allow longer than this time period of follow-up to examine, it would not present a true picture of 6- or 12-month retention). Although initially we did report on 6-month retention (among those who had 6-months of follow-up time in) a smaller cohort, we felt this added confusion and therefore removed it.**

1. Line 172: It would make more sense to present the yield of HIV for each testing strategy as each testing strategy was not implemented for the same amount of time. That would make a better argument for cost effectiveness of each strategy. As a stand-alone this figure is not presenting that much additional data aside from the breakdown in diagnosis by age in the different testing strategies. It may be better presented in a table that presents additional demographic information such as Gender of the children.

**Thank you for your suggestion. We agree and Tables 2 and 3 now present yield.**

2. Is additional demographic information such as gender available? This would useful to add to the paper

**We agree with you again and have disaggregated by gender in Table 3.**

3. Line 196: What happened to the other children that were not enrolled? What was the reason for non-enrolment? Do we know if they were subsequently linked to care as including the non-enrolled in the denominator would affect the linkage to care proportion significantly?

**The reasons for non-enrollment are now given in the Challenges section. We agree with you that the inclusion of this data would add great value and potentially shift the results considerably. Unfortunately, while attempts were made to track them, we were unsuccessful in finding these children.**

4. Line 204: why are there no results shown? As noted above this result is critical in understanding weather or not the e-voucher impacted linkage to care.

**We have revised this analysis and now show the results in Figure 2.**

Discussion

1. Line 248: the timely client follow-up support is not previously defined? How was this different from facility care?

**Great point—we went back and defined linkage to care to be within 3 months of their HIV-positive test date (line 206). We also have re-worded ‘timely’ to be ‘active’.**

2. Line 247: it will be best if the proportion of children lost be included in the results as mentioned above. How was “lost” defined? Is this that there was no record of them at the clinic when you followed up? If so that may be better categorised as “not linked to care”? Again, the follow up procedure between diagnosis to linkage should be clearly described in the paper.\

**We have added in the total number of HIV-positive children identified and the proportion enrolled in lines 263-264 of the Results. In the Discussion we have also clarified that “lost” means they were not enrolled into the project’s mobile application and therefore also excluded from the follow-up cohort.**
