# Supplementary material for: Beyond the facility: An evaluation of seven community-based pediatric HIV testing strategies and linkage to care outcomes in a high prevalence, resource-limited setting
Source: PLoS One. 2020 Sep 2;15(9):e0236985. doi: 10.1371/journal.pone.0236985 (PMC7467225; doi:10.1371/journal.pone.0236985)
Supplement: Attachment — Submitted filename: Response to Reviewers.docx [file pone.0236985.s006.docx]

**Reviewer #1 Comments to the Author**

Reviewer #1: Thank you for submitting this revised paper. There is important information being presented and I think that with revision, this paper may be suitable for publication. However, I feel there is one central design flaw that still needs to be addressed. It is unclear to me why the authors of this study would not have ascertained and then reported on the status of the mother of the children being tested through these community based strategies. For PITC, when children are sick and in a health facility, it makes sense for both clinical and practical reasons for the children to be tested directly, regardless of the status of the parent. This is not the case for the testing of healthy children in a community based setting. Testing children of HIV-negative mothers, as seems likely to have been done in this program, is not efficient, as the results of this study clearly show. Testing all children is appropriate for a population based survey like the PHIAs. But that is not what is being reported on here. This paper is looking at practical strategies that countries can implement for community based case finding of children. Omitting or not reporting on the status of the mother limits the generalizability and usefulness of the results presented. Unless this is addressed clearly, I cannot recommend this paper for publication.

**We apologize for the lack of clarity and specificity given in our eligibility criteria. Children of HIV-negative mothers were not tested under the M-HIT project, and as such we have revised lines 155-160 to clarify.**

**Reviewer #2: Summary of revised paper**

This article from Sindelar et al has been substantially and meaningfully revised to address reviewer comments. Analyses have been redone and presented in clearer ways and more context has been given. Despite all of these well-conducted revisions, some major internal inconsistencies remain in this paper that would need to be addressed for it to be scientifically accurate. Additionally, there are several cases where easily identifiable and very relevant literature was not able to be found by the authors, which diminishes the sense that a careful detailed eye was applied to other areas of revision within the paper.

Major essential comments

1. Table 2 does not include the number of children who tested positive, which makes it impossible to check many of the subsequent analyses. Below are specific items to address to allow the paper to be accurately evaluated:

a. The authors’ revised “Objectives” section is great. However, the columns in Table 2 do not match their objective, which states that comparison will be done between # tested, # tested positive, and proportion tested positive (yield). The table shows a new proportion that was not previously described and is not well interpreted in the text, and fails to include the number who tested positive in each of the categories. Suggest adding this column. The authors suggest this was omitted because of word count or space restrictions; in this case, I’d suggest removing the column showing the proportion in the column to the left of yield.

**We were worried the table would be too busy and had erroneously thought the reader could calculate the number who tested positive. You’re correct that it is difficult to do this as we are showing averages in the table with numbers that are not whole integers, and because these numbers are small rounding does play a factor. As such, we have revised both Tables 2 and 3 to include the number testing HIV-positive. We left in the proportion tested (of each age group), as it was crucial to the project to determine if these strategies were successful at reaching substantial proportions of children. This was added to Objective 1 to better highlight its importance to the study.**

b. Related to the previous comment, when I tried to back calculate the number of positives in each of the strata (as suggested by the authors in their response letter), the presented yields did not make sense. For example, the stated yield for D2D Index testing is 0.66% with a denominator of 229 children; if 1 child had tested positive, the yield would have been 0.437%; if 2 had tested positive, it would have been 0.873%; neither of these is the reported 0.66%. I checked several numbers across this table and all suggested either incorrect math or rounding errors. Suggest adding the column with the number of people who tested positive and then rechecking all proportions listed in the paper.

**Thank you for pointing this out to us—you are correct, and we did make corrections to the “All M-HIT” age and sex yields, and as mentioned above add in the numbers testing HIV-positive to Tables 2 and 3.**

2. It is not possible to fully evaluate the accuracy of the negative binomial regression that the authors present. Despite the changes made in this revision, I still have concerns about the accuracy of this analysis. While the authors tried to address this concern in the revision by noting for D2D Index that 4.95 translates to 0.50, which is close to the 0.66% presented, that logic does NOT hold for the Targeted (2.23 vs 0.63%) and the Facility Index testing (1.34 vs 0.66%). Additionally, if this model was used to adjust for differences in time spent using each approach, we would not expect these numbers to be comparable. Overall, this statistical analysis cannot be evaluated for accuracy and appropriateness until the number of positive individuals is included in Table 2 and the proportions of yield are revised to be accurate.

**We have added in the number of positive individuals to Tables 2 and 3 and hope that sheds better light on how we modeled the number of HIV positive children for all testing strategies—M-HIT and facility-based testing. In addition, we would be happy to share our Stata do-file if that would help the reviewer feel more comfortable with this analysis; we consulted an epidemiologist and she confirmed the methods were sound. The model was run to compare the seven community-based strategies with facility-based testing. In doing so, we needed to account for the different number of months each strategy was implemented, the different numbers tested in each strategy, and the overlapping spatial dimensions (multiple strategies could have been implemented in the same community councils, a sub-division of Lesotho’s districts). Finally, the example we gave was meant to reconcile a previous concern about the yields being very different in the model—we had only wanted to show that the yields were in fact similar.**

3. The authors suggest that despite extensive literature review, there were no studies identified that addressed the impact of incentives on linkage to care. I’ve provided a number of articles that were identified using the search terms, “incentive linkage to care HIV” in PubMed. Even if these are not perfectly aligned to what the authors were searching for, it is inappropriate to present the results of this incentive analysis without contextualizing it in any of the incentives literature:

A Conditional Economic Incentive Fails to Improve Linkage to Care and Antiretroviral Therapy Initiation Among HIV-Positive Adults in Cape Town, South Africa.
Maughan-Brown B, Smith P, Kuo C, Harrison A, Lurie MN, Bekker LG, Galárraga O.
AIDS Patient Care STDS. 2018 Feb;32(2):70-78. doi: 10.1089/apc.2017.0238.

Financial Incentives for Linkage to Care and Viral Suppression Among HIV-Positive Patients: A Randomized Clinical Trial (HPTN 065).
El-Sadr WM, Donnell D, Beauchamp G, Hall HI, Torian LV, Zingman B, Lum G, Kharfen M, Elion R, Leider J, Gordin FM, Elharrar V, Burns D, Zerbe A, Gamble T, Branson B; HPTN 065 Study Team.
JAMA Intern Med. 2017 Aug 1;177(8):1083-1092. doi: 10.1001/jamainternmed.2017.2158.

Economic strengthening for HIV testing and linkage to care: a review of the evidence.
Swann M.
AIDS Care. 2018;30(sup3):85-98. doi: 10.1080/09540121.2018.1476665. Review

[https://www.ncbi.nlm.nih.gov/pmc/articles/PMC4699403/](https://linkprotect.cudasvc.com/url?a=https%3a%2f%2fwww.ncbi.nlm.nih.gov%2fpmc%2farticles%2fPMC4699403%2f&c=E,1,_ivBdcEM6xwd1y8JMBFQlnl8ZqBSIIEiw_gAqX5MiKiWA3M5NCCGbz0Ytqa5waUD-DFoxZmauHP8zA6TWlPU_4UatFHhr7uiJuUpVAgZ5EnX67q5fOTj&typo=1)

Economic strengthening for HIV testing and linkage to care: a review of the evidence.
Swann M.
AIDS Care. 2018;30(sup3):85-98. doi: 10.1080/09540121.2018.1476665. Review.


A combination intervention strategy to improve linkage to and retention in HIV care following diagnosis in Mozambique: A cluster-randomized study.
Elul B, Lamb MR, Lahuerta M, Abacassamo F, Ahoua L, Kujawski SA, Tomo M, Jani I.
PLoS Med. 2017 Nov 14;14(11):e1002433. doi: 10.1371/journal.pmed.1002433. eCollection 2017 Nov.

High-Yield HIV Testing, Facilitated Linkage to Care, and Prevention for Female Youth in Kenya (GIRLS Study): Implementation Science Protocol for a Priority Population.
Inwani I, Chhun N, Agot K, Cleland CM, Buttolph J, Thirumurthy H, Kurth AE.
JMIR Res Protoc. 2017 Dec 13;6(12):e179. doi: 10.2196/resprot.8200.

Investigating interventions to increase uptake of HIV testing and linkage into care or prevention for male partners of pregnant women in antenatal clinics in Blantyre, Malawi: study protocol for a cluster randomised trial.
Choko AT, Fielding K, Stallard N, Maheswaran H, Lepine A, Desmond N, Kumwenda MK, Corbett EL.
Trials. 2017 Jul 24;18(1):349. doi: 10.1186/s13063-017-2093-2.

Voucher incentives improve linkage to and retention in care among HIV-infected drug users in Chennai, India.
Solomon SS, Srikrishnan AK, Vasudevan CK, Anand S, Kumar MS, Balakrishnan P, Mehta SH, Solomon S, Lucas GM.
Clin Infect Dis. 2014 Aug 15;59(4):589-95. doi: 10.1093/cid/ciu324. Epub 2014 May 6.

**Thank you for the tremendous effort you made to provide relevant and valuable research to adequately bulk up the incentives discussion. To clarify, we did not suggest that such papers did not exist (as was stated on lines 371-382 of the second submission), however we felt that because of the inherent difference in conditional and unconditional incentives, it could be misleading and inaccurate to compare their outcomes. We understand your concerns around providing a broader contextualization of these outcomes for the audience and have expanded this discussion with additional literature as suggested.**

Minor

1. In table 1, D2D Index testing should be relabeled as a targeted strategy. Any strategies that use an index to identify individuals for testing, or apply any other approach for risk stratification among individuals or risk to influence selection of sites is targeted, not blanket testing.

**Change made in Table 1.**

2. The authors clarified that “active” in care is defined as having one or more visits after ART initiation. It is not clear whether this took into consideration the number of visits that one SHOULD have had. For example, a child who had 2 visits after ART initiation, but was expected based on time elapsed to have had 9, should likely be classified as lost to follow up or not retained, but using the authors’ described definition would still be considered active. This approach systematically overestimates retention, which is not scientifically appropriate.

**There are several reasons we utilized this terminology. It was previously reported that no standard definition for retention in care exists [1], and therefore we purposefully chose a simplified definition of ‘active’. Although this too could be defined differently, we were somewhat limited by our data. According to Lesotho’s MoH guidelines, all children should have at least one ART visit by 3-months, so for the sake of consistency with their reporting (as was done as much as possible in the M-HIT study) we adopted this definition. Finally, we do not believe that the opposite of active is lost-to-follow-up, nor that active means the same thing as ‘retained’, and again purposefully did not use these terms.**

**That being said, we understand your concern around utilizing the word ‘active’ to describe this outcome as it can mislead the reader. In an effort to be as clear as possible and use streamlined terminology, we found wide adoption (including the WHO) of the more inclusive term “engaged in care,” which we believe is a more accurate description of the outcomes being presented in this research [2-5].**

3. The analysis in lines 274-6 should include a statistical comparison and p-value.

**We have conducted a Wilcoxon rank-sum test and added the p-value.**

4. There is mismatch between Table 2 and Figure 1. Figure 1 still has “Facility-based” as a category, which is not presented in Table 2 and seems strangely high.

**Figure 1 should be compared to Table 3, as both of those show data on the ‘Children’ age-group. We have included facility-based testing in Table 3 where we could. As described above, Figure 1 is modeled data, and the purpose was to include this important comparison to the facility-based testing. We would expect facility-based testing to find the highest number of HIV-positive children as the traditional venue where children are tested, particularly those who are ill and symptomatic. Ideally, we would have included facility-based data in Table 2 as well, however the MoH age stratifications do not provide the breakdown between adults and adolescents.**

5. There is still new data presented in the discussion of this manuscript, which should not happen in a scientific manuscript. The authors state that the data were not presented in the results section because the sample used in the survey was not representative; this is not a reasonable reason for introducing new data in the discussion, unless those data have already been published elsewhere (in which case they should be referenced).

**This section has been revised so that it no longer cites results from the survey and instead frames it as a discussion of all potential benefits of the mobile outreach clinics.**

6. The authors were not able to find any papers about linkage to care in the pre or post test and treat era. Here are some papers for consideration from PubMed. Also consider finding the pediatric specific paper from the SEARCH trial. Some include adult populations, but can be included to contextualize:

Predictors of timely linkage-to-ART within universal test and treat in the HPTN 071 (PopART) trial in Zambia and South Africa: findings from a nested case-control study.
Sabapathy K, Mubekapi-Musadaidzwa C, Mulubwa C, Schaap A, Hoddinott G, Stangl A, Floyd S, Ayles H, Fidler S, Hayes R; HPTN 071 (PopART) study team.
J Int AIDS Soc. 2017 Dec;20(4). doi: 10.1002/jia2.25037.

High levels of retention in care with streamlined care and universal test and treat in East Africa.
Brown LB, Havlir DV, Ayieko J, Mwangwa F, Owaraganise A, Kwarisiima D, Jain V, Ruel T, Clark T, Chamie G, Bukusi EA, Cohen CR, Kamya MR, Petersen ML, Charlebois ED; SEARCH Collaboration.
AIDS. 2016 Nov 28;30(18):2855-2864.

Only adults: Understanding the Time Needed to Link to Care and Start ART in Seven HPTN 071 (PopART) Study Communities in Zambia and South Africa.
Seeley J, Bond V, Yang B, Floyd S, MacLeod D, Viljoen L, Phiri M, Simuyaba M, Hoddinott G, Shanaube K, Bwalya C, de Villiers L, Jennings K, Mwanza M, Schaap A, Dunbar R, Sabapathy K, Ayles H, Bock P, Hayes R, Fidler S; HPTN 071 (PopART) study team.
AIDS Behav. 2019 Apr;23(4):929-946. doi: 10.1007/s10461-018-2335-7.

**Thank you, we have further contextualized this discussion point as suggested.**

7. The reasons given for no voucher being received are related to distance, which could also be associated with linkage, serving as a confounder. This needs to be mentioned in the limitations section and interpreted.

**The reasons we gave for no e-voucher being received were in lines 437-438: “Additionally, some children did not receive an e-voucher due to network failures in remote locations, client errors in M-Pesa registration, or general system errors.” Distance to the health facility was irrelevant as all e-vouchers were sent via mobile phone and the cash could be collected through any m-pesa agent nationwide. The money could also be transferred to a transport operator electronically if no agents were near.**

**References**

1. Rollins N, Essajee S, Bellare N, Doherty M, Hirnschall G. Improving Retention in Care Among Pregnant Women and Mothers Living With HIV. JAIDS Journal of Acquired Immune Deficiency Syndromes. 2017;75:S111-S114.
2. World Health Organization. Global Health Sector Strategy on HIV: 2016–2021. Geneva: World Health Organization; 2016. Available from: https://apps.who.int/iris/bitstream/handle/10665/246178/WHO-HIV-2016.05-eng.pdf
3. Bengtson A, Kumwenda W, Lurie M, Klyn B, Owino M, Miller W et al. Improving Monitoring of Engagement in HIV Care for Women in Option B+: A Pilot Test of Biometric Fingerprint Scanning in Lilongwe, Malawi. AIDS and Behavior. 2019;24(2):551-559.
4. Cheever L. Engaging HIV-Infected Patients in Care: Their Lives Depend on It. Clinical Infectious Diseases. 2007;44(11):1500-1502.
5. Gardner E, McLees M, Steiner J, del Rio C, Burman W. The Spectrum of Engagement in HIV Care and its Relevance to Test-and-Treat Strategies for Prevention of HIV Infection. 2020.
