# Supplementary material for: Beyond the facility: An evaluation of seven community-based pediatric HIV testing strategies and linkage to care outcomes in a high prevalence, resource-limited setting
Source: PLoS One. 2020 Sep 2;15(9):e0236985. doi: 10.1371/journal.pone.0236985 (PMC7467225; doi:10.1371/journal.pone.0236985)
Supplement: Attachment — Submitted filename: Response to Reviewers.docx [file pone.0236985.s007.docx]

**Reviewer #1:** Thanks for the response to my concern. The authors have stated that “Children of HIV-negative mothers were not tested under the M-HIT project”, which is a really important statement. What it implies is that the M-HIT project had a system for screening for the exposure status of children and then directing their testing based on that screening process. This statement implies that they had a way of determining whether the mother was HIV infected or HIV-negative. The authors need to provide more detail on this as it would be crucial for other countries and programs to understand how they managed this as they design their own community testing programs. The actual data collection form and SOP should be provided. It would also be essential if the authors have it to provide information on how many mothers were screened, how many were infected versus negative, as well as how many children had a deceased mother or a status unknown mother.

I am still concerned about whether in fact all or even a majority of the children were HIV-exposed and eligible for testing and that concern is based on the numbers. According to the text an average of 2433 were done per month over 30 months which would total to 72,990 tests in 2 districts in Lesotho. This number is discrepant to the MHIT Testing Strategies Dataset provided which has a total of 406,983 test (46% in children would give roughly 187,000. I didn’t go through to sort it to get the exact number, but the authors need to double-check this discrepancy). Regardless of which number is correct, both would be very high for a testing strategy that was restricted to HIV-exposed children. To give a sense of expected numbers see exercise below utilizing numbers from Spectrum. According to this exercise, there are only roughly 150,000 exposed children in all of Lesotho. Using the numbers given in the text (72,990), the authors propose that they have tested half this number in just two districts. The yield seen in this study is less than what would be expected if you just tested all children in the general population at random, which is not what the authors have stated was done in this program.

Numbers for Lesotho from Spectrum 2018 and 2019

General Population= 2,049,311
Population <15= 651,743
Estimated <15 exposed to HIV= 151,649 (calculated as mothers needing PMTCT per year x .95x 15 years)
Estimated <15 HIV-infected= 12,134
Prevalence<15= 12,143/651,743=
<15 HIV+ on ART= 8,499
<15 HIV+ not on ART= 3,635

Expected Yield General Population Testing= 3,635/651,743= 0.6%
Expected Yield HIV Exposed Restricted Testing= 3635/151,649= 2.4%

Perhaps, I have made an error in my calculations. I would love to hear back from the authors on this. I must again emphasize the primary importance of describing clearly how the children in this study came to be tested for the downstream interpretation of these results and the generalizability of the findings to other programs and countries.

**Author Response**

**First, we really want to thank you for your thorough examination of our research and the additional effort you put forth in understanding its context. It is also our primarily objective to inform future programs, and we are grateful for the clarity your questions are bringing this paper.**

**We misconstrued the eligibility terms for children (>18 months) by conveying that it included only those with a mother who is positive or has an unknown status. This statement is only true for infants (<18 months). The majority of the children (>18 months) tested through M-HIT were not exposed; however, they were eligible per the Ministry of Health guidelines. The guidelines state that all individuals with an unknown status are eligible for testing.  For those older than 18 months, all were offered testing, regardless of maternal status and exposure history.  There is one algorithm that is inclusive of all children >18 months, adolescents, and adults with additional conditions for adolescents and adults as already outlined in the paper. Lesotho has always embraced an inclusive ‘Know Your Status’ philosophy for testing and we apologize for our error in communicating that critical piece of information.**

**At the time of M-HIT implementation, additional risk-based stratification was only used for re-testing protocols to determine frequency, not for initial testing.  When determining eligibility, healthcare workers examined the child’s health book to identify whether their status was known or unknown.**

**It is important to note that both index strategies did not follow the MOH eligibility requirements. In the index strategies, all household members were offered HTS as these individuals were thought to be at higher risk of infection. The manuscript has been updated to reflect all of these HTS eligibility requirements.**

**We have also provided all MoH HTS algorithms in Annex 1 – 3 of this document (0-9 months, 9-18 months, and children/adolescents/adults). These were taken directly from the Lesotho 2013 MOH Guidelines. This full document will also be uploaded as part of this submission process for your reference.**

**Reviewer #2**: Thank you for making revisions to this paper. All but the following two points were sufficiently addressed in the most recent submission.

The modeled number of positive children analysis is still not clear, even when compared to the newly presented number of children tested. I suggest removing it from the manuscript as it does not add additional insight about how to prioritize testing strategies.

**Author Response**

**We have removed, per your suggestion.**

While the evouchers were sent by mobile phone, and therefore getting to the health facility was not a problem in receiving money, the authors still state that "remote locations" was associated with network failures and accounted for some evouchers not being received; "remote locations" is also likely associated with distance to a clinic and therefore likelihood of linkage to care. Given this potential for confounding, a note should be added to the limitations to qualify this.

**Thank you for clarifying this further; we see your point now and have added in a sentence in line 400 – 402.**

**Reviewer #4:** 1) It is unclear how “positive yield” was defined and calculated. Take Table 2 Adults category as an example. In the first row, Tested positive (30.80) / Tested (1182) = 2.60%, which is the positive yield shown in the table. But how the All M-HIT positive yield was calculated, as 17.40 / 2327 = 0.75%, which does not equal to 3.73%.

**Author Response**

**Thank you for catching this error: while for the total number tested I was taking the sum of each strategy, for the total number positive I was taking the overall average—I have revised the table so that both of these numbers are now the overall average for all strategies, for the average number of months each strategy was implemented for (the number of months each strategy was implemented for is in Table 1). The yields are now reflective of positive/tested. For Adults, you will now see 121.82/3265=3.73%. Note that I have updated the “All M-HIT” row in Table 3 to align to the same methods as Table 2.**

**Annex**

Annex 1: HTS algorithm for infants 0-9 months

Annex 2: HTS algorithm for infants 9-18 months

Annex 3: HTS algorithm for individuals >18 months
