# Supplementary material for: Beyond the facility: An evaluation of seven community-based pediatric HIV testing strategies and linkage to care outcomes in a high prevalence, resource-limited setting
Source: PLoS One. 2020 Sep 2;15(9):e0236985. doi: 10.1371/journal.pone.0236985 (PMC7467225; doi:10.1371/journal.pone.0236985)
Supplement: Attachment — Submitted filename: Reviewer Comments Plos.docx [file pone.0236985.s004.docx]

**Reviewer Comments: CDC**

**Abstract:**

- Instead of saying “Majority of children identified were…” include the exact proportion of children identified
- It may be better to put the number of children diagnosed rather than 9% of the overall number of people as the paper is about paediatric testing
- Abstract doesn’t mention of the 7 strategies, however these where in the title of the paper?
- Was ART initiated and dispensed in the community for the duration of the follow up period? May need to clarify in the abstract where ART was dispensed. Title and abstract implies that ART was dispensed in community settings.
- Line 33 are “enrolled” children the same as those children diagnosed?

**Introduction**

1. Line 81: What year is this data from?
2. The introduction would benefit form mention of subsequent linkage to care concerns of community-based HIV testing strategies
3. The introduction outlies in great detail the barriers to testing and HIV testing gaps among children but does not bring forth any evidence of how community-based HIV testing could bridge this gap? Has previous work on community-based testing for children been done in other settings? Has this been effective?
4. The introduction provides a lot of data on ART coverage, however, is there is any data of HIV testing coverage in the age group? What is covered of early infant diagnosis in Lesotho or the region? Or PMTCT coverage? This data will be useful to give context for the testing gaps that exist if available.
5. Line 81: the prevalence is 2.1% among children of what age range?
6. Line 90: is there a reference to M-HIT anywhere? A protocol? This would be helpful for additional context to the study

**Methods**

7. Is this paper presenting M-HIT or a subcomponent of M-HIT? It may be important to clarify that this paper is focusing on apart of M-HIT and clearly demarcate which part. This is not clear.
8. Line 104 that says the testing strategies were focused on rural areas is contradictory to the rest of the section. Are Maseru and Leribe rural areas?
9. Table 1: The different testing strategies are unclear in their current format. It may be useful if there was some expansion to these as the title of the paper is an evaluation of the 7 strategies and they therefore should play a major role in the paper. E.g. *Targeted testing: what was the selection criteria for a child who is high risk? Where are the venues described? Schools? Shopping complexes? Community halls?

*The relationship between facility index testing, door to door testing and door to door index testing is not clear. If testing is offered for all household members in facility index testing, how then do you index an individual in the same household become an index when everyone in that household has been tested already?

10. Line124: This appears as another intervention in addition to just community-based testing leading to ART initiation i.e. another motivator (economic) was provided which may have resulted in the subsequent high linkage and retention rates.
11. Line 137: Please consider rewording to “Diagnosing children unknown HIV”. Identifying implies that the testing may have been done prior to you finding the child.
12. 138: You would need a denominator to quantify this i.e. your denominator would be total number of children tested in each district (community and facility), however, this is not presented in the results?
13. Line 145: Describing the expected ART visits in the 3 months may be useful in order for the reader to understand how many times a client would have come within the 3 months e.g. are they just supposed to come to the clinic once in the 3 months to be classified as retained?
14. What is the justification of only 3-month retention given that ART is a lifelong treatment?

**Results**

1. Line 172: It would make more sense to present the yield of HIV for each testing strategy as each testing strategy was not implemented for the same amount of time. That would make a better argument for cost effectiveness of each strategy. As a stand-alone this figure is not presenting that much additional data aside from the breakdown in diagnosis by age in the different testing strategies. It may be better presented in a table that presents additional demographic information such as Gender of the children.
2. Is additional demographic information such as gender available? This would useful to add to the paper
3. Line 196: What happened to the other children that were not enrolled? What was the reason for non-enrolment? Do we know if they were subsequently linked to care as including the non-enrolled in the denominator would affect the linkage to care proportion significantly?
4. Line 204: why are there no results shown? As noted above this result is critical in understanding weather or not the e-voucher impacted linkage to care.

**Discussion**

1. Line 248: the timely client follow-up support is not previously defined? How was this different from facility care?
2. Line 247: it will be best if the proportion of children lost be included in the results as mentioned above. How was “lost” defined? Is this that there was no record of them at the clinic when you followed up? If so that may be better categorised as “not linked to care”? Again, the follow up procedure between diagnosis to linkage should be clearly described in the paper.
